# Versatile Chemical Recycling Strategies: Value‐Added Chemicals from Polyester and Polycarbonate Waste

**DOI:** 10.1002/cssc.202200255

**Published:** 2022-02-23

**Authors:** Jack M. Payne, Muhammad Kamran, Matthew G. Davidson, Matthew D. Jones

**Affiliations:** ^1^ Centre for Sustainable and Circular Technologies University of Bath Claverton Down Bath BA2 7AY United Kingdom; ^2^ Department of Chemistry University of Bath Claverton Down Bath BA2 7AY United Kingdom

**Keywords:** chemical upcycling, green chemistry, homogeneous catalysis, polycarbonates, polyesters

## Abstract

Zn^II^‐complexes bearing half‐salan ligands were exploited in the mild and selective chemical upcycling of various commercial polyesters and polycarbonates. Remarkably, we report the first example of discrete metal‐mediated poly(bisphenol A carbonate) (BPA‐PC) methanolysis being appreciably active at room temperature. Indeed, Zn(**2**)_2_ and Zn(**2**)Et achieved complete BPA‐PC consumption within 12–18 mins in 2‐Me‐THF, noting high bisphenol A (BPA) yields (*S_BPA_* =85–91 %) within 2–4 h. Further kinetic analysis found such catalysts to possess *k_app_* values of 0.28±0.040 and 0.47±0.049 min^−1^ respectively at 4 wt%, the highest reported to date. A completely circular upcycling approach to plastic waste was demonstrated through the production of several renewable poly(ester‐amide)s (PEAs), based on a terephthalamide monomer derived from bottle‐grade poly(ethylene terephthalate) (PET), which exhibited excellent thermal properties.

## Graphical abstract

**Full circle**: Zn^II^‐complexes bearing half‐salan ligands facilitate the mild and selective degradation of various commercial polyesters and polycarbonates into value‐added products (green solvents and chemical building blocks). We report the first example of discrete metal‐mediated poly(bisphenol A carbonate) methanolysis being appreciably active at room temperature, whilst the production of several renewable poly(ester‐amides)s demonstrates a completely circular PET waste upcycling approach.

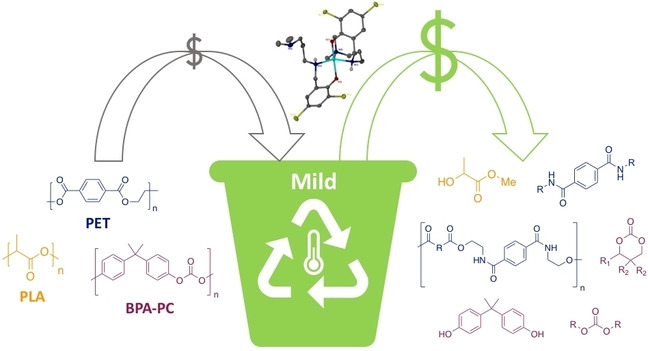

## Introduction

The “Plastic Age” has delivered unparalleled prosperity in virtually every facet of society from healthcare and transport to agriculture and communications.^[1]^ However, rapid modernization has highlighted several major drawbacks associated with a linear plastics economy at scale (367 million tonnes in 2020).^[2]^ Firstly, production is unsustainable based on a depleting fossil reserve, equating to *ca*. 99 % of all processed plastics.^[3]^ Secondly, short‐sighted product design has inadvertently rendered traditional plastics (e. g. robust and durable) a persistent environmental pollutant. This, coupled with irresponsible handling at end‐of‐life (EoL), underpin plastic pollution, exemplified by ocean plastics.^[4]^ To this end, renewable and/or biodegradable alternatives, such as poly(lactic acid) (PLA) and poly(ethylene furanoate) (PEF), have been developed.[^3^, ^5^] Whilst compostable materials represent a possible solution to system leakage, a linear approach fails to capture embedded material value. This is particularly problematic in sectors dominated by single‐use plastics, such as packaging.^[3]^ Thus, a sustainable and green plastic economy relies on transitioning towards a circular model, with recycling a promising enabler.^[6]^ Industrially, mechanical recycling is primarily used, accounting almost entirely for Europe's (EU 28+2) packaging recycling rate in 2018.^[7]^ However, this method is limited by eventual material downcycling, necessitating product repurposing to lower value applications, which creates uncertainty surrounding long‐term material value retention.[^6a^, ^8^] Conversely, chemical recycling enables product quality to be preserved over an infinite number of cycles. Moreover, scope for both monomer recovery and derivatization of platform/value‐added chemicals provides additional economic benefits to industry.^[9]^ However, barriers to adoption within industry remain, which include high CAPEX/operating costs and energy intensity.[^1c^, ^9g^] There is therefore a clear opportunity to develop sustainable chemical recycling strategies to overcome such challenges, with catalysis central to such innovation. Polymer classes relevant to this study include polyesters (e. g. PLA and poly(ethylene terephthalate) (PET) and polycarbonates (e. g. poly(bisphenol A carbonate) (BPA‐PC)). PLA recycling methods have recently been reviewed and include strategies such as pyrolysis, hydrogenation and hydrosilylation.[^1c^, ^6a^, ^10^] Metal‐mediated transesterification is most pertinent herein, although literature examples remain scarce, particularly relative to lactide polymerization.[^11^, ^12^] Given the proliferation of plastic pollution, it is imperative future innovation is not limited to emerging plastics and encompasses established commercial products, such as PET and BPA‐PC. Additionally, the latter is also a significant source of bisphenol A (BPA), a damaging xenostrogenic pollutant.[^1a^, ^13a^] Both are used in a diverse range of applications, notably packaging and engineering, and are largely amenable to chemical recycling strategies.[^1c^, ^13^] PET glycolysis is a commonly used method, often employing a metal acetate transesterification catalyst to furnish bis(2‐hydroxyethyl)terephthalate (BHET), which can be repolymerized or redirected for upcycling.[^1c^, ^13d^] PET aminolysis has also been reported, yielding value‐added terephthalamides for use in the production of additives and high‐performance materials.[^13d^, ^14^] Catalysts for PET alcoholysis and glycolysis have been widely investigated, although discrete metal‐based examples remain rare.[^12f^, ^12g^, ^15^] Comparatively, BPA‐PC remains vastly underexplored and is limited almost exclusively to the use of organocatalysts.[^13a^, ^16^] Notable examples include TBD‐ and DBU‐catalysed methanolysis reported by Do et al.^[16a]^ and Quaranta et al.^[16b]^ respectively. Traditionally, such systems rely on elevated temperatures (≥70 °C), high catalyst loadings (≥5 mol%)figr3 and/or prolonged reaction times.^[13a]^ However, simple alkali‐metal catalysed methanolysis using NaOH has been shown to be an efficient alternative.^[17]^ Indeed, Liu et al.^[17a]^ reported over 95 % BPA yield within 35 min at 40 °C in THF {m(BPA‐PC):m(MeOH)=1 : 1, 2 wt% NaOH}. Alongside BPA, organic carbonates (e. g. dimethyl carbonate (DMC)) are also produced. Such chemicals are commercially important with a variety of uses, for example as green solvents, battery electrolytes and chemical building blocks.[^13a^, ^18^] Whilst metal‐mediated degradation is a possible solution to such crucial challenges in the field, a clear industry appetite remains for cheap and environmentally benign alternatives through judicial catalyst design.

Herein, we report a series of Zn^II^‐complexes bearing half‐salan ligands and their application to the mild and selective degradation of several commercial polyesters and polycarbonates. Various strategies (e. g. alcoholysis, glycolysis and aminolysis) were used to obtain a diverse range of value‐added chemicals. A completely circular upcycling approach to plastic waste is demonstrated through the production of several renewable poly(ester‐amide)s based on a terephthalamide monomer derived from bottle‐grade PET.

## Results and Discussion

We have previously shown ligand flexibility and hydrogen bond donors to dramatically influence the activity of Zn^II^‐complexes for polyester degradation.[^12g^, ^12h^, ^12i^] Thus, we sought to design new industrially relevant ligands that combine such adventitious attributes. A range of monoanionic half‐salan ligands (**1‐6**H) were prepared *via* a facile two‐step process (Scheme 1) and characterized by multinuclear NMR spectroscopy and high resolution ESI‐MS (Figures S1–S18). A variety of heteroleptic Zn**L^HS^** Et monomers and homoleptic Zn**L^HS^**_2_ complexes were formed upon reaction with ZnEt_2_, where **L^HS^** denotes the half‐salan ligand (Scheme 1). Complexes were isolated and characterized in solution (Figures S19–S44) and in the solid‐state (Figure 1 and Figure 2).

**Scheme 1:**
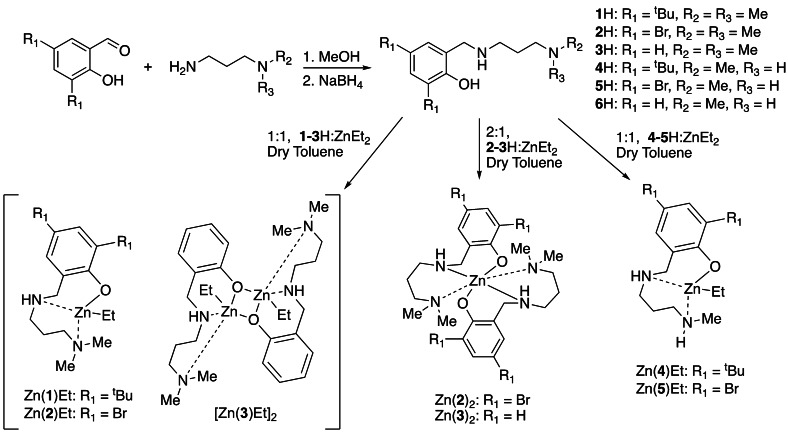
Preparation of half‐salan ligands and their corresponding homo‐ and heteroleptic Zn^II^‐complexes.

**Figure 1:**
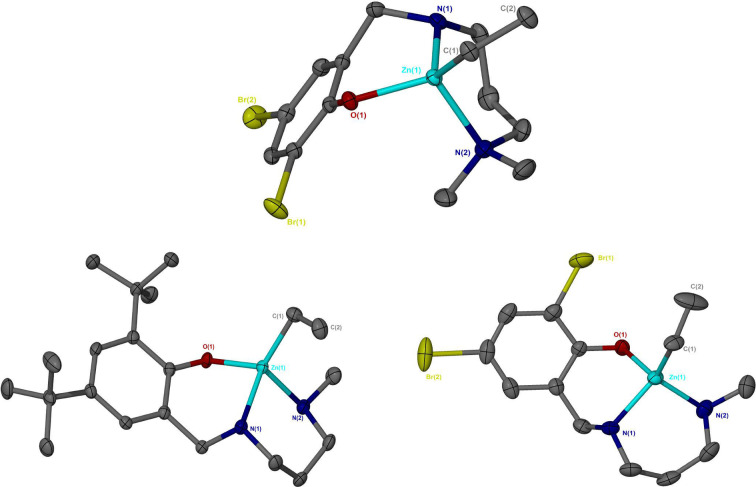
Solid‐state structures of Zn(**2**)Et (top centre), Zn(**4**)Et (bottom left) and Zn(**5**)Et (bottom right). Thermal ellipsoids shown at 50 % probability. All hydrogen atoms have been omitted for clarity.

**Figure 2:**
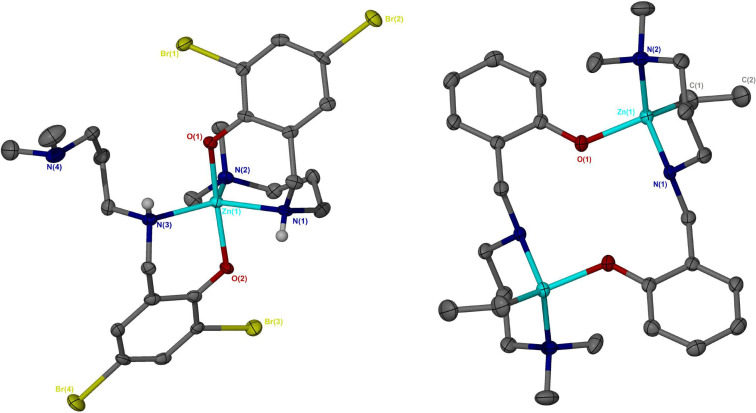
Solid‐state structures of Zn(**2**)_2_ (left) and [Zn(**3**)Et]_2_ (right). Thermal ellipsoids shown at 50 % probability. All hydrogen atoms, except those bound to N(1) and N(3) for Zn(**2**)_2_, have been omitted for clarity. [Zn(**3**)Et]_2_ has a centre of inversion to complete the macrocycle.

To assess polymerization activity all Zn^II^‐complexes were trialed in PLA production, specifically the ring‐opening polymerization (ROP) of lactide (Scheme S2). The racemic monomer (rac‐LA) was used to assess possible catalyst stereoselectivity. Industrially preferred melt conditions (130 and 180 °C) were used (Table S1), negating the need for an auxiliary solvent.^[11]^ In summary, Zn^II^‐complexes were generally highly active in the melt, achieving high conversion (78–94 %) within minutes under immortal conditions at 130 °C {[*rac*‐LA] : [M] : [BnOH]=10000 : 1 : 33} (Table S1). Catalyst colour and low metal loading (0.01 mol%) enabled the production of highly desirable white polymer, which was atactic (*P_r_* =0.54–0.59). Zn(**2**)_2_ was the outstanding candidate, achieving TOFs up to 273000 h^−1^ (Table S1, Entry 6), competitive with the industry standard; Sn(Oct)_2_.[^11^, ^12i^]

Superior *M_n_* control (*M*_*n,theo*_=13200 g mol ^−1^, *M_n_* =19350 g mol^−1^) and narrower dispersities (*Đ*=1.69) were realized upon modifying the [M] : [BnOH] ratio to 1 : 100, suggesting a more efficient initiation process (Table S1, Entry 6). Further polymerization and materials characterization data can be found in the Supporting Information.

**Figure 3:**
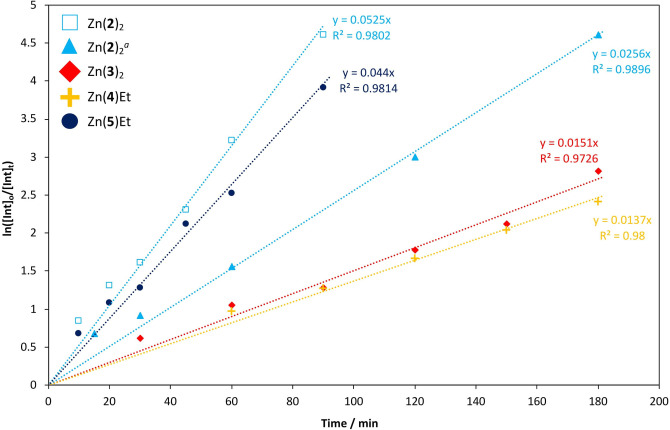
Pseudo‐first‐order logarithmic plot for the degradation of a PLLA (0.25 g, *M_n_* =45510 g mol^−1^) cup using Zn(**2‐3**)_2_ and Zn(**4‐5**)Et at 8 wt% (0.02 g, 0.72–1.4 mol% relative to ester linkages) in THF at 50 °C. Reaction conditions: *V*_THF_ : *V*_MeOH_=4 : 1, *n*_MeOH_ : *n*_ester_=7 : 1. [a] Zn(**2**)_2_=4 wt% (0.01 g, 0.36 mol% relative to ester linkages).

All Zn^II^‐complexes were also investigated in the metal‐mediated degradation of commercial PLA into methyl lactate (Me‐LA), a high value green solvent (Table S2).[^1c^, ^6a^] In short, Zn(**2‐3**)_2_ and Zn(**4‐5**)Et were taken forward for further kinetic analysis under mild reaction conditions (Figure 3 and Table S3). Reactivity trends could generally be attributed to ligand effects, with the presence of hydrogen bond donors and Lewis acidity of the Zn^II^ centre being particularly important.[^12e^, ^12g^, ^12i^] Notably, Zn(**2**)_2_ and Zn(**5**)Et were reasonably active at 8 wt%, exhibiting *k_app_* values of 0.053 and 0.044 min^−1^ respectively in THF at 50 °C (Table S3, Entries 2 and 5). Such values are comparable to previously reported Zn^II^‐complexes.[^12g^, ^12i^]

### Chemical Recycling of PET

Following PLA degradation success, our attention shifted to other commercial polyesters. Consequently, PET was selected, which accounted for *ca*. 23 % of plastic use within the packaging sector in 2015.^[3]^ Zn(**2**)_2_ was pursued due to its ease of preparation, stability and high activity. Unless otherwise stated, bottle‐grade PET (*M_n_* ∼40000 g mol^−1^) was used. All reaction times reflect the time taken to achieve complete PET dissolution, indicative of reaction completion. Degradation products were isolated as white solids *via* washing or recrystallization and characterized by ^1^H/^13^C{^1^H} NMR spectroscopy (Figures S59–S64).

### PET Glycolysis

**Scheme 2:**
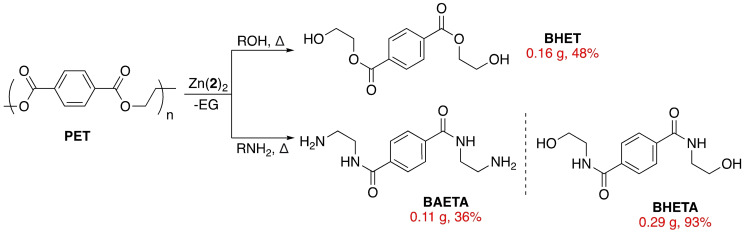
Metal‐mediated PET glycolysis and aminolysis using Zn(**2**)_2_.

Initial work focused on PET glycolysis, an established commercial process.^[13c]^ Degradation products include BHET and EG, constituting to monomer recovery (Scheme 2 and S4). Herein, a reaction temperature of 180 °C and an excess of EG (20.6 equiv.) in the presence of Zn(**2**)_2_ (4–8 wt%) were chosen based on previous work (Table S4).^[12g]^ Zn(OAc)_2_ ⋅ 2H_2_O is generally considered the benchmark catalyst, and thus was selected as a commercially available and air‐stable reference.^[1c]^ BHET was recovered *via* recrystallization from deionized H_2_O.

Zn(**2**)_2_ was reasonably active at 8 wt%, affording BHET in 48 % yield within 1.5 h (Table S4, Entry 6), competitive with previously reported discrete metal‐based complexes.[^12f^, ^12g^, ^15^] Interestingly, a PET thin‐film could be completely deconstructed within 1 h at 4 wt% (*Y_BHET_* =61 %; Table S4, Entry 4), highlighting activity dependence on sample morphology. For both sources of PET, Zn(**2**)_2_ exhibited higher activity relative to Zn(OAc)_2_ ⋅ 2H_2_O (Table S4, Entries 1 and 2). In all instances, BHET yields <65 % were observed, presumably due to the production of water‐soluble higher chain oligomers despite the use of a large excess of EG.[^1c^, ^13c^]

### PET Aminolysis

In pursuit of alternative value‐added chemicals from PET waste, our attention swiftly shifted to aminolysis for the production of terephthalamides (Scheme 2). Two commercially available aliphatic amines were chosen, namely ethanolamine and ethylenediamine.^[14]^ Lower reaction temperatures (110–120 °C) were used relative to glycolysis due to the aliphatic amines (6.4–16 equiv.) rendering the reaction more thermodynamically favourable (Table S5).[^1c^, ^14a^]

In the presence of Zn(**2**)_2_ at 8 wt%, terephthalamides were furnished in moderate to excellent yield (36–93 %) within 1–2 h (Table S5, Entries 2 and 4). Whilst BHETA results were comparable to previously reported systems, this work remains a rare example of discrete metal‐based aminolysis.[^1c^, ^12g^] BAETA was obtained in significantly lower yield (*Y_TPA_* =36 %) relative to BHETA (*Y_TPA_* =93 %), which could be attributed to product loss during arduous work‐up.

### Production of Poly(ester‐amide)s (PEA)

To achieve a completely circular upcycling approach, BHETA was selected for repolymerization owing to its ease of preparation. Recently, poly(ester‐amide)s (PEAs) have emerged as a promising hybrid material for both commodity and high‐performance applications, notably in the biomedical sector. Indeed, such materials could potentially be engineered to capture desirable qualities of both respective homopolymers (e. g. thermomechanical and possible biodegradability/recyclability).^[19]^ Thus, PEAs were targeted in proof‐of‐concept work.

**Figure 4:**
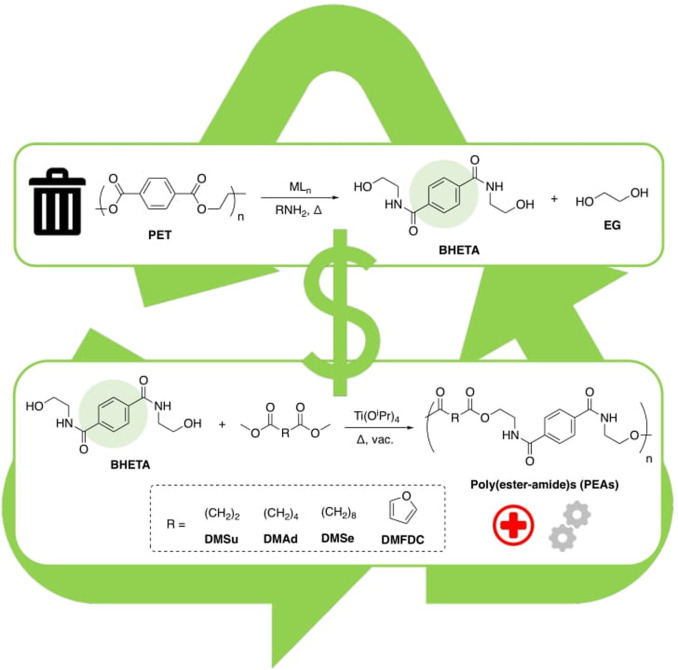
Melt polycondensation of BHETA with various diesters for PEA production.

Herein, we report PEA synthesis based on a range of aliphatic and aromatic diesters (Figure 4 and Table 1), employing transesterification by melt polycondensation as a model green polymerization process (see Supporting Information). Diesters were selected based on their ability to be renewably sourced, enabling such PEAs to be of 100 % recycled and bio‐based content.^[20]^ Moreover, PEA(**1‐4**) lend themselves to chemical recycling due to the presence of cleavable ester linkages. Titanium alkoxides are well‐known ROP and polycondensation catalysts, thus Ti(O^i^Pr)_4_ was used.^[21]^

**Table 1:** Polycondensation of diesters with BHETA for PEA production.

Diester	PEA	Mn^[a]^ [g mol^−1^]	Đ^[a]^	Tg^[b]^ [°C]	Td‐5^[c]^ [°C]	Td‐50^[c]^ [°C]
DMSu	PEA(**1**)	1750	3.36	118	296	392
DMAd	PEA(**2**)	2150	3.72	114	293	397
DMSe	PEA(**3**)	3150	2.91	98	293	388
DMFDC	PEA(**4**)	2450	3.24	126	275	345

Reaction conditions: BHETA (0.23 mg, 0.92 mmol), diester (0.13–0.21 g, 0.92 mmol) and Ti(O^i^Pr)_4_ (400 ppm, stock solution, 20 μL mL^−1^ in toluene). Polycondensation step conducted at 210 °C for 3 h under a dynamic vacuum (<1 mbar). [a] Determined *via* SEC analysis in *N,N‐*DMAc with 0.1 % w/v LiBr. [b] Determined by DSC analysis. [c] *T*_*d‐5*_ and *T*_*d‐50*_ values refer to 5 % and 50 % weight loss respectively during TGA analysis under an argon atmosphere.

^1^H NMR spectroscopic analysis (Figures S65–S68) confirmed PEA production. Notably, we report the synthesis of PEA(**3**) and PEA(**4**) for the first time. Indeed, PEA(**4**) represents new opportunities for bio‐based aromatic polymers, which have previously been hindered by limited monomer scope and challenging synthesis.^[20]^ SEC analysis revealed PEA(**1‐4**) to be of moderate *M_n_* (1750–3150 g mol^−1^) and highly disperse (*Đ*=2.91–3.72) (Table 1). These *M_n_* values imply a loss in stoichiometric balance, evidenced by the presence of residual BHETA in PEA(**1‐3**) (Figures S65–S67). This is possibly due to a number of factors including trace monomer impurities, side reactions and mass transfer limitations.^[22]^ However, such results are amenable to further optimization. Demarteau et al.^[14b]^ have previously reported PEA(**1–2**), achieving comparable *M_n_* with narrower dispersities (*Đ*=1.34–1.46). However, DSC analysis (Figure S70) revealed PEA(**1‐2**) to possess significantly higher *T_g_* values of 118 and 114 °C (relative to 7 and 18 °C respectively), evidencing greater hydrogen bonding interactions. For PEA(**1‐3**) an increase in diester chain length coincided with a decrease in *T_g_*, as expected, although PEA(**3**) retained an impressive *T_g_* of 98 °C (Table 1, Entry 3). Substitution of the flexible DMSe linker for DMFDC yielded the highest *T_g_* of 126 °C (Table 1, Entry 4), owing to increased polymer rigidity. TGA analysis (Figure S71) was used to determine *T*_*d‐5*_ and *T*_*d‐50*_ values, which highlight good polymer thermal stability and robustness (Table 1). Such thermal properties are truly exceptional compared to conventional PEAs and other commercial plastics including PET and PEF.[^3^, ^19^] We anticipate this discovery to inspire new and exciting developments in both PET waste management and PEA design, particularly for bio‐based aromatic polymers.

### Chemical Recycling of BPA‐PC

We then sought to explore Zn(**2**)_2_ for the recycling of other polymer classes, identifying polycarbonates (PC) as a key area of opportunity for metal‐based and sustainable solutions. Presently, PC production equates to *ca*. 5 million tonnes p/a, with BPA‐PC being the market dominant polymer.^[13a]^

### BPA‐PC Methanolysis

**Scheme 3:**
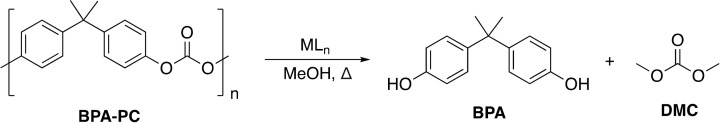
Metal‐mediated BPA‐PC methanolysis into BPA and DMC.

Preliminary experiments were conducted at 75 °C based on previous state‐of‐the‐art organocatalytic systems.^[16]^ Commercial BPA‐PC pellets (*M_w_* ∼45000 g mol^−1^) and 4 wt% Zn(**2**)_2_ (1.3 mol%) were used. 2‐Me‐THF (solvent) and an initial *n*_carbonate_ : *n*_MeOH_ ratio of 1 : 25 were chosen based on previous work in our group.^[16h]^ Reaction progress was monitored using ^1^H NMR (Figures S72–S75), observing BPA and DMC as the main degradation products (Scheme 3). Unless otherwise stated, complete BPA‐PC consumption was observed. *S_BPA_* and *S_OC_* refer to BPA and organic carbonate selectivity respectively.

**Figure 5:**
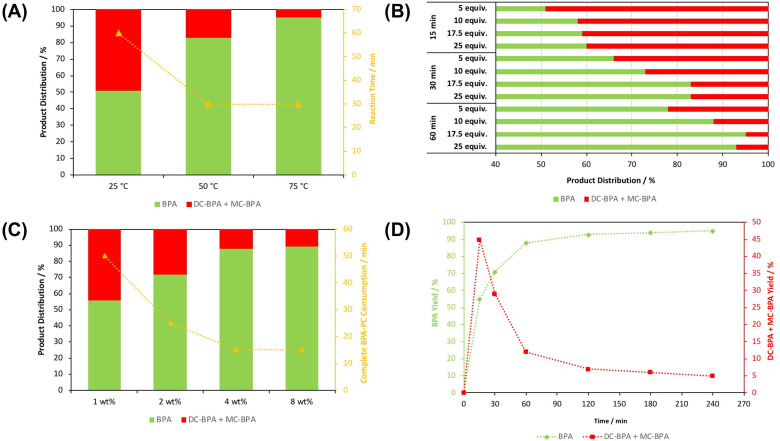
Effect of different reaction parameters on BPA‐PC methanolysis: reaction temperature {*n*_carbonate_ : *n*_MeOH_=1 : 25} (**A**), *n*_carbonate_ : *n*_MeOH_ (**B**), catalyst loading {Zn(**2**)_2_=1–8 wt%=0.0025–0.02 g=0.32–2.5 mol% (relative to carbonate linkages), *n*_carbonate_ : *n*_MeOH_=1 : 10, 1 h} (**C**) and reaction time {*n*_carbonate_ : *n*_MeOH_=1 : 10} (**D**). Reaction conditions: BPA‐PC (0.25 g, *M_w_* ∼45000 g mol^−1^), solvent: 2‐methyltetrahydrofuran (2‐Me‐THF, 4 mL), Zn(**2**)_2_=4 wt% (0.01 g, 1.3 mol% relative to carbonate linkages) and 50 °C.

Promisingly, Zn(**2**)_2_ was shown to facilitate rapid BPA‐PC methanolysis at 75 °C (Figure 5a). Thus, the effect of various reaction parameters on activity was explored (Figure 5).

### Effect of Reaction Parameters

Between 25–75 °C (Figure 5a), an increase in temperature coincided with enhanced BPA yield (up to 95 %) and a reduction in BPA‐PC consumption rate (5–20 min). Beyond Arrhenius behaviour, it is proposed higher reaction temperatures favour BPA‐PC dissolution and swelling.[^16c^, ^17a^] Prior to BPA formation, mono‐ (MC‐BPA) and di‐carbonate BPA (DC‐BPA) were formed, which were considerably more pronounced at 25 °C despite a prolonged reaction time of 1 h. To assess the need for an auxiliary solvent, reactions between 50–75 °C were repeated in DMC (Table S6, Entries 1 and 2). Unsurprisingly, solvent exchange exasperated the production of MC‐BPA and DC‐BPA, resulting in a lower yield of BPA (*S_BPA_* =68–80 %).^[16h]^ Consequently, 2‐Me‐THF was retained, citing its green credentials.^[23]^ A reaction temperature of 50 °C was chosen in the interest of potential cost‐energy savings.

To reduce process waste, the influence of *n*_carbonate_ *:n*_MeOH_ on activity was investigated with time (Figure 5b). Reaction progress was characterized by the consumption of MC‐BPA and DC‐BPA, resulting in superior BPA yield. Good process efficiency was maintained between 1 : 25 to 1 : 10 (*n*_carbonate_ : *n*_MeOH_), achieving between 88–95 % BPA yield within 1 h. Further decreasing the *n*_carbonate_ : *n*_MeOH_ molar ratio to 1 : 5 resulted in a significant drop in productivity (78 % BPA yield), which could be attributed to dilution effects. The rate of BPA‐PC consumption (15 min) appeared independent of *n*_carbonate_ : *n*_MeOH_, implying product distribution is equilibrium limited. A *n*_carbonate_ : *n*_MeOH_ ratio of 1 : 10 was optimal for this reaction.

Catalyst loading was shown to impact both BPA yield and the rate of BPA‐PC consumption (Figure 5c). Between 1–4 wt% the yield of BPA gradually improved from 56 to 88 %, whilst the rate of BPA‐PC consumption decreased from 50 to 15 min. No appreciable impact on process efficiency was observed between 4–8 wt%. Indeed, catalyst saturation has previously been shown to adversely impact productivity by catalysing detrimental side reactions.^[17c]^ Thus, optimal BPA recovery was possible at 4 wt% (1.3 mol%).

The effect of reaction time on BPA yield was also monitored (Figure 5d). The yield of BPA increased rapidly to 88 % within the first hour before plateauing, consistent with the reaction intermediate consumption profile and previous reports.[^16c^, ^17^] Whilst a slight increase in BPA yield (up to 95 %) was observed with prolonged stirring (4 h), 1 h was deemed sufficient to avoid a protracted reaction time.

Such optimized conditions are promising compared to previously reported systems, in particular the use of low temperatures and loadings.[^13a^, ^16^, ^17^]

### Reaction Scope

**Figure 6:**
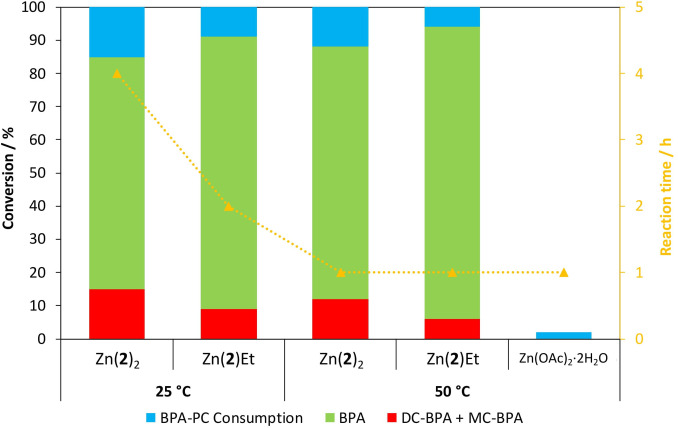
Catalyst performance comparison for BPA‐PC methanolysis. Reaction conditions: BPA‐PC (0.25 g, *M_w_* ∼45000 g mol^−1^), *n*_carbonate_ : *n*_MeOH_=1 : 10, Zn^II^‐complexes=4 wt% (0.01 g, 1.3–2.2 mol% relative to carbonate linkages), Zn(OAc)_2_ ⋅ 2H_2_O=4 wt% (0.01 g, 4.4 mol% relative to carbonate linkages), solvent: 2‐Me‐THF (4 mL).

Under the optimized conditions {*n*_carbonate_ : *n*_MeOH_=1 : 10, 4 wt% catalyst, 1 h at 50 °C}, reaction scope was further probed (Table S6). Surprisingly, Zn(**2**)Et maintained excellent performance relative to Zn(**2**)_2_ under analogous conditions (Figure 6). Zn(OAc)_2_ ⋅ 2H_2_O was virtually inactive at 50 °C, highlighting the need for ligated complexes. Impressive Zn(**2**)_2_ and Zn(**2**)Et tolerance was demonstrated through the use of mixed feeds and the degradation of a compact disc (CD), which represented a challenging BPA‐PC composite (Table S6, Entries 3 and 7–9). Solvent‐free methanolysis using 4 wt% Zn(**2**)Et at 50 °C was unsuccessful, which could be attributed to poor polymer solubility in MeOH (Table S6, Entry 10).^[13a]^ Organic carbonate substrate scope was extended to diethyl carbonate (DEC) *via* ethanolysis using Zn(**2**)Et, further promoting a green approach since ethanol can be renewably derived (Table S6, Entry 6 and Figure S77).^[24]^ However, an extended reaction time of 2 h was conceded to achieve comparable BPA yield (*S_BPA_* =88 %), owing to ethanol being a poorer nucleophile. In all instances, organic carbonate yield was *ca*. 10 % lower relative to BPA values (Table S6). Indeed, the presence of residual H_2_O has previously been shown to inhibit DMC formation, consistent with the use of wet solvents.^[17c]^

More importantly, we report the first example of discrete metal‐mediated BPA‐PC methanolysis being appreciably active at RT. Indeed, high BPA yields (*S_BPA_* =85–91 %) were obtainable within 2–4 h (Figure 6), surpassing previous state‐of‐the‐art organocatalysts, such as TBD and DBU, which required prolonged stirring at RT (12–160 h).[^16a^, ^16b^] Zn(**2**)Et furnished BPA in notably higher yields in reduced time relative to Zn(**2**)_2_. This can likely be attributed to Zn(**2**)_2_ being present in a slightly lower molar loading of 1.3 mol% (relative to 2.2 mol%) at 4 wt%, which could be compensated for by an increase in temperature to 50 °C (Figure 6). An acid control using dilute HCl (1 mol%) revealed no BPA‐PC consumption after 4 h at RT, further highlighting the promise of these catalysts (Table S6, Entry 14). For Zn(**2**)_2_, increasing the *n*_carbonate_ *:n*_MeOH_ molar ratio to 1 : 25 had no effect on activity, as expected (Table S6, Entry 4). An ambient and selective recycling strategy using a mixed BPA‐PC:PET feed in the presence of Zn(**2**)Et at 4 wt% was demonstrated on a multigram scale (Table S6, Entry 12). Crucially, process efficiency was maintained, whilst ^1^H NMR and isolated BPA yields (88–89 %) were comparable (Figure S76).

### Degradation Kinetics

**Figure 7:**
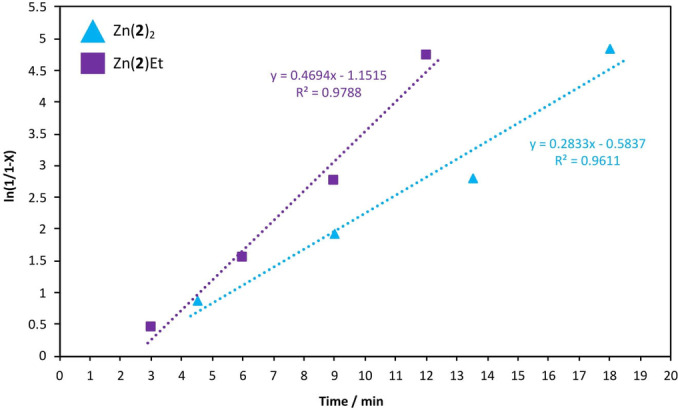
Pseudo‐first‐order logarithmic plot for BPC‐PC (0.25 g, *M_w_* ∼45000 g mol^−1^) methanolysis using Zn(**2**)_2_ and Zn(**2**)Et at 4 wt% (0.01 g, 1.3–2.2 mol% relative to carbonate linkages) at room temperature. Reaction conditions: *n*_carbonate_ : *n*_MeOH_=1 : 10, solvent: 2‐Me‐THF (4 mL).

Whilst initial work established a product distribution dependence on temperature (Figure 5a and Figure 6), comparable BPA‐PC consumption rates were observed between 25–50 °C (*ca*. 15 min). Thus, kinetic analysis was pursued at RT (Figure 7 and Table S7). A plot of ln(1/1‐X) against time, where X refers to BPC‐PC conversion by weight, exhibited a linear relationship. This suggests the reaction proceeds pseudo‐first‐order with respect to BPA‐PC consumption, consistent with previous work by Song and co‐workers.^[16i]^ Consequently, the gradient of the logarithmic plot is equivalent to the apparent rate constant, *k_app_*.

Remarkably, Zn(**2**)_2_ and Zn(**2**)Et exhibited *k_app_* values of 0.28 and 0.47 min^−1^ respectively at 4 wt% (Table S7, Entries 1 and 2), the highest reported to date. This breakthrough provides the foundation for further metal‐mediated innovation, opening new possibilities for polycarbonate recycling under mild conditions.

### Catalyst Stability

Stability testing in excess MeOH at RT was conducted to further probe the active species. ^1^H NMR analysis revealed Zn(**2**)_2_ to be stable (Figure S47). However, a change in coordination number around the metal center during methanolysis cannot be excluded due to lability of the pendant amine.^[12i]^ Conversely, Zn(**2**)Et reacted to generate the corresponding ‐OMe analogue (Zn(**2**)OMe) *via* elimination of the terminal ‐Et to afford dissolve ethane (Figure S48). DOSY analysis of Zn(**2**)OMe revealed one species exists exclusively in solution with a diffusion constant (*D*) of 0.71×10^−9^ m^2^ s^−1^ (Figure S49). Despite having comparable *M_r_* values, this was lower relative to Zn(**2**)Et (*D*=0.90×10^−9^ m^2^ s^−1^; Figure S30), possibly evidencing the formation of a dimer in solution.

### BPA‐PC Glycolysis

Recent work has highlighted the potential to access valuable cyclic carbonate monomers *via* BPA‐PC glycolysis.[^16f^, ^16g^] Traditionally, such chemicals are derived from toxic phosgene or CO, creating an appetite for greener alternatives.^[25]^ Consequently, we sought to diversify product scope. Reaction progress was monitored using ^1^H NMR analysis (Figures S78–S80), employing tetramethylsilane (TMS) as an internal standard to determine product yields (Table S8). Zn(**2**)Et was selected due to catalyst availability.

**Scheme 4:**
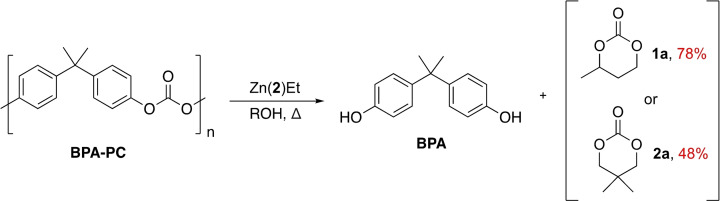
Metal‐mediated BPA‐PC glycolysis using Zn(**2**)Et where ROH refers to *rac‐*butane‐1,3‐diol (**1 a**) and 2,2‐dimethyl‐1,3‐propanediol (**2 a**) respectively.

Promisingly, high value cyclic carbonates (Scheme 4) were obtained in moderate to good yield (48–78 %; Table S8, Entries 1 and 4), demonstrating catalyst versatility.^[9e]^ Cyclic carbonate selectivity (*S_CC_*) was shown to be dependent on both reaction time and *n*_glycol_ : *n*_carbonate_ (Table S8). Lower reaction temperatures (75 °C), catalyst loadings (2.2 mol%) and reduced reaction times (1 h) were used relative to an organocatalyst (TBD : MSA) reported by Jehanno et al.,^[16f]^ highlighting the promise of metal‐mediated solutions for such green transformations. Additionally, excellent BPA recovery (*S_BPA_* =96–99 %) was observed under all reaction conditions (Table S8).

### Chemical Recycling of Poly(propylene carbonate)

Finally, we endeavoured to extend Zn(**2**)_2_ and Zn(**2**)Et to other commercial polycarbonates, such as poly(propylene carbonate) (PPC). PPC is a copolymer based on propylene oxide (PO) and CO_2_ with uses in packaging and biomedical materials, among others.^[26]^ Reductive depolymerization strategies for PPC have been widely reported, although typically rely on precious metals and forcing reaction conditions.^[10c]^

To the best of our knowledge, we report the first example of PPC methanolysis (Scheme S11), producing propylene carbonate (PC) in moderate to good yield (up to 58 % in 1.5 h) at 50 °C (Table S9, Entry 2). PC is both a valuable green solvent and intermediate for the production of DMC *via* transesterification.^[18c]^ ^1^H NMR analysis revealed the production of unidentified side‐products, likely due to competing product equilibria (Figure S81), which appeared dependent on both *n*_carbonate_ : *n*_MeOH_ and reaction time (Table S9). In the absence of catalyst, no PPC consumption was observed (Table S9, Entry 1). This was monitored *via* ^1^H NMR, necessitating the use of THF to circumvent solvent/polymer peak overlapping (Figure S82).

## Conclusion

A range of Zn^II^‐complexes based on half‐salan ligands have been prepared and fully characterized in both the solution and solid‐state. Catalyst versatility and robustness was demonstrated through the mild and selective degradation of various commercial polyesters and polycarbonates. A range of strategies (e. g. alcoholysis, glycolysis and aminolysis) were employed to obtain a diverse range of value‐added chemicals with uses as green solvents and chemical building blocks. Remarkably, Zn(**2**)_2_ and Zn(**2**)Et were shown to facilitate rapid BPA‐PC methanolysis (12–18 min) at room temperature in 2‐Me‐THF under low loadings. Further kinetic analysis found such catalysts to possess *k_app_* values of 0.28±0.040 and 0.47±0.049 min^−1^ respectively at 4 wt%, the highest reported to date. Product distribution was found to be dependent on a number of parameters including temperature, *n*_carbonate_ : *n*_MeOH_, catalyst loading and reaction time. Proof‐of‐concept work demonstrated a completely circular upcycling approach to plastic waste through the production of several renewable PEAs based on a terephthalamide monomer derived from bottle‐grade poly(ethylene terephthalate) (PET). These materials displayed excellent thermal properties, observing *T_g_* values up to 126 °C, whilst noting scope for further optimization. Such findings promise to stimulate new and exciting developments in both polymer design and recycling, enabling key challenges associated with a dynamic and evolving plastics economy to be addressed. Further work is ongoing to explore the optimization of such catalysts for chemical recycling applications.

## Experimental Section

Exemplar procedures are provided below, see Supporting Information for full details.

### Materials

The synthesis and characterization of all metal complexes was performed under an inert atmosphere of argon using standard Schlenk or Glovebox techniques. All chemicals used were purchased from Sigma‐Aldrich and used as received, with the exception of rac‐lactide (*rac‐*LA), which was recrystallized once from anhydrous toluene prior to use. Dimethyl succinate (98 %+, Alfa Aesar), dimethyl adipate (99 %, Acros Organics), dimethyl sebacate (97 %, Alfa Aesar) and dimethyl 2,5‐furandicarboxylate (99 %, Sarchem laboratories) were sourced from alternative suppliers. Commercial poly(lactic acid) (PLA) samples were purchased (PLLA cup, *M_n_* =45,510 g mol^−1^, *Vegware* ^*TM*^; R600Y‐VW) and cut up into 0.1×0.1 cm^2^ pieces before degradation. PLLA‐based 3D printing material was kindly provided by Filamentive. Bottle‐grade poly(ethylene terephthalate) (PET) (Coke Bottle, *The Coca‐Cola Company* ^*TM*^, *M_n_* ∼40,000 g mol^−1^) was sourced from a local grocery store (Fresh, University of Bath), rinsed with acetone, air‐dried and cut up into 0.1×0.1 cm^2^ pieces before degradation. PET thin‐films represent waste from the manufacturing sector and were kindly donated by Avery Dennison. Poly(bisphenol A carbonate) (BPA‐PC) (*M_w_* ∼45,000 g mol^−1^) and poly(propylene carbonate) (*M_n_* ∼50,000 g mol^−1^) pellets were sourced from Sigma‐Aldrich and used as received. All dry solvents used in handling all metal complexes were obtained *via* SPS (solvent purification system).

### Ligand

**2**H: To a solution of 3,5‐dibromosalicylaldehyde (0.56 g, 2 mmol) in MeOH (5 mL), 3‐(dimethylamino)‐1‐propylamine (0.25 mL, 2 mmol) was added dropwise and the resulting solution was stirred for 3 h at room temperature. NaBH_4_ (0.19 g, 5 mmol, 2.5 equiv.) was added portion‐wise with stirring, observing rapid precipitation of a white powder in a colourless solution, which was stirred for 1 h at room temperature. The reaction mixture was quenched with deionized H_2_O (5 mL) and the solvent concentrated *in vacuo*. A white powder was isolated by Buchner filtration, washed with deionized H_2_O (4×5 mL) and dried *in vacuo*.

### Zn^II^‐Complex

Zn(**2**)_2_: To a solution of **2**H (0.55 g, 1.5 mmol) in dry toluene (7.5 mL), ZnEt_2_ (0.75 mL, 0.75 mmol, 1.0 m in hexane) was added dropwise with stirring, observing rapid precipitation of a white powder. The resulting suspension was stirred at 80 °C overnight under a static argon atmosphere. The product was redissolved upon vigorous heating with stirring and the flask transferred to a freezer. After 3 days at −18 °C, a white solid powder was isolated by cannula filtration and dried *in vacuo* at 80 °C for 4 h.

### BPA‐PC Degradation

General procedure: A J Young's ampoule was charged with BPA‐PC pellets (0.25 g, *M_w_* ∼45,000 g mol^−1^) and metal complex (1–8 wt %, 0.0025–0.02 g, 0.32–2.5 mol% relative to carbonate linkages) in a glovebox filled with argon. 2‐Me‐THF (4 mL) and a desired amount of MeOH (0.21–1 mL, 5–25 equiv.) were added under a dynamic flow of argon and the ampoule was submerged in a pre‐heated oil bath (50 °C), observing complete polymer dissolution within 15 to 30 min. Sample aliquots were taken under a flow of argon and analysed by ^1^H NMR (CDCl_3_) spectroscopy.

## Conflict of interest

The authors declare no conflict of interest.

1

## Supporting information

As a service to our authors and readers, this journal provides supporting information supplied by the authors. Such materials are peer reviewed and may be re‐organized for online delivery, but are not copy‐edited or typeset. Technical support issues arising from supporting information (other than missing files) should be addressed to the authors.

Supporting Information

## Data Availability

The data that support the findings of this study are available in the supplementary material of this article.
