# Supplementary material for: Versatile Chemical Recycling Strategies: Value‐Added Chemicals from Polyester and Polycarbonate Waste
Source: ChemSusChem. 2022 Feb 23;15(8):e202200255. doi: 10.1002/cssc.202200255 (PMC9306953; doi:10.1002/cssc.202200255)
Supplement: Supplementary file 1 — Supporting Information [file CSSC-15-0-s001.pdf]

ChemSusChem

Supporting Information

Versatile Chemical Recycling Strategies: Value-Added Chemicals from Polyester and Polycarbonate Waste

Jack M. Payne, Muhammad Kamran, Matthew G. Davidson, and Matthew D. Jones*© 2022 The Authors. ChemSusChem published by Wiley-VCH GmbH. This is an open access article under the terms of the Creative Commons Attribution License, which permits use, distribution and reproduction in any medium, provided the original work is properly cited.

Contents

1. General Experimental Methods 3-4
2. Synthesis and Characterization 5-47
    2.1. Half-Salan Ligands 5-22
    2.2. Zn(II)-Complexes 23-43
    2.3. Catalyst Stability Testing 44-47
3. Lactide Polymerization 48-52
    3.1. Polymerization of *rac*-LA 48
        3.1.1. Representative ^{1}H NMR Spectrum 49
        3.1.2. Representative SEC Spectra 50
        3.1.3. Representative Homonuclear Decoupled ^{1}H NMR Spectrum 51
        3.1.4. MALDI-ToF Spectra 52
4. PLA Degradation 53-56
    4.1. PLA Methanolysis 53
        4.1.1. Degradation Kinetics 54
        4.1.2. Representative ^{1}H NMR Spectra 54-56
5. PET Degradation 57-67
    5.1. PET Glycolysis 57
        5.1.1. Representative ^{1}H NMR Spectrum 58
    5.2. PET Aminolysis 59
        5.2.1. *N,N'*-bis(2-aminoethyl)terephthalamide (BAETA) 59-61
        5.2.2. *N,N'*-bis(2-hydroxyethyl)terephthalamide (BHETA) 61-62
    5.3. Renewable Poly(ester-amide)s 63
        5.3.1. Melt Polycondensation Method 63
        5.3.2. Poly(ester-amide) Characterization 64
            5.3.2.1. ^{1}H NMR spectra 64-65
            5.3.2.2. Representative SEC spectrum 66
            5.3.2.3. Thermal Analysis 66-67
6. Polycarbonate Degradation 68-79
    6.1. BPA-PC Methanolysis 68
        6.1.1. Degradation Kinetics 69
        6.1.2. Representative ^{1}H NMR Spectra 69-72
    6.2. BPA-PC Glycolysis 73
        6.2.1. 4-Methyl-1,3-dioxan-2-one 74
        6.2.2 5,5-Dimethyl-1,3-dioxan-2-one 75-76
    6.3. PPC Methanolysis 77
        6.3.1. Representative ^{1}H NMR Spectra 78-79
7. Crystallographic Data 80

References 81

1. General Experimental Methods

The synthesis and characterization of all metal complexes was performed under an inert atmosphere of argon using standard Schlenk or Glovebox techniques. All chemicals used were purchased from Sigma-Aldrich and used as received, with the exception of *rac*-lactide (*rac*-LA), which was recrystallized once from anhydrous toluene prior to use. Dimethyl succinate (98%+, Alfa Aesar), dimethyl adipate (99%, Acros Organics), dimethyl sebacate (97%, Alfa Alfa Aesar) and dimethyl 2,5-furandicarboxylate (99%, Sarchem laboratories) were sourced from alternative suppliers. Commercial poly(lactic acid) (PLA) samples were purchased (PLLA cup, M_n = 45,510 g mol^{-1}, *Vegware*^{TM}; R600Y-VW) and cut up into 0.1 × 0.1 cm^2 pieces before degradation. PLLA-based 3D printing material was kindly provided by Filamentive. Bottle-grade poly(ethylene terephthalate) (PET) (Coke Bottle, *The Coca-Cola Company*^{TM}, M_n ~ 40,000 g mol^{-1}) was sourced from a local grocery store (Fresh, University of Bath), rinsed with acetone, air-dried and cut up into 0.1 × 0.1 cm^2 pieces before degradation. PET thin-films represent waste from the manufacturing sector and were kindly donated by Avery Dennison. Poly(bisphenol A carbonate) (BPA-PC) (M_w ~ 45,000 g mol^{-1}) and poly(propylene carbonate) (M_n ~ 50,000 g mol^{-1}) pellets were sourced from Sigma-Aldrich and used as received. All dry solvents used in handling all metal complexes were obtained *via* SPS (solvent purification system).

^{1}H and ^{13}C{^{1}H} NMR spectra were obtained on either a Bruker 400 or 500 MHz spectrometer and referenced to residual solvent resonances.[1] HSQC and VT NMR experiments were run on a Bruker 500 MHz spectrometer. Coupling constants (*J*) are provided in Hertz (Hz) to the nearest integer. $CDCl_3$ was dried over CaH_2 prior to use with all metal complexes. C_6D_6 was degassed and stored over molecular sieves for use with all metal complexes. All ligands were prepared *via* novel synthetic procedures and characterized *via* electron-spray ionisation-mass spectrometry (ESI-MS) in positive mode. CHN microanalysis was performed by Elemental Microanalysis under an inert atmosphere. Diffusional ordered spectroscopy (DOSY) NMR analysis was carried out on a Bruker 500 MHz instrument.[2] The standard Bruker pulse sequence ledsp2s used was with 8 scans recorded per gradient level. A gradient strength between 1600 to 1750 μs was used with a diffusion time of 0.05 seconds. Eight gradient strengths were used between 10 to 90 %. Data was processed using DOSY methods.

Single crystal X-ray data was collected on an EOS SuperNova diffractometer with Cu Kα radiation (λ = 1.54184 Å) at 150(2) K unless otherwise stated. All structures were solved by direct methods and refined on all F^2 data using the SHELXL-2014 or 2017 suite of programs. All hydrogen atoms were included in idealized positions and refined using the riding model, all refinement details are given in the .cif file.

All polymer molecular weights (M_n) and dispersities (*Đ*) were characterized by size exclusion chromatography (SEC), which was performed on an Agilent 1260 infinity instrument using a flow rate of 1 mL min^{-1} at 35 °C with a THF eluent and a PLgel 5 μm MIXED-D 300 × 7.5 mm column. The system was referenced against 11 narrow molecular weight polystyrene standards with detection *via* refractive index response. Poly(ester-amide)s derived from PET were found to be insoluble in THF, thus *N,N*-dimethylacetamide (*N,N*-DMAc) was used as an alternative eluent. Subsequent analysis was performed on an Agilent 1260 infinity instrument using a flow rate of 1 mL min^{-1} at 50 °C with a *N,N*-DMAc and 0.1% w/v LiBr eluent and a Polargel-M 300 × 7.5 mm column. The system was referenced against 11 narrow molecular weight polymethylmethacrylate (PMMA) standards with detection *via* refractive index response. Polymer (PLA) tacticity was determined *via* homonuclear decoupled ^{1}H NMR

($CDCl_3$) spectroscopic analysis of the methine region in accordance to relationships described by Coates *et al.*[3] MALDI-ToF mass spectra were determined on a Bruker Autoflex speed instrument using DCTB (*trans*-2-[3-(4-tert-Butylphenyl)-2-methyl-2-propenylidene]malononitrile, 10 μL, 10 mg mL^{-1} in THF) as the matrix and ionized using NaTFA (2 μL, 0.1 M in THF) for PLA (10 μL, 5 mg mL^{-1} in THF). After centrifugation, homogeneous samples of PLA (2 μL) were spotted onto a polished steel plate and data was collected in linear positive mode.

Differential scanning calorimetry (DSC) analysis was conducted on a TA-instrument Q20 differential scanning calorimeter. All runs were performed under a nitrogen atmosphere with a constant flow rate of 50 mL min^{-1}. The sample (4 – 6 mg) was first equilibrated at -10 °C and then heated to 225 - 250 °C at a heating rate of 20 °C min^{-1} to construct the first heating curve. The sample was held isothermally for 1 min before starting the cooling scan. In the cooling scan, the sample was cooled at a rate of 10 °C min^{-1} down to -10 °C, which was maintained for 1 minute. Finally, in the second heating scan, the temperature was increased from -10 to 225 - 250 °C using a heating rate of 10 °C min^{-1}. The glass transition temperature (T_g) was measured on the second heating scan.

Thermogravimetric analysis (TGA) was performed on a Setsys Evolution TGA 16/18 from Setaram. The sample (8 – 12 mg) was loaded into a 170 μL alumina crucible and the furnace purged with argon for at least 30 minutes at room temperature. The sample was then heated from room temperature to 120 °C at a heating rate of 10 °C min^{-1}, which was maintained for 20 minutes to remove any residual water. The sample was then further heated to 700 °C at a heating rate of 10 °C min^{-1}, all under a constant flow of argon.

Materials characterization (SEC, ESI-MS and MALDI-ToF) facilities were provided by the Material and Chemical Characterization Facility (MC^2) at the University of Bath.

2. Synthesis and Characterization

2.1. Half-Salan Ligands

1H:

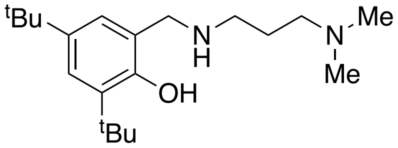

To a solution of 3,5-di-*tert*-butyl-2-hydroxybenzaldehyde (0.47 g, 2 mmol) in MeOH (5 mL), 3-(dimethylamino)-1-propylamine (0.25 mL, 2 mmol) was added dropwise and the resulting solution was stirred for 3 h at room temperature. $NaBH_4$ (0.38 g, 10 mmol, 5 equivalents) was added portion-wise and the solution was stirred overnight, observing discolouration to afford a colourless solution. The reaction mixture was quenched with deionized H_2O (5 mL) and the solvent removed *in vacuo*. The resulting oil was redissolved in DCM (30 mL), washed with deionized H_2O (3 × 30 mL) and the solvent removed *in vacuo*. The product was dried *in vacuo* for 1 h and collected as a white oil.

^1H NMR (C_6D_6, 400 MHz): δ = 7.51 (d, *J* = 2 Hz, 1H; ArH), 6.93 (d, *J* = 2 Hz, 1H; ArH), 3.56 (s, 2H; Ar-CH_2), 2.30 (t, *J* = 7 Hz, 2H; CH_2), 1.98 (t, *J* = 7 Hz, 2H; CH_2), 1.95 (s, 6H; $N(CH_3)_2$), 1.74 (s, 9H; $C(CH_3)_3$), 1.39 (s, 9H; $C(CH_3)_3$), 1.27 (p, *J* = 7 Hz, 2H; CH_2). *N.B.* Unaccounted for -OH and -NH resonances, indicative of rapid proton exchange in solution. HSQC analysis assisted with peak assignment.

$^{13}C\{^1H\}$ NMR (C_6D_6, 125 MHz): δ = 155.8, 140.4, 136.2, 123.5, 122.9, 122.8 (Ar), 58.2 (CH_2), 54.0 (Ar-CH_2), 47.5 (CH_2), 45.5 ($N(CH_3)_2$), 35.4, 34.4 ($C(CH_3)_3$), 32.1, 30.1 (CH_3), 27.3 (CH_2). *N.B.* HSQC analysis assisted with peak assignment.

Yield = 0.39 g, 61%

ESI-MS (+ve, MeOH): Calculated m/z $[C_{20}H_{37}N_2O]^+$ = 321.2906; found m/z = 321.2946.

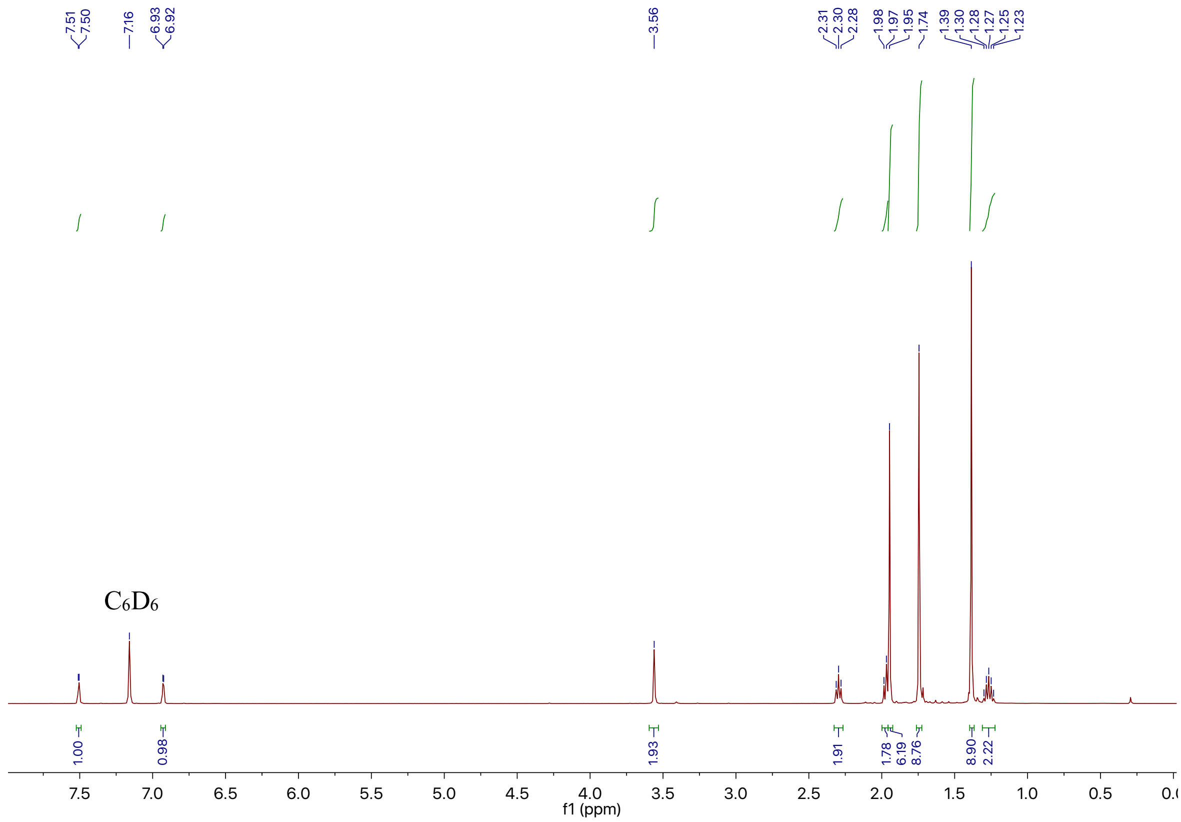

Figure S1. ^{1}H NMR (C_6D_6, 400 MHz) spectrum of **1**H.

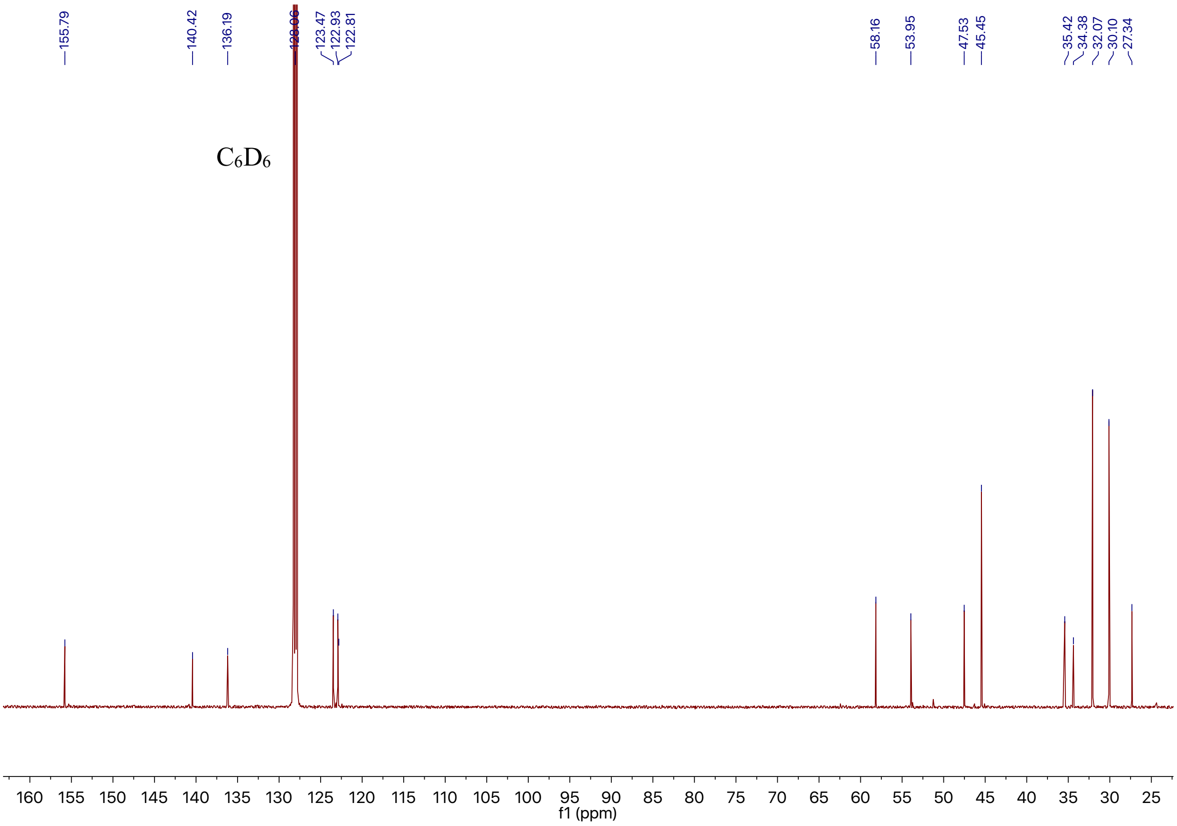

Figure S2. $^{13}C\{^{1}H\}$ NMR (C_6D_6, 125 MHz) spectrum of **1**H.

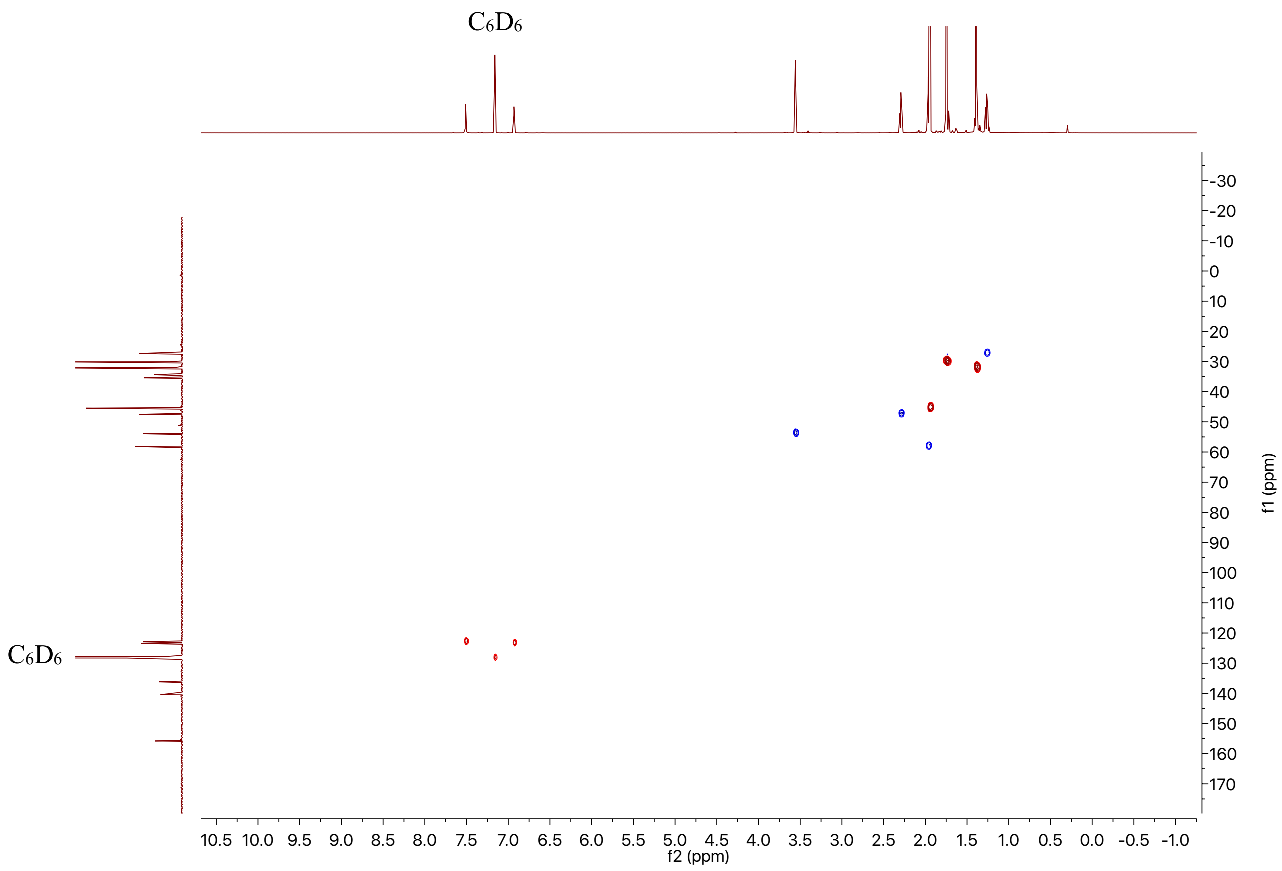

Figure S3. HSQC NMR (C_6D_6, 125 MHz) spectrum of **1**H.

2H:

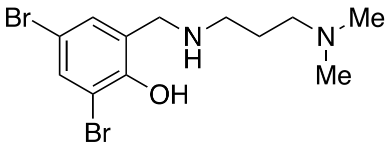

To a solution of 3,5-dibromosalicylaldehyde (0.56 g, 2 mmol) in MeOH (5 mL), 3-(dimethylamino)-1-propylamine (0.25 mL, 2 mmol) was added dropwise and the resulting solution was stirred for 3 h at room temperature. $NaBH_4$ (0.19 g, 5 mmol, 2.5 equivalents) was added portion-wise with stirring, observing rapid precipitation of a white powder in a colourless solution, which was stirred for 1 h at room temperature. The reaction mixture was quenched with deionized H_2O (5 mL) and the solvent concentrated *in vacuo*. A white powder was isolated by Buchner filtration, washed with deionized H_2O (4 × 5 mL) and dried *in vacuo*.

^1H NMR (C_6D_6, 400 MHz): δ = 7.60 (d, *J* = 2 Hz, 1H; ArH), 6.71 (d, *J* = 2 Hz, 1H; ArH), 3.06 (s, 2H; Ar-CH_2), 2.05 (t, *J* = 6 Hz, 2H; CH_2), 1.89 (s, 6H; $N(CH_3)_2$), 1.87 (t, *J* = 6 Hz, 2H; CH_2), 1.10 (p, *J* = 6 Hz, 2H; CH_2). *N.B.* Unaccounted for -OH and -NH resonances, indicative of rapid proton exchange in solution. HSQC analysis assisted with peak assignment.

$^{13}C\{^1H\}$ NMR (C_6D_6, 125 MHz): δ = 155.8, 134.3, 130.2, 125.5, 112.0, 110.3 (Ar), 58.3 (CH_2), 52.2 (Ar-CH_2), 47.6 (CH_2), 45.4 ($N(CH_3)_2$), 26.6 (CH_2). *N.B.* HSQC analysis assisted with peak assignment.

Yield = 0.55 g, 76%

ESI-MS (+ve, MeOH): Calculated m/z $[C_{12}H_{19}Br_2N_2O]^+$ = 366.9844; found m/z = 366.9823.

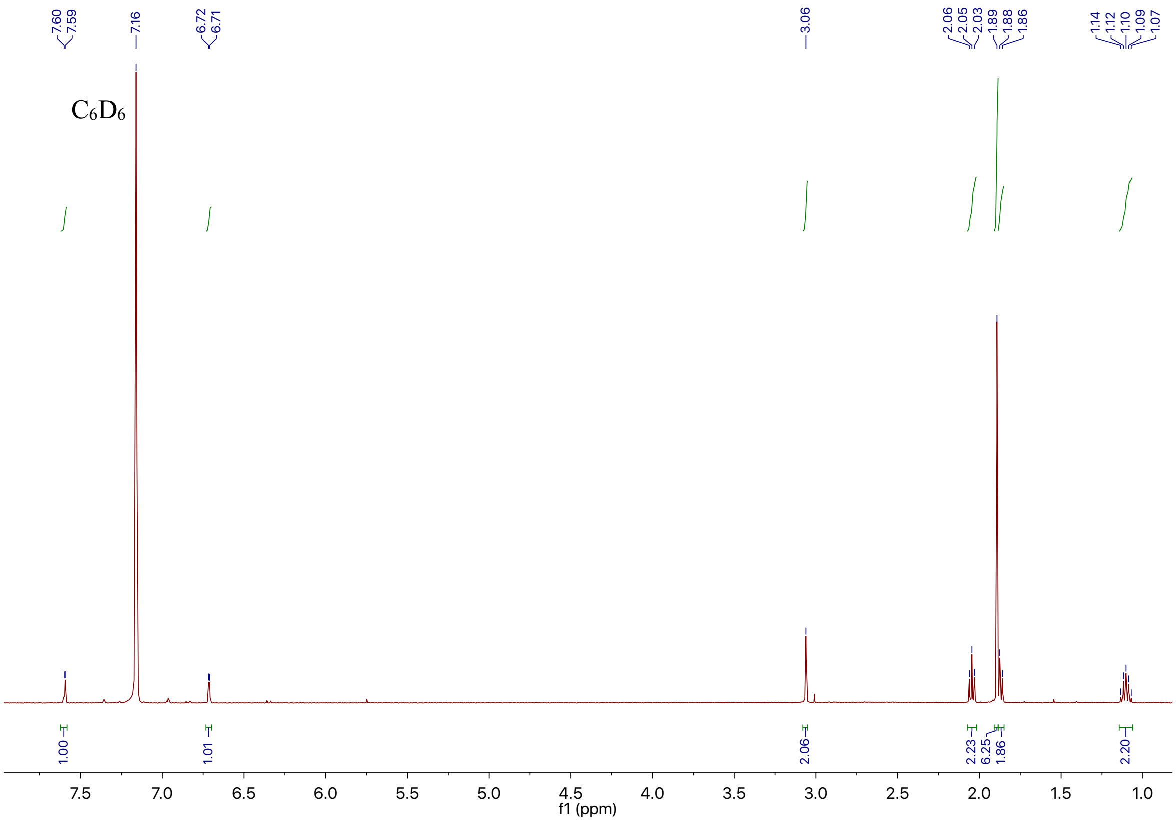

Figure S4. ^{1}H NMR (C_6D_6, 400 MHz) spectrum of **2**H.

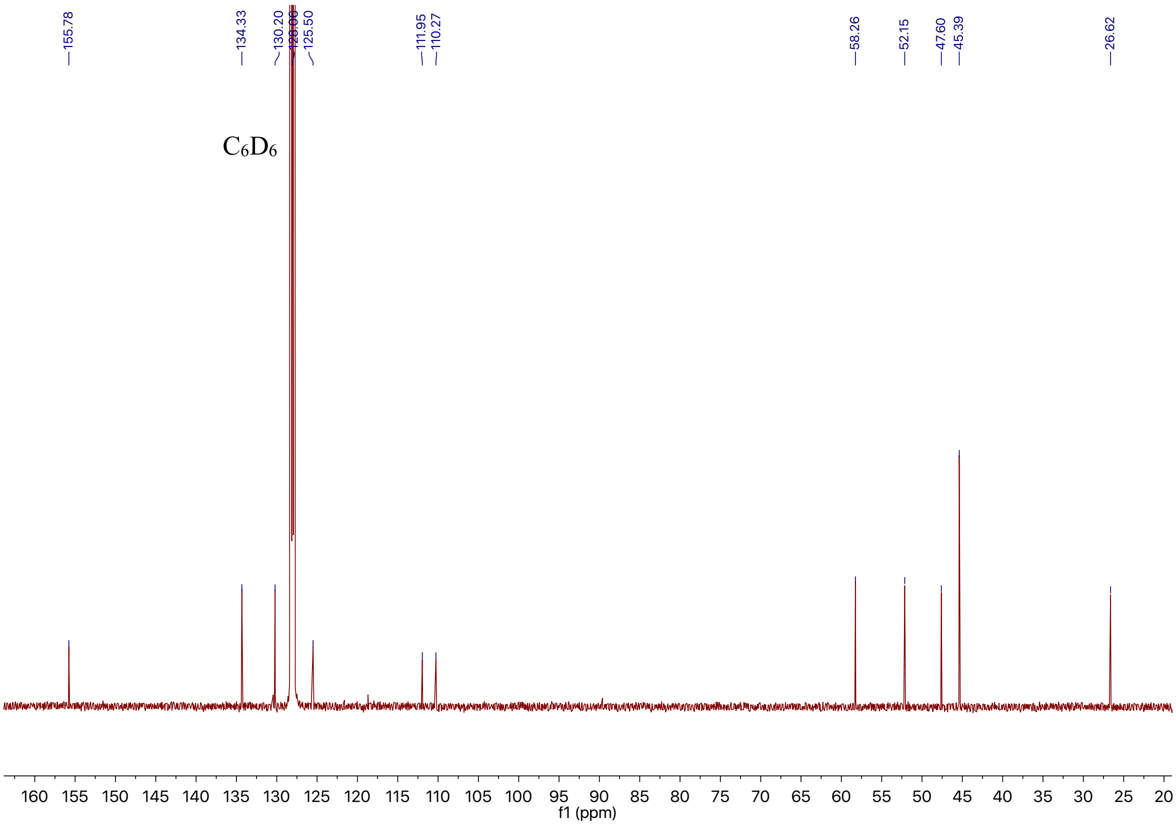

Figure S5. ^{13}C{^{1}H} NMR (C_6D_6, 125 MHz) spectrum of **2**H.

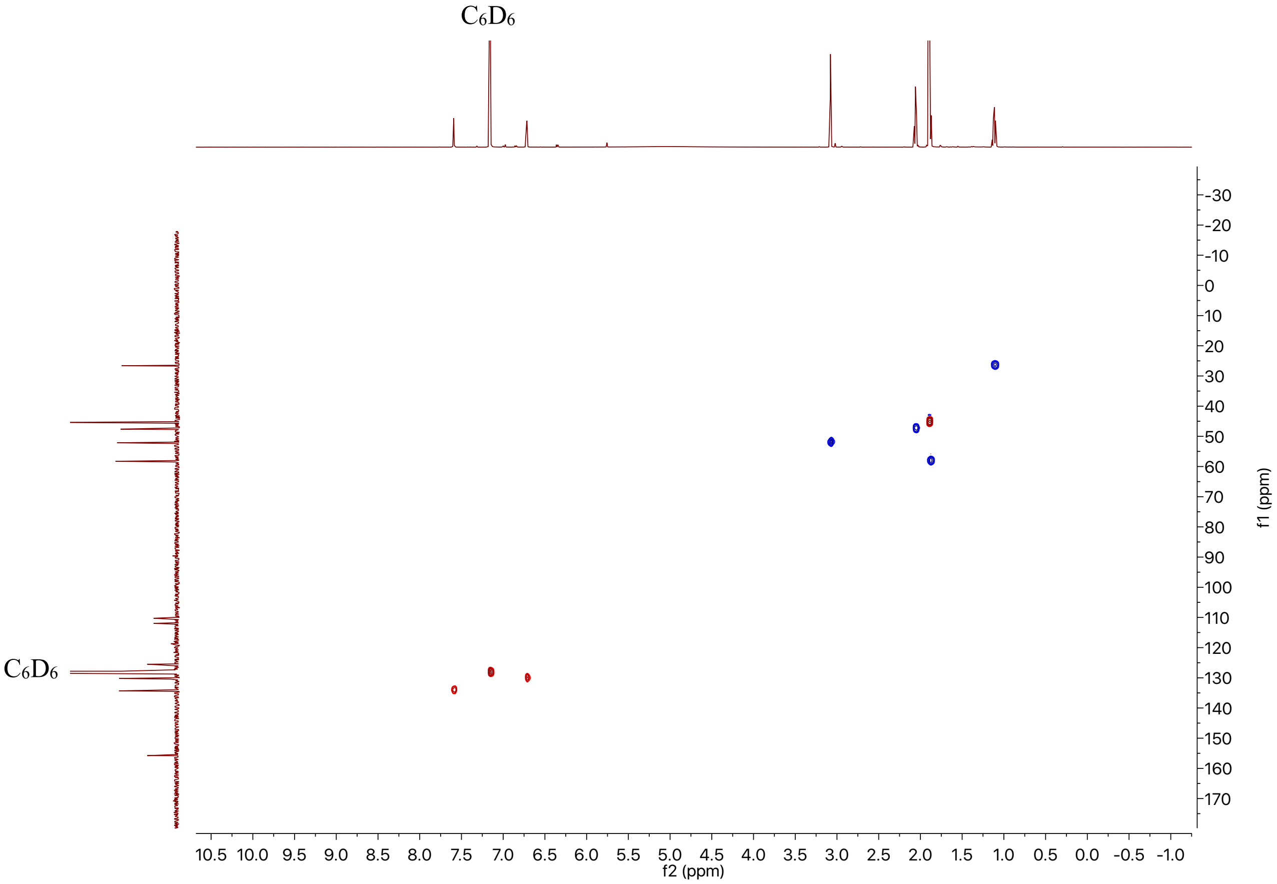

Figure S6. HSQC NMR (C_6D_6, 125 MHz) spectrum of **2**H.

3H:

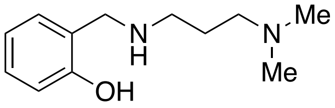

To a solution of salicylaldehyde (0.21 mL, 2 mmol) in MeOH (5 mL), 3-(dimethylamino)-1-propylamine (0.25 mL, 2 mmol) was added dropwise and the resulting solution was stirred for 3 h at room temperature. $NaBH_4$ (0.38 g, 10 mmol, 5 equivalents) was added portion-wise and the solution was stirred for 2 h at room temperature, observing discolouration to a cloudy colourless solution. The reaction mixture was quenched with deionized H_2O (5 mL) and the solvent removed *in vacuo*. The resulting oil was redissolved in DCM (30 mL), washed with deionized H_2O (3 × 30 mL) and the solvent removed *in vacuo*. The product was dried *in vacuo* for 1 h and collected as a pale-yellow oil.

^{1}H NMR ($CDCl_3$, 400 MHz): δ = 7.15 (td, *J* = 8, 2 Hz, 1H; ArH), 6.98 (dd, *J* = 7, 1 Hz, 1H; ArH), 6.82 (dd, *J* = 8, 1 Hz, 1H; ArH), 6.76 (td, *J* = 7, 1 Hz, 1H; ArH), 3.98 (s, 2H; Ar-CH_2), 2.73 (t, *J* = 7 Hz, 2H; CH_2), 2.33 (t, *J* = 7 Hz, 2H; CH_2), 2.20 (s, 6H; $N(CH_3)_2$), 1.70 (p, *J* = 7 Hz, 2H; CH_2). *N.B.* Unaccounted for -OH and -NH resonances, indicative of rapid proton exchange in solution. HSQC analysis assisted with peak assignment.

^{13}C{^{1}H} NMR ($CDCl_3$, 125 MHz): δ = 158.6, 128.7, 128.4, 122.7, 119.0, 116.5 (Ar), 58.2 (CH_2), 53.0 (Ar-CH_2), 47.8 (CH_2), 45.7 ($N(CH_3)_2$), 27.4 (CH_2). *N.B.* HSQC analysis assisted with peak assignment.

Yield = 0.41g, 98%

ESI-MS (+ve, MeOH): Calculated m/z $[C_{12}H_{21}N_2O]^+$ = 209.1654; found m/z = 209.1640.

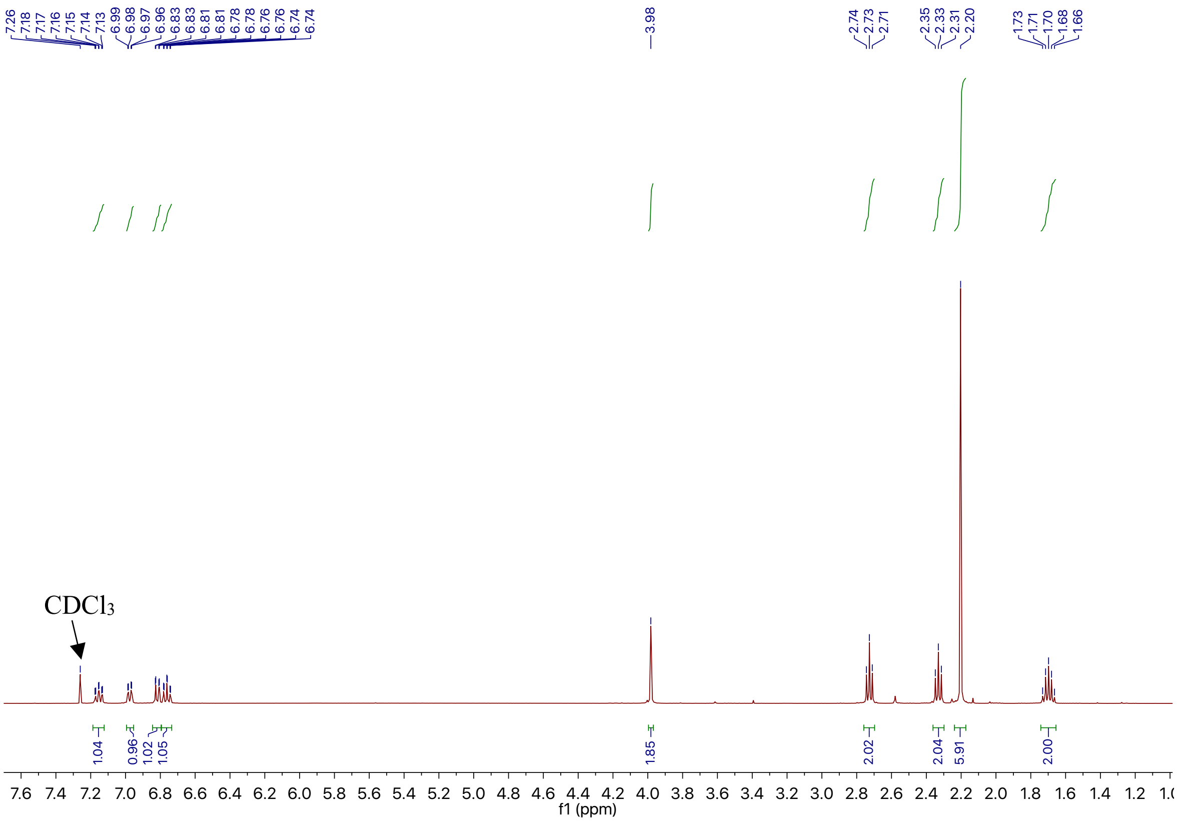

Figure S7. ^{1}H NMR ($CDCl_3$, 400 MHz) spectrum of **3**H.

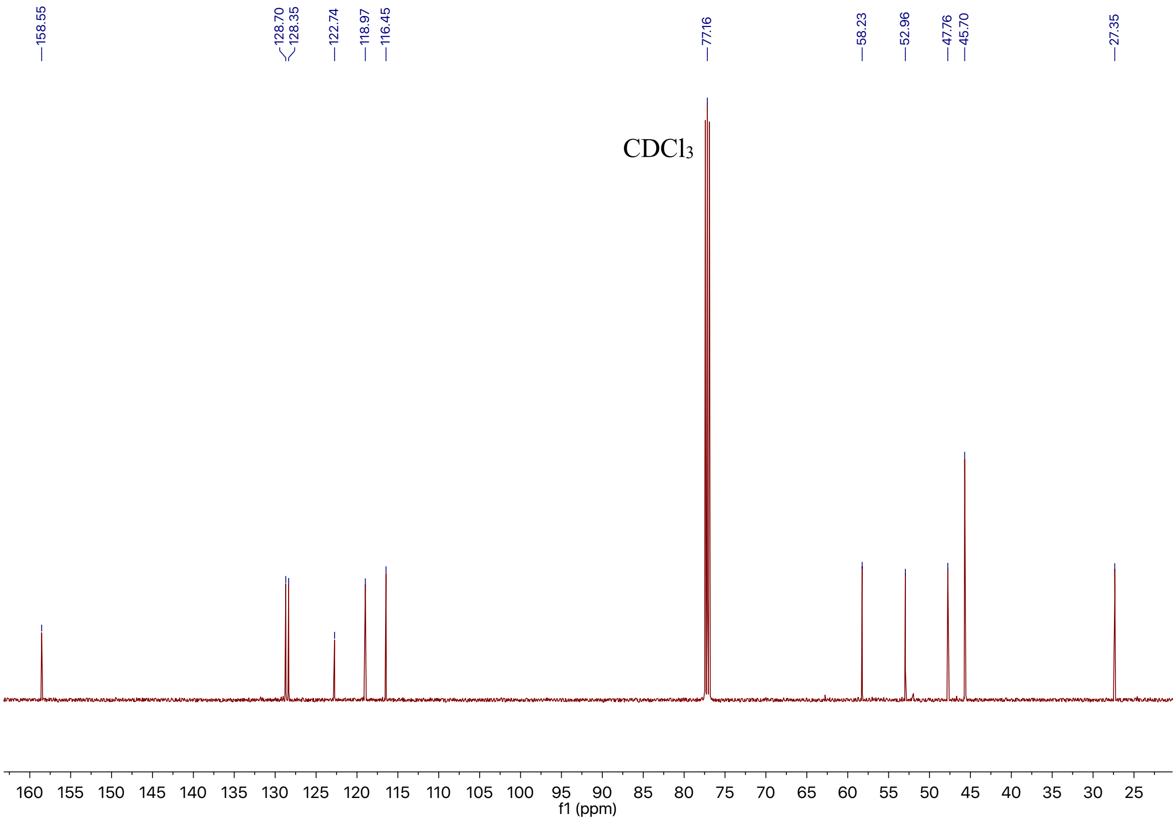

Figure S8. $^{13}C\{^1H\}$ NMR ($CDCl_3$, 125 MHz) spectrum of **3**H.

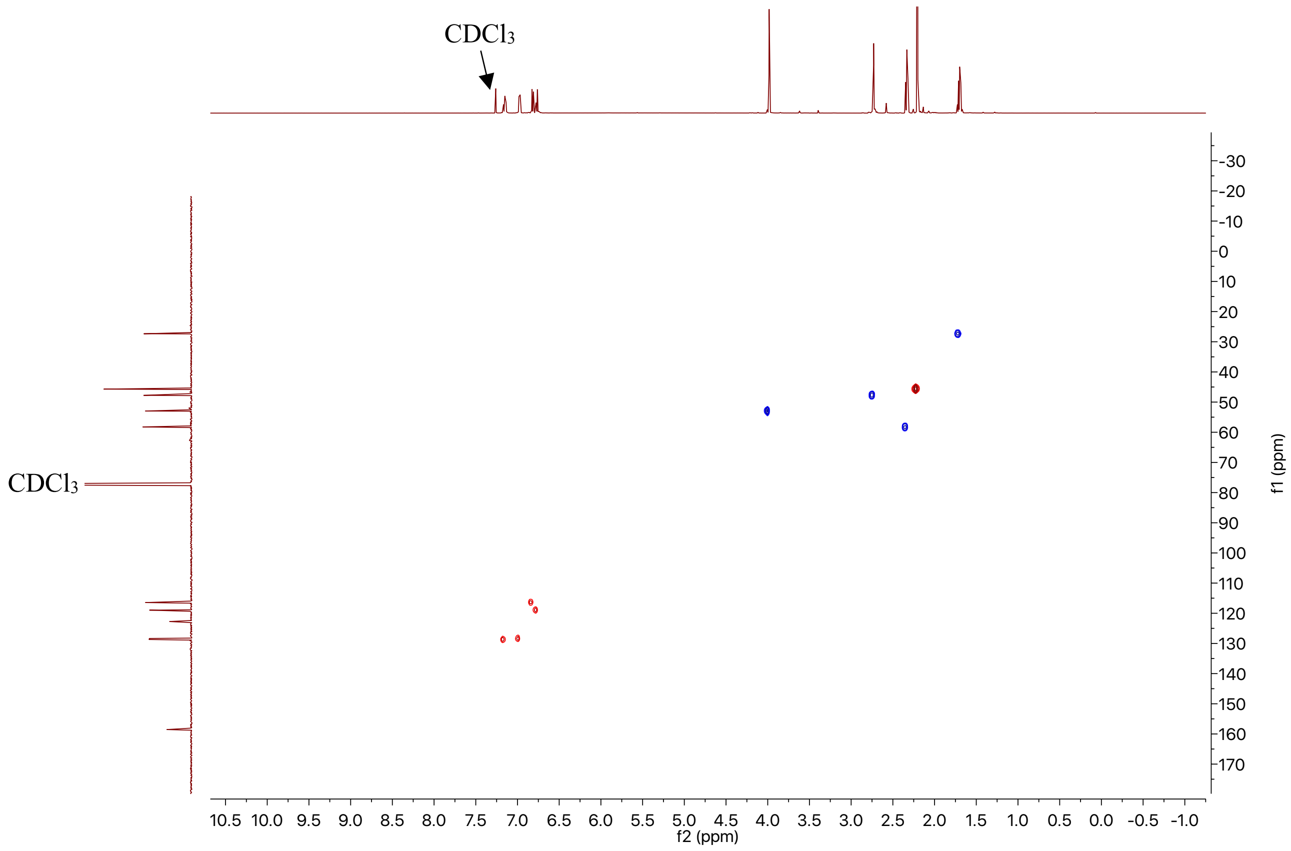

Figure S9. HSQC NMR ($CDCl_3$, 125 MHz) spectrum of **3**H.

4H:

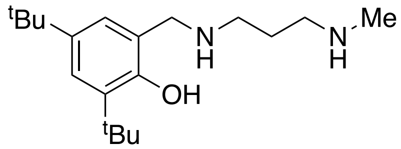

To a solution of 3,5-di-*tert*-butyl-2-hydroxybenzaldehyde (0.47 g, 2 mmol) in MeOH (5 mL), 3-(methylamino)propylamine (0.21 mL, 2 mmol) was added dropwise and the resulting solution was stirred for 1.5 h at 0 °C. $NaBH_4$ (0.38 g, 10 mmol, 5 equivalents) was added portion-wise and the solution was stirred for 3 h at room temperature, observing complete discolouration. The reaction mixture was quenched with deionized H_2O (5 mL) and the solvent removed *in vacuo*. The resulting oil was redissolved in DCM (30 mL), washed with deionized H_2O (3 × 30 mL) and the solvent removed *in vacuo*. The product was dried *in vacuo* for 1 h and collected as a white oil.

^1H NMR ($CDCl_3$, 400 MHz): δ = 7.22 (d, *J* = 3 Hz, 1H; ArH), 6.86 (d, *J* = 3 Hz, 1H; ArH), 3.95 (s, 2H; Ar-CH_2), 2.76 (t, *J* = 7 Hz, 2H; CH_2), 2.66 (t, *J* = 7 Hz, 2H; CH_2), 2.42 (s, 3H; N(CH_3)), 1.73 (p, *J* = 7 Hz, 2H; CH_2), 1.42 (s, 9H; C(CH_3)$_3$), 1.28 (s, 9H; C(CH_3)$_3$). *N.B.* Unaccounted for -OH and -NH resonances, indicative of rapid proton exchange in solution. HSQC analysis assisted with peak assignment.

$^{13}C\{^1H\}$ NMR ($CDCl_3$, 125 MHz): δ = 154.9, 140.5, 135.9, 123.2, 123.0, 122.2 (Ar), 53.8 (Ar-CH_2), 50.4, 47.3 (CH_2), 36.8 (N(CH_3)), 35.0, 34.3 (C(CH_3)$_3$), 31.8 (CH_3), 29.8 (CH_2), 29.8 (CH_3). *N.B.* HSQC analysis assisted with peak assignment.

Yield = 0.48 g, 79%

ESI-MS (+ve, MeOH): Calculated m/z $[C_{19}H_{35}N_2O]^+$ = 307.2749; found m/z = 307.2782.

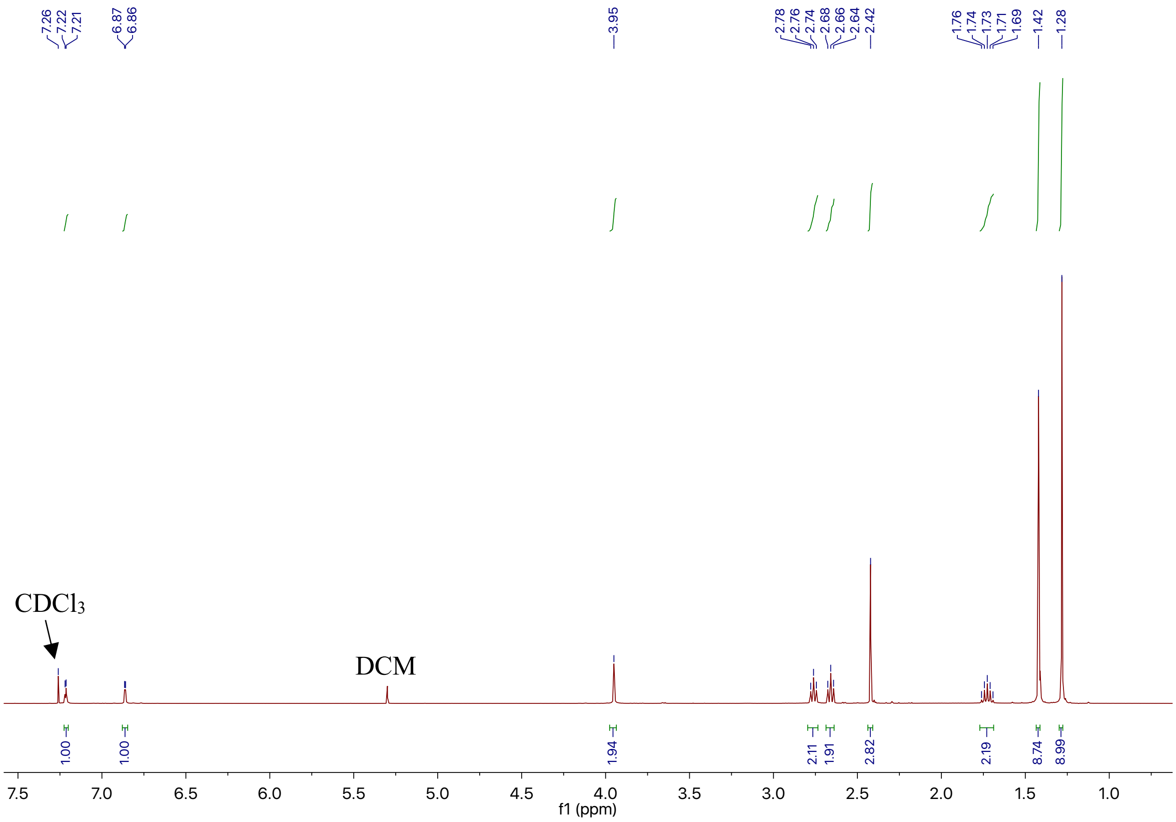

Figure S10. ^{1}H NMR ($CDCl_3$, 400 MHz) spectrum of **4**H.

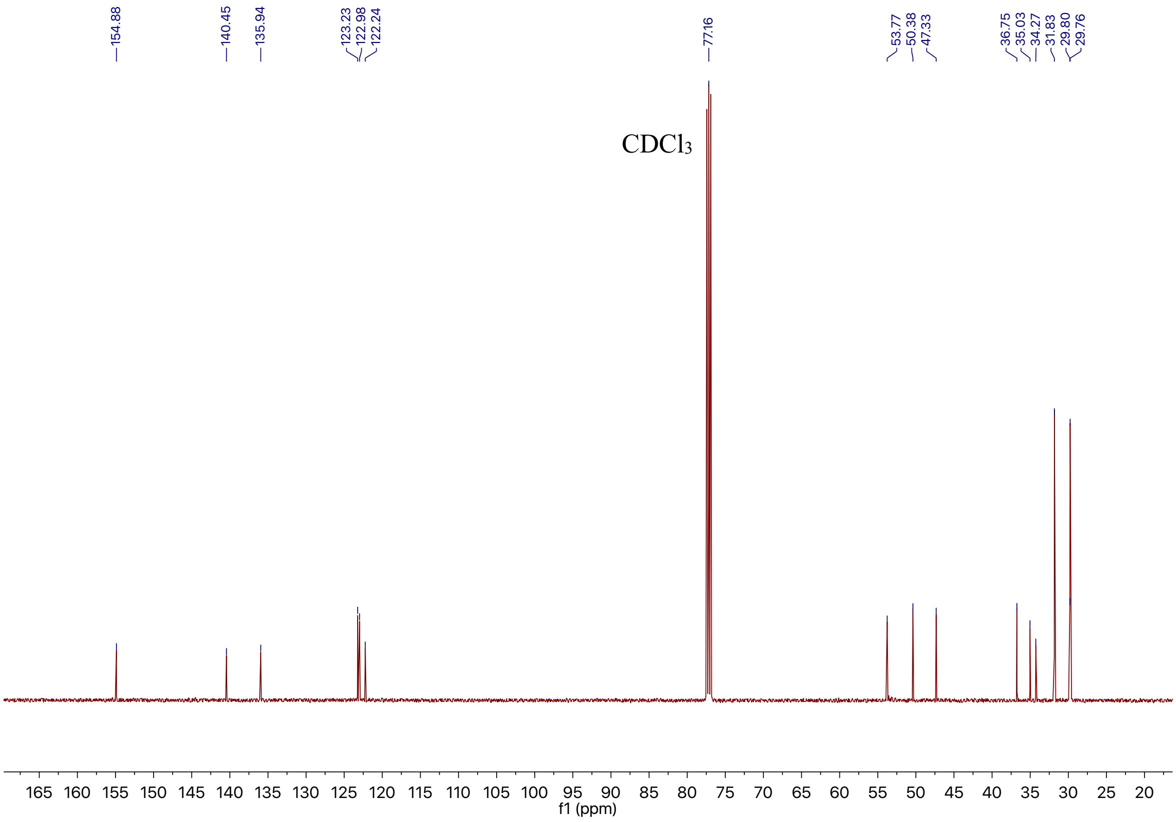

Figure S11. $^{13}C\{^{1}H\}$ NMR ($CDCl_3$, 125 MHz) spectrum of **4**H.

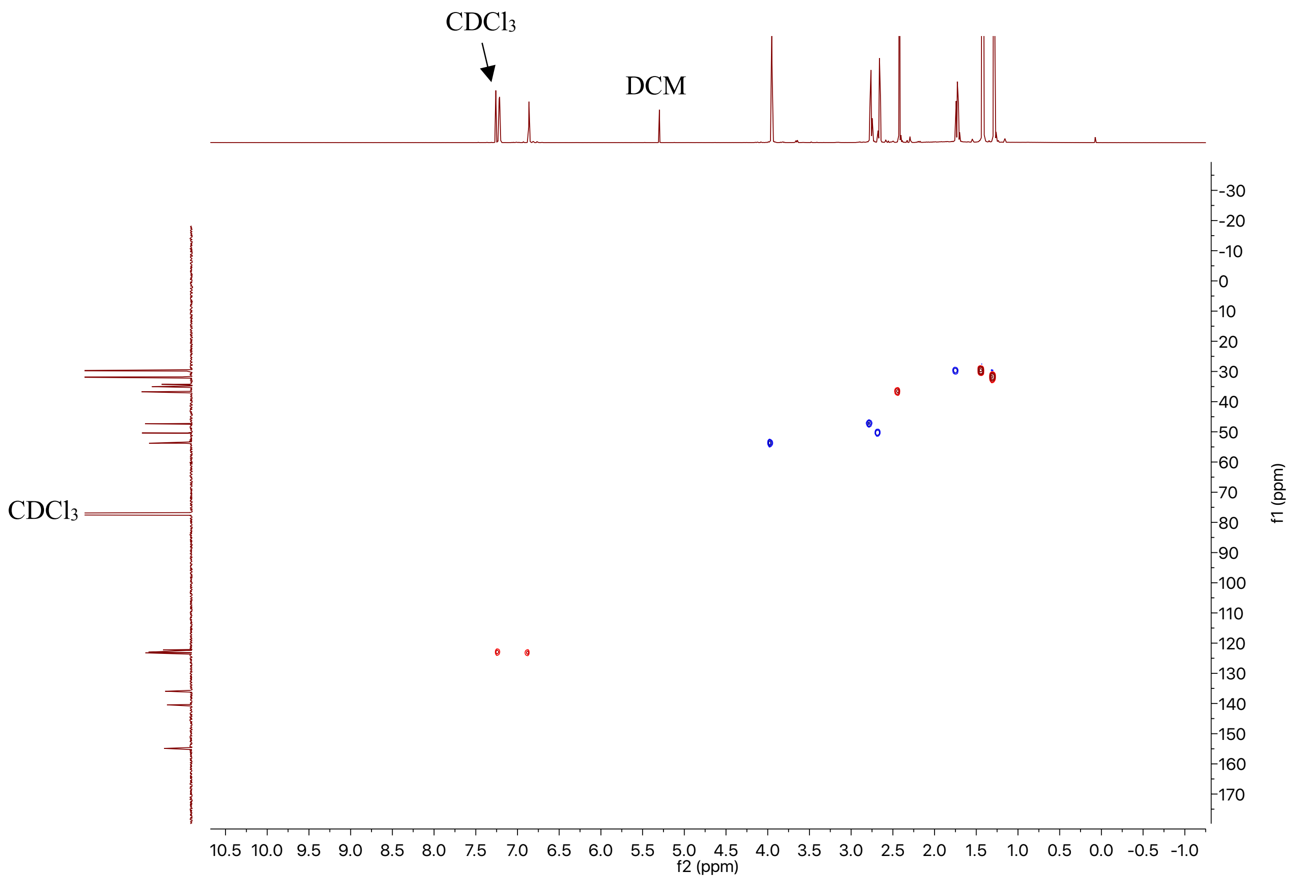

Figure S12. HSQC NMR ($CDCl_3$, 125 MHz) spectrum of **4**H.

5H:

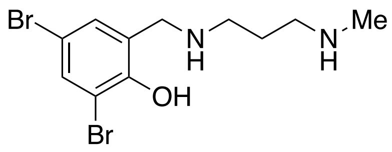

To a solution of 3,5-dibromosalicylaldehyde (0.56 g, 2 mmol) in MeOH (5 mL), 3-(methylamino)propylamine (0.21 mL, 2 mmol) was added dropwise and the resulting solution was stirred for 1.5 h at 0 °C. $NaBH_4$ (0.19 g, 5 mmol, 2.5 equivalents) was added portion-wise and the solution was stirred for 3 h at room temperature, observing complete discolouration. The reaction mixture was quenched with deionized H_2O (5 mL), observing rapid precipitation of a white powder. The solvent was removed *in vacuo*, the white solid redissolved in DCM (30 mL), washed with deionized H_2O (3 × 30 mL) and the solvent removed *in vacuo*. The product was dried *in vacuo* for 1 h and collected as a pale-yellow solid.

^{1}H NMR ($CDCl_3$, 400 MHz): δ = 7.53 (d, *J* = 2 Hz, 1H; ArH), 7.04 (d, *J* = 2 Hz, 1H; ArH), 3.95 (s, 2H; Ar-CH_2), 2.75 (t, *J* = 7 Hz, 2H; CH_2), 2.68 (t, *J* = 7 Hz, 2H; CH_2), 2.41 (s, 3H; N(CH_3)), 1.71 (p, *J* = 7 Hz, 2H; CH_2). *N.B.* Unaccounted for -OH and -NH resonances, indicative of rapid proton exchange in solution. HSQC analysis assisted with peak assignment.

^{13}C{^{1}H} NMR ($CDCl_3$, 125 MHz): δ = 155.2, 134.0, 130.1, 125.1, 111.5, 110.0 (Ar), 52.4 (Ar-CH_2), 50.8, 48.0 (CH_2), 36.6 (N(CH_3)), 28.9 (CH_2). *N.B.* HSQC analysis assisted with peak assignment.

Yield = 0.32 g, 45%

ESI-MS (+ve, MeOH): Calculated m/z $[C_{11}H_{17}N_2O_1Br_2]^+$ = 350.9708; found m/z = 350.9744.

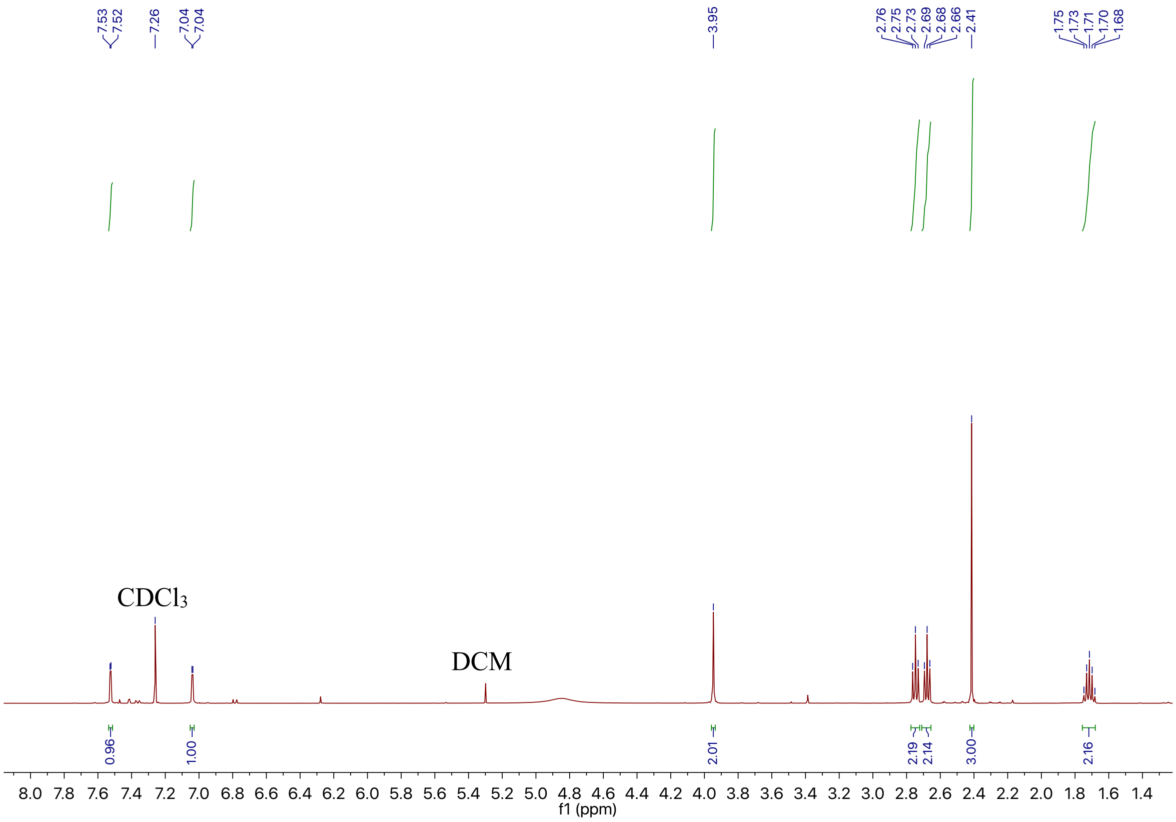

Figure S13. ^{1}H NMR ($CDCl_3$, 400 MHz) spectrum of **5**H.

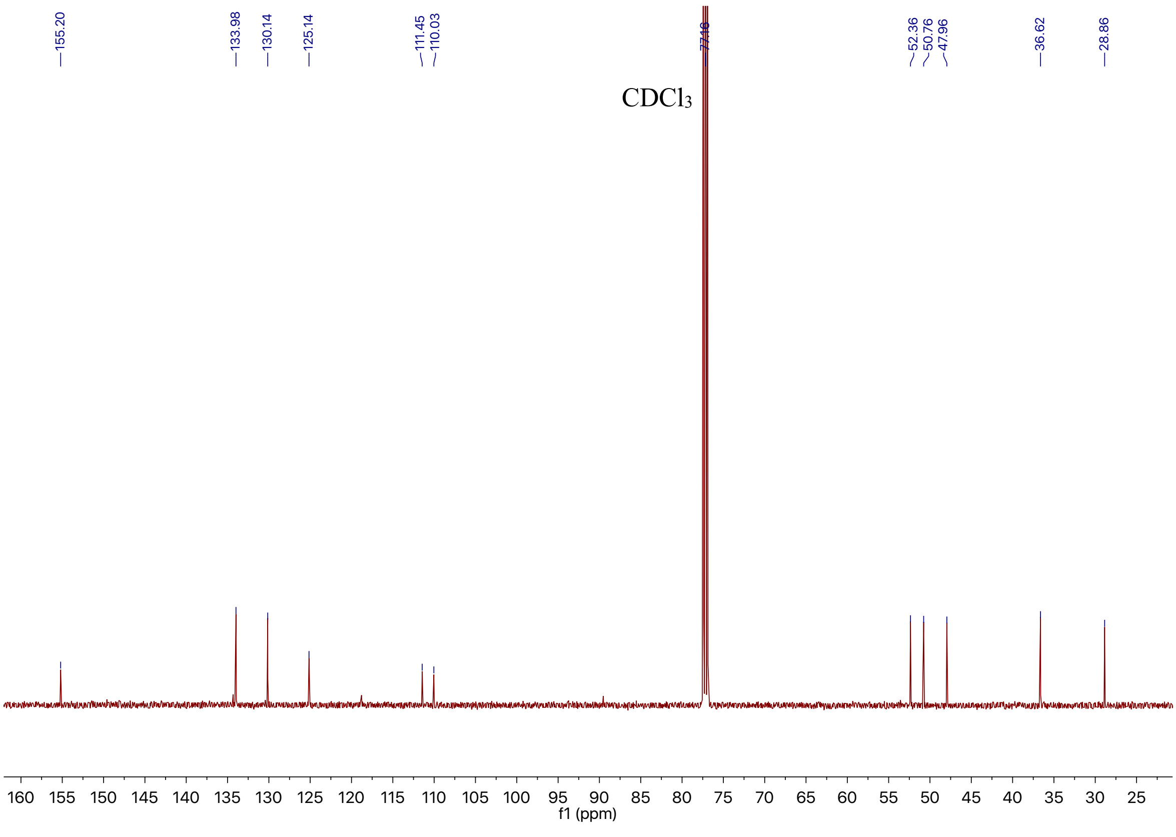

Figure S14. $^{13}C\{^{1}H\}$ NMR ($CDCl_3$, 125 MHz) spectrum of **5**H.

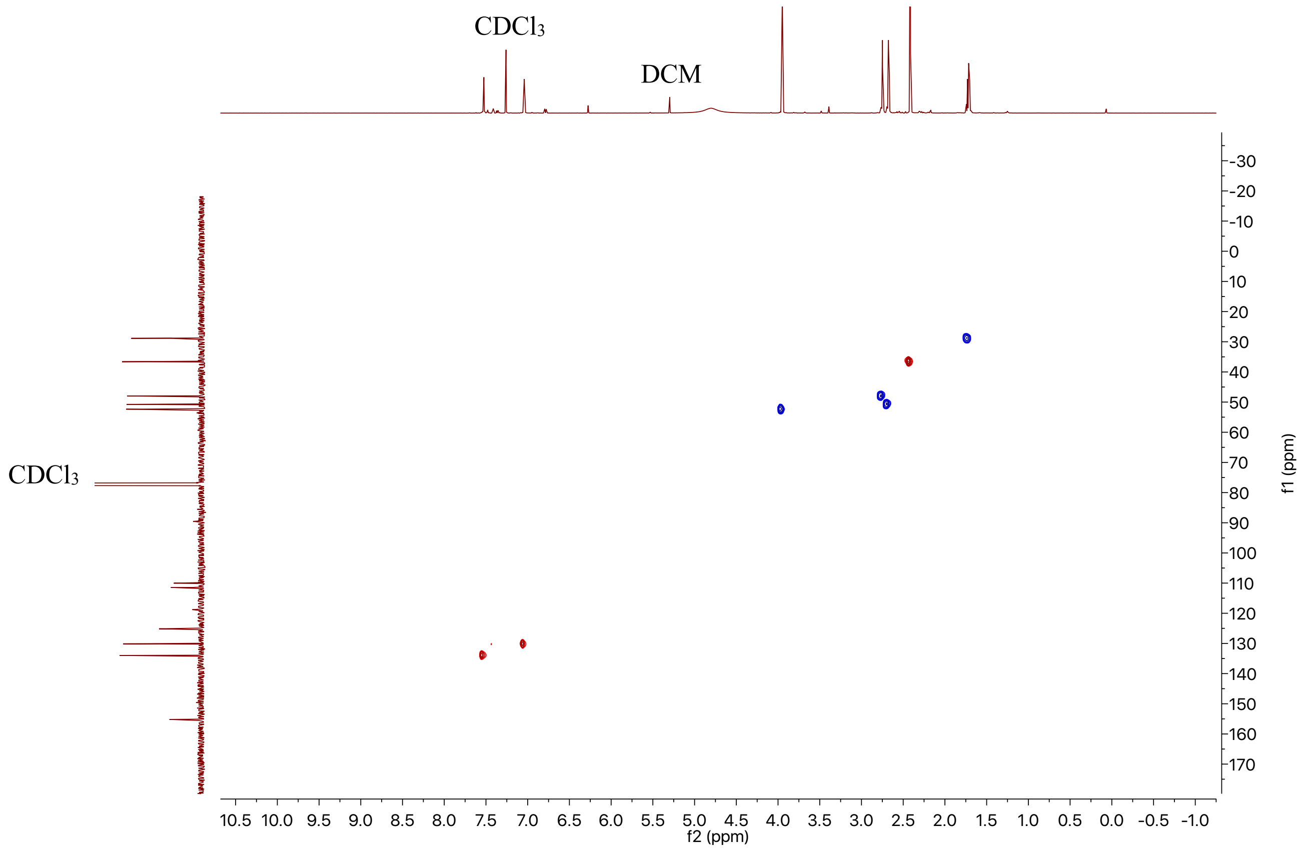

Figure S15. HSQC NMR ($CDCl_3$, 125 MHz) spectrum of **5**H.

6H:

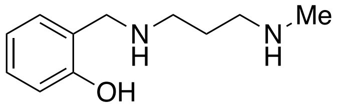

To a solution of salicylaldehyde (0.21 mL, 2 mmol) in MeOH (5 mL), 3-(methylamino)propylamine (0.21 mL, 2 mmol) was added dropwise and the resulting solution was stirred for 1.5 h at 0 °C. $NaBH_4$ (0.38 g, 10 mmol, 5 equivalents) was added portion-wise and the solution was stirred for 3 h at room temperature, observing complete discolouration. The reaction mixture was quenched with deionized H_2O (5 mL) and the solvent removed *in vacuo*. The resulting oil was redissolved in DCM (30 mL), washed with deionized H_2O (3 × 30 mL) and the solvent removed *in vacuo*. The product was dried *in vacuo* for 1 h and collected as a clear colourless oil.

^{1}H NMR ($CDCl_3$, 400 MHz): δ = 7.16 (td, *J* = 8, 2 Hz, 1H; ArH), 6.98 (d, *J* = 8 Hz, 1H; ArH), 6.82 (dd, *J* = 8, 1 Hz, 1H; ArH), 6.76 (td, *J* = 7, 1 Hz, 1H; ArH), 3.99 (s, 2H; Ar-CH_2), 2.75 (t, *J* = 7 Hz, 2H; CH_2), 2.66 (t, *J* = 7 Hz, 2H; CH_2), 2.41 (s, 3H; N(CH_3)), 1.72 (p, *J* = 7 Hz, 2H; CH_2). *N.B.* Unaccounted -OH and -NH resonances, indicative of rapid proton exchange in solution. HSQC analysis assisted with peak assignment. Relative peak area (RPA) of aromatic resonances higher than expected, indicative of residual product impurities, which remain unidentified. One possible impurity is the presence of a minor cyclic by-product (**6***H$_{cyc}$) generated *via* cyclisation of the salen (**6***H) prior to reduction. This is consistent with an additional singlet resonance at δ = 3.98 ppm corresponding to a methine proton (NCH). Purification by complexation pursued.

^{13}C{^{1}H} NMR ($CDCl_3$, 125 MHz): δ = 158.5, 128.7, 128.4, 122.7, 119.0, 116.5 (Ar), 52.93 (Ar-CH_2), 50.48, 47.49 (CH_2), 36.73 (N(CH_3)), 29.67 (CH_2). *N.B.* HSQC analysis assisted with peak assignment. Additional peaks observed due the presence of residual impurities. Purification by complexation pursued.

Yield = 0.19 g, 49%

ESI-MS (+ve, MeOH): Calculated m/z $[C_{11}H_{19}N_2O]^+$ = 195.1498 ; found m/z = 195.1488.

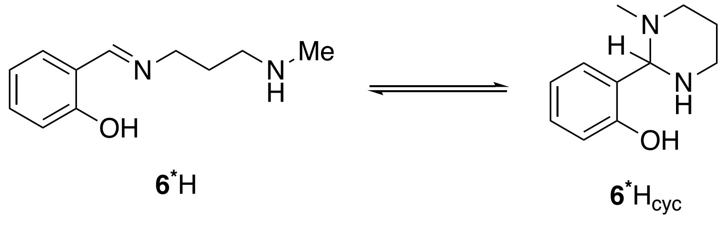

Scheme S1. Possible salen ligand cyclisation reaction affording 6*H$_{cyc}$ prior to reduction.

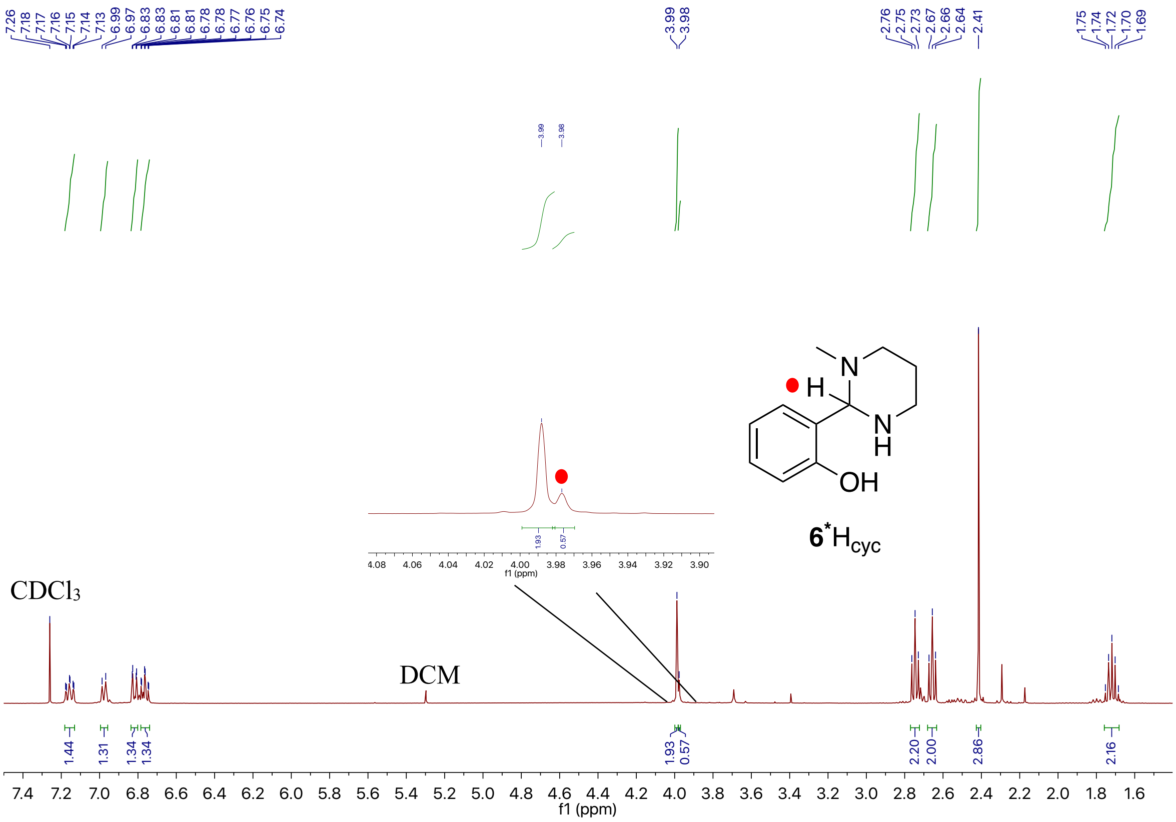

Figure S16. ^{1}H NMR ($CDCl_3$, 400 MHz) spectrum of **6**H.

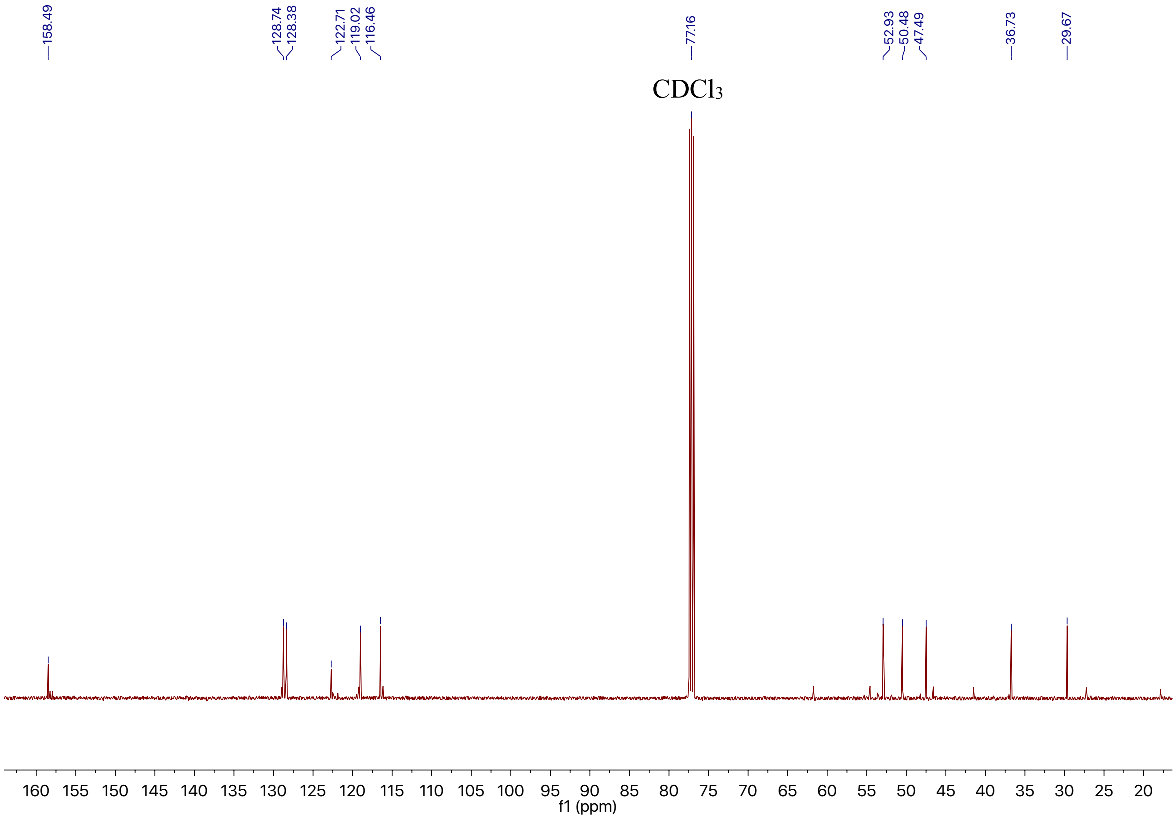

Figure S17. $^{13}C\{^{1}H\}$ NMR ($CDCl_3$, 125 MHz) spectrum of **6**H.

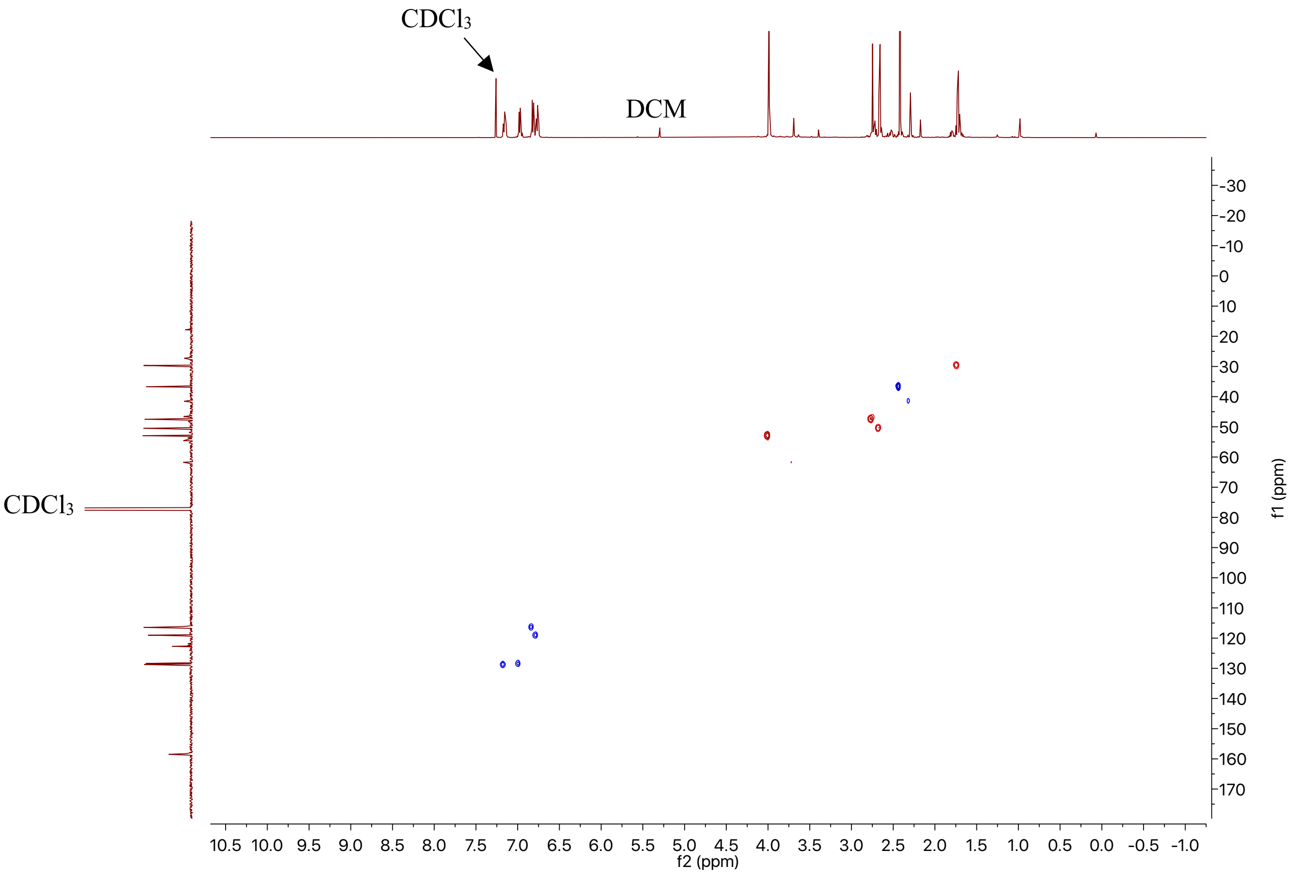

Figure S18. HSQC NMR ($CDCl_3$, 125 MHz) spectrum of **6**H.

2.2. Zn(II)-Complexes

Zn(**1**)Et:

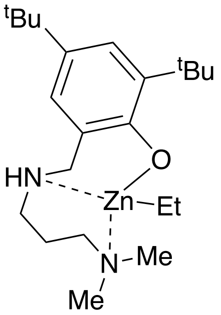

To a solution of **1**H (0.18 g, 0.55 mmol) in dry toluene (5.5 mL), $ZnEt_2$ (0.55 mL, 0.55 mmol, 1.0 M in hexane) was added dropwise with stirring. The resulting solution was stirred for 3 h at room temperature. The solvent was removed *in vacuo* to afford a white solid, which was washed in *n*-hexane (5 mL) for 1 h prior to recrystallisation. A white solid was isolated by cannula filtration and dried *in vacuo* for 3 h at 80 °C.

^{1}H NMR (C_6D_6, 500 MHz): δ = 7.64 (d, *J* = 3 Hz, 1H; ArH), 6.97 (d, *J* = 2 Hz, 1H; ArH), 3.46 (t, *J* = 11 Hz, 1H; Ar-CH), 3.22 (d, *J* = 12 Hz, 1H; Ar-CH), 2.16 – 2.06 (m, 1H; CH), 1.89 (br s, 16H; $N(CH_3)_2$; CH; $C(CH_3)_3$)), 1.85 – 1.79 (m, 2H; CH), 1.78 – 1.71 (m, 2H; CH), 1.54 (s, 9H; $C(CH_3)_3$), 1.52 (t, *J* = 8 Hz, 3H; Zn-Et), 1.11 – 0.95 (m, 2H; CH, NH), 0.62 – 0.52 (m, 1H; CH), 0.45 – 0.30 (m, 2H; Zn-Et). *N.B.* HSQC analysis assisted with -CH and -NH resonance assignment. Higher RPA than expected observed for δ = 1.85 – 1.79 (m, 2H; CH) and 1.78 – 1.71 (m, 2H; CH), which can likely be attributed to baseline broadening. Consequently, it is proposed both resonances correspond to a single -CH resonance (RPA = 1), consistent with HSQC analysis. Broad singlet observed at δ = 1.89, which could be attributed to overlapping $N(CH_3)_2$ and CH resonances with a $C(CH_3)_3$ singlet. This was treated as a single peak for RPA analysis. Peak broadening can likely be attributed to structural fluxionality.

$^{13}C\{^1H\}$ NMR (C_6D_6, 125 MHz): δ = 164.9, 138.4, 133.9, 125.1, 124.0, 121.9 (Ar), 60.9 (CH_2), 54.0 (Ar-CH_2), 48.7 (CH_2), 47.1 ($N(CH_3)_2$), 36.0, 34.3 ($C(CH_3)_3$), 32.5, 30.4 (CH_3), 22.8 (CH_2), 14.5 (CH_3; Zn-Et), -3.5 (CH_2; Zn-Et). *N.B.* HSQC analysis assisted with peak assignment. Low peak intensity observed for $N(CH_3)_2$ (δ = 47.1), indicative of fluxionality at the $^{13}C\{^1H\}$ NMR timescale, consistent with ^{1}H NMR analysis.

Yield = 0.13 g, 55%

Calculated for $C_{22}H_{40}N_2O_1Zn$: C, 63.83%; H, 9.74%; N, 6.77%. Found: C, 61.64%; H, 9.52%; N, 6.61%. Elemental analysis more consistent with a degree of hydroxide during analysis, indicating poor hydrolytic stability. Zn(**1**)OH, theoretical: C, 59.77%; H, 9.03%; N, 6.97%.

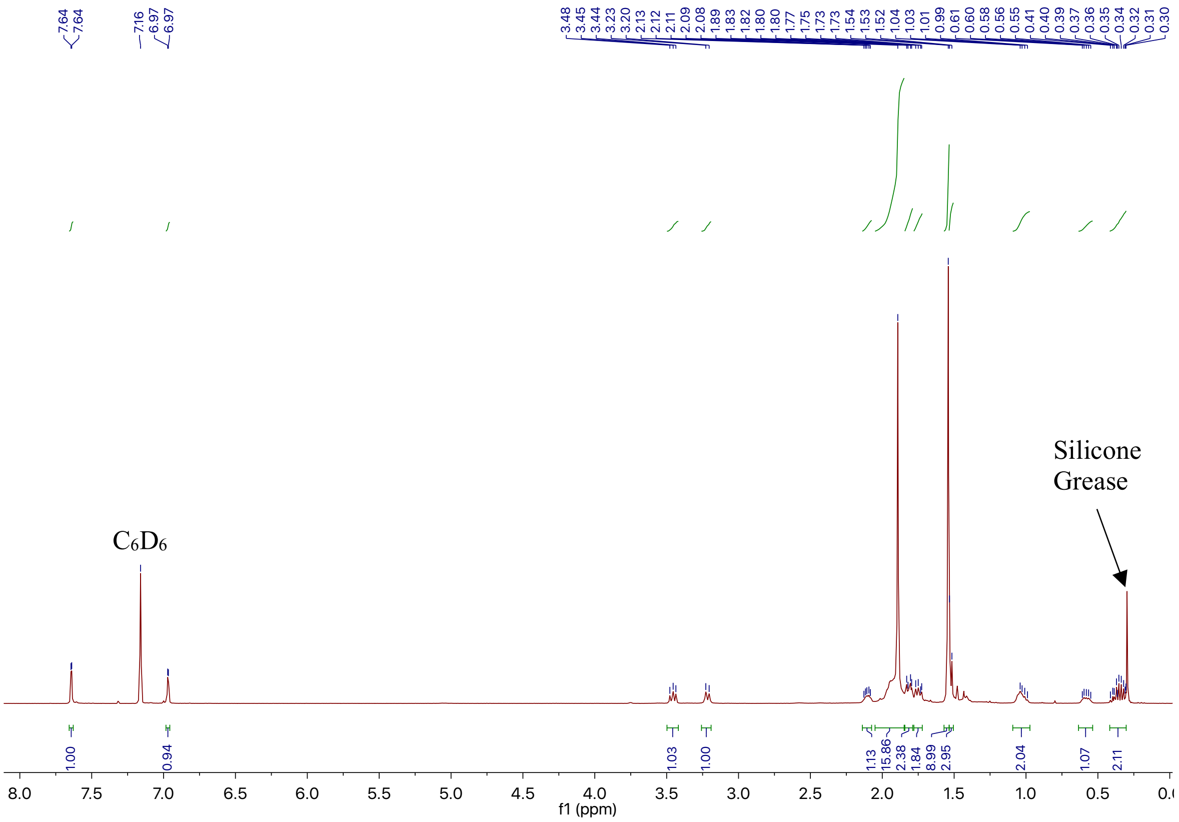

Figure S19. ^{1}H NMR (C_6D_6, 500 MHz) spectrum of Zn(**1**)Et.

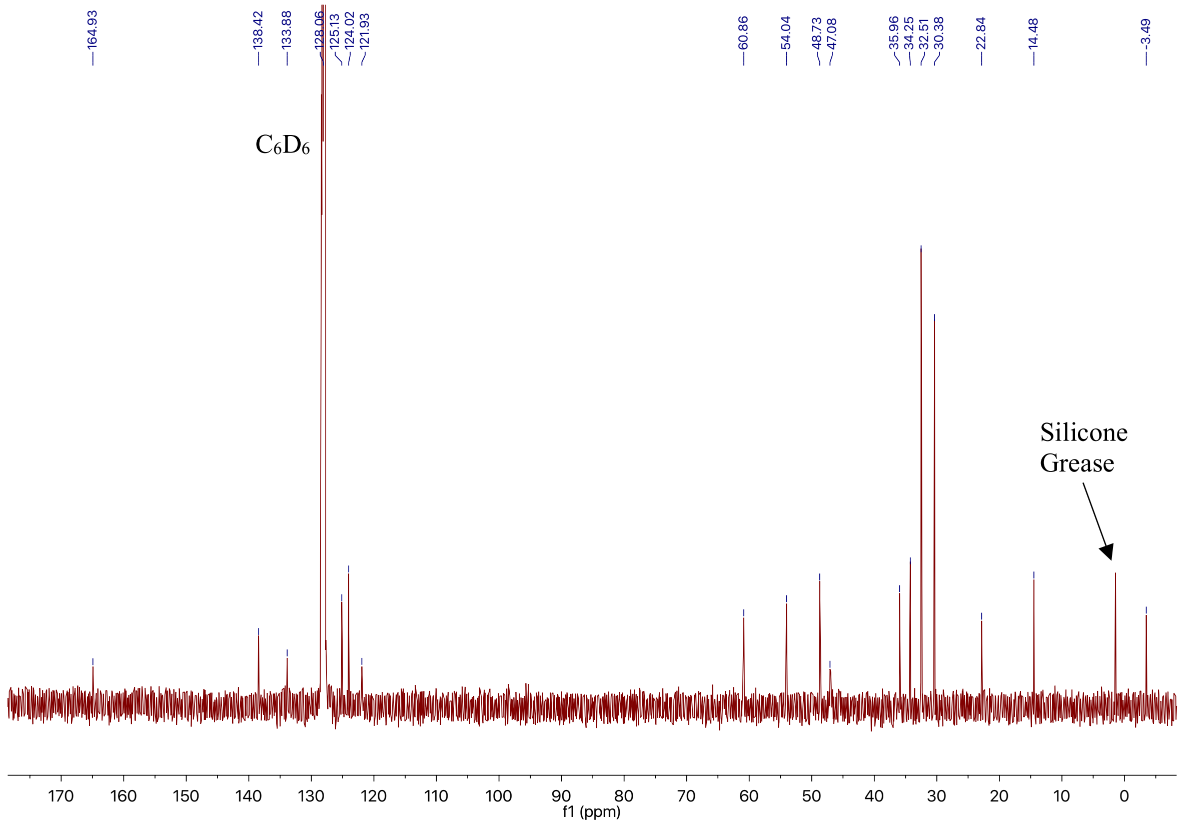

Figure S20. $^{13}C\{^{1}H\}$ NMR (C_6D_6, 125 MHz) spectrum of Zn(**1**)Et.

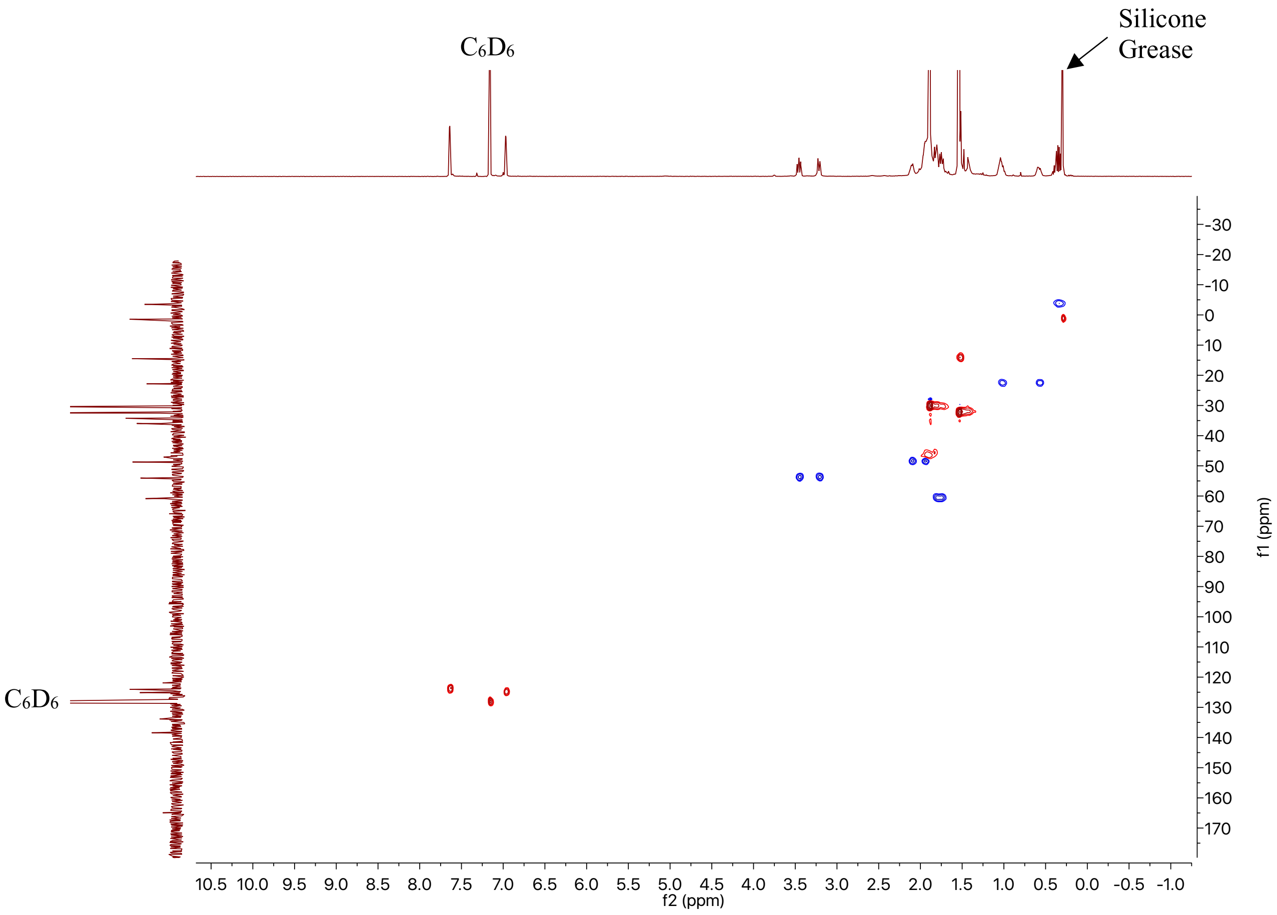

Figure S21. HSQC NMR (C_6D_6, 125 MHz) spectrum of Zn(**1**)Et.

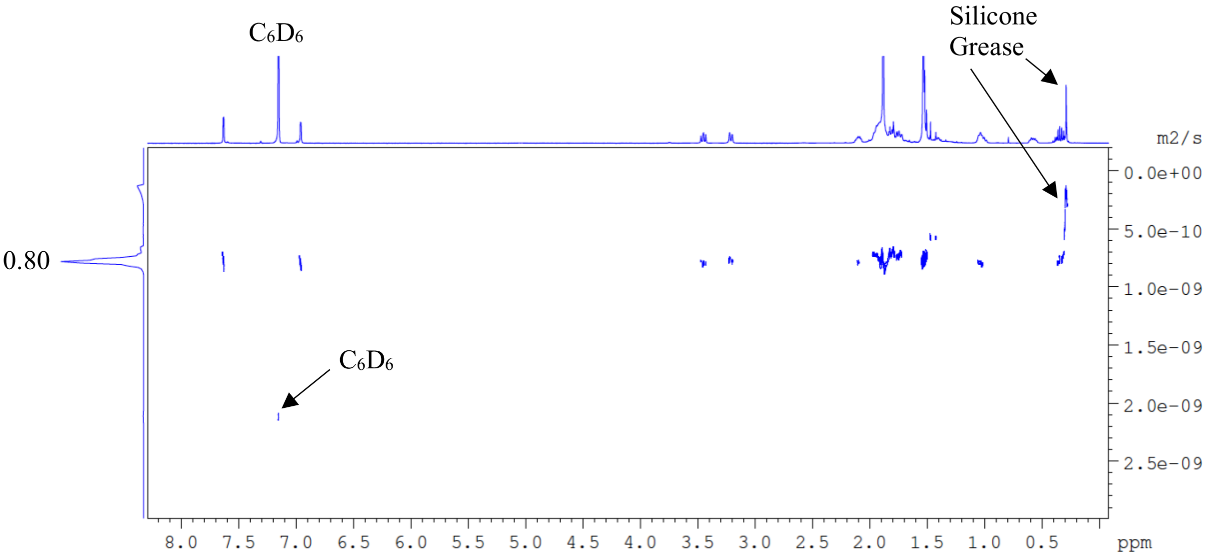

Figure S22. DOSY NMR (C_6D_6, 500 MHz) spectrum of Zn(**1**)Et, indicating only one species present in solution, with a diffusion constant of 0.80×10^{-9} m^2 s^{-1}.

Zn(**2**)$_2$:

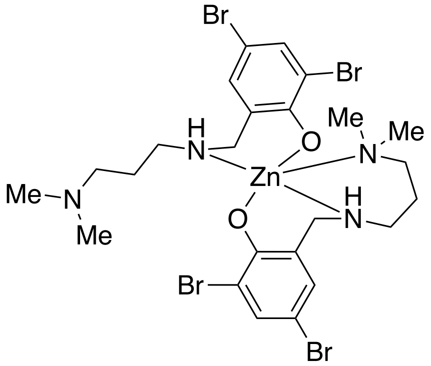

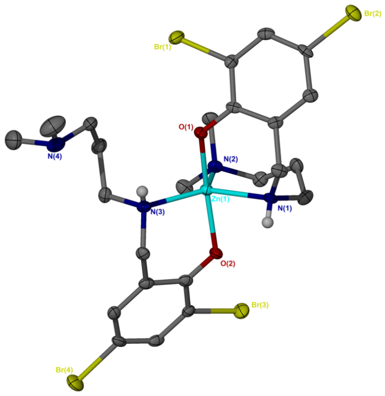

To a solution of **2**H (0.55 g, 1.5 mmol) in dry toluene (7.5 mL), $ZnEt_2$ (0.75 mL, 0.75 mmol, 1.0 M in hexane) was added dropwise with stirring, observing rapid precipitation of a white powder. The resulting suspension was stirred at 80 °C overnight under a static argon atmosphere. The product was redissolved upon vigorous heating with stirring and the flask transferred to a freezer. After 3 days at – 18 °C, a white solid powder was isolated by cannula filtration and dried *in vacuo* at 80 °C for 4 h.

^{1}H NMR ($CDCl_3$, 500 MHz): δ = 7.52 (d, J = 2 Hz, 1H; ArH), 7.02 (d, J = 3 Hz, 1H; ArH), 3.84 (br s, 2H; Ar-CH_2), 3.03 (br s, 2H; CH_2), 2.47 (s, 2H; CH_2), 2.24 (s, 6H; ($N(CH_3)_2$)), 1.79 (br s, 2H; CH_2). *N.B.* HSQC analysis assisted with -CH and -NH resonance assignment. Unaccounted for -NH resonance likely due to rapid proton exchange in solution. Evidence of peak broadening for -CH resonances, possibly indicating structural fluxionality.

^{13}C{^{1}H} NMR ($CDCl_3$, 125 MHz): δ = 161.9, 134.4, 132.0, 126.9, 115.7, 103.6 (Ar), 62.0 (CH_2), 52.0 (Ar-CH_2), 49.3 (CH_2), 46.8 ($N(CH_3)_2$), 24.0 (CH_2). *N.B.* HSQC analysis assisted with peak assignment. Poor CH_2 resonance intensity observed, indicative of fluxionality on the ^{13}C{^{1}H} NMR timescale, consistent with ^{1}H NMR analysis.

Yield = 0.36 g, 60%

Calculated for $C_{24}H_{34}Br_4N_4O_2Zn$: C, 36.23%; H, 4.31%; N, 7.04%. Found: C, 37.02%; H, 4.42%; N, 6.84%.

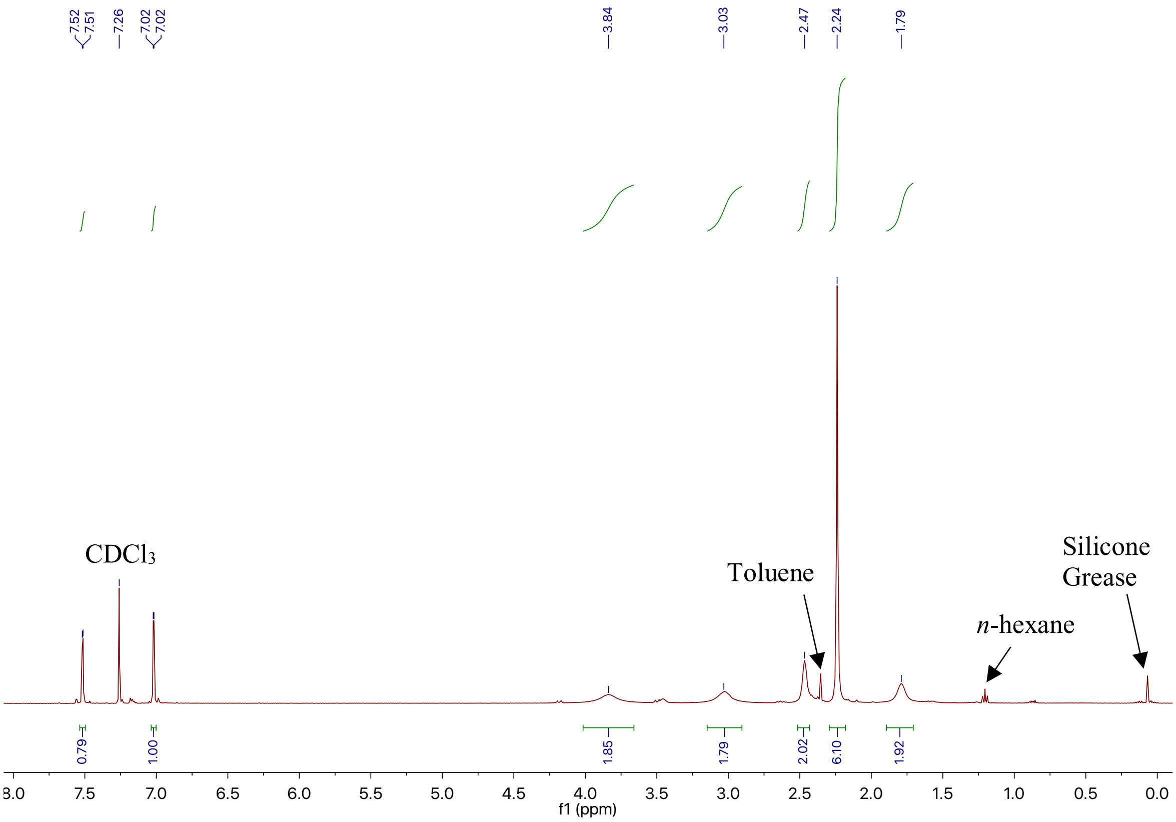

Figure S23. ^{1}H NMR ($CDCl_3$, 500 MHz) spectrum of Zn(**2**)$_2$.

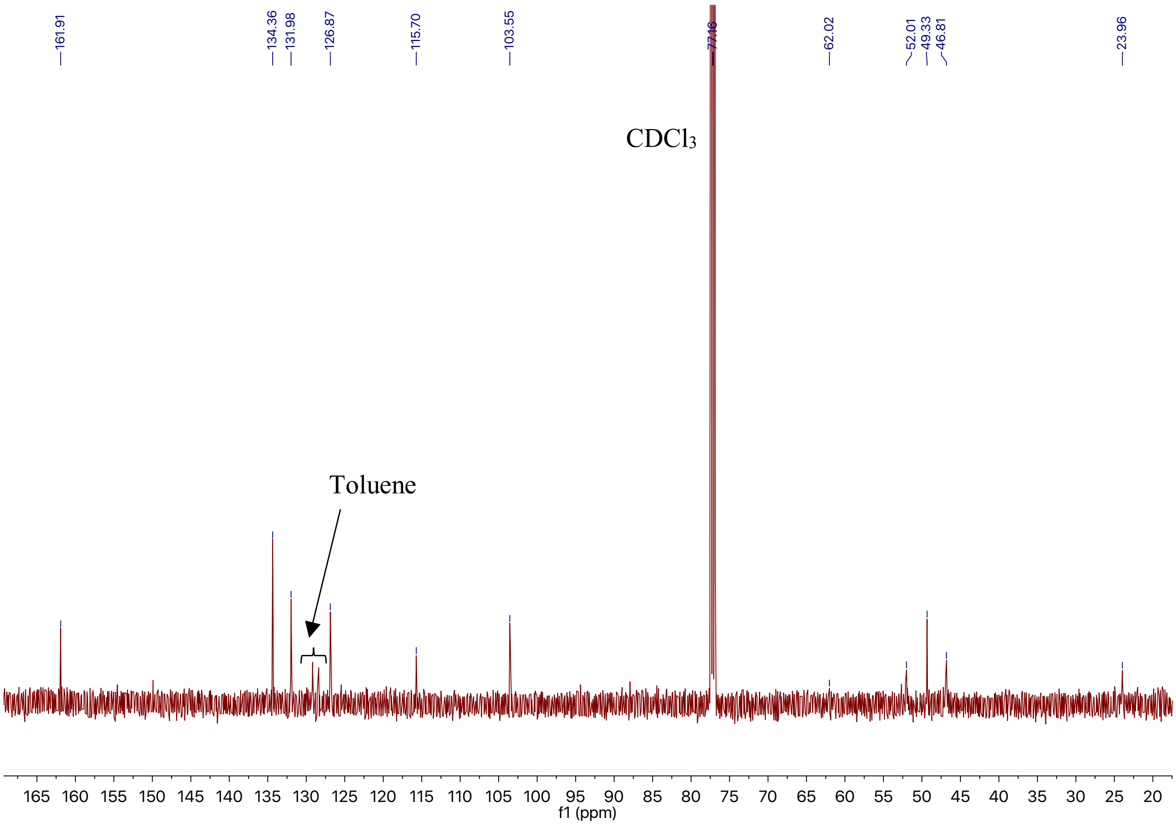

Figure S24. $^{13}C\{^1H\}$ NMR ($CDCl_3$, 125 MHz) spectrum of Zn(**2**)$_2$.

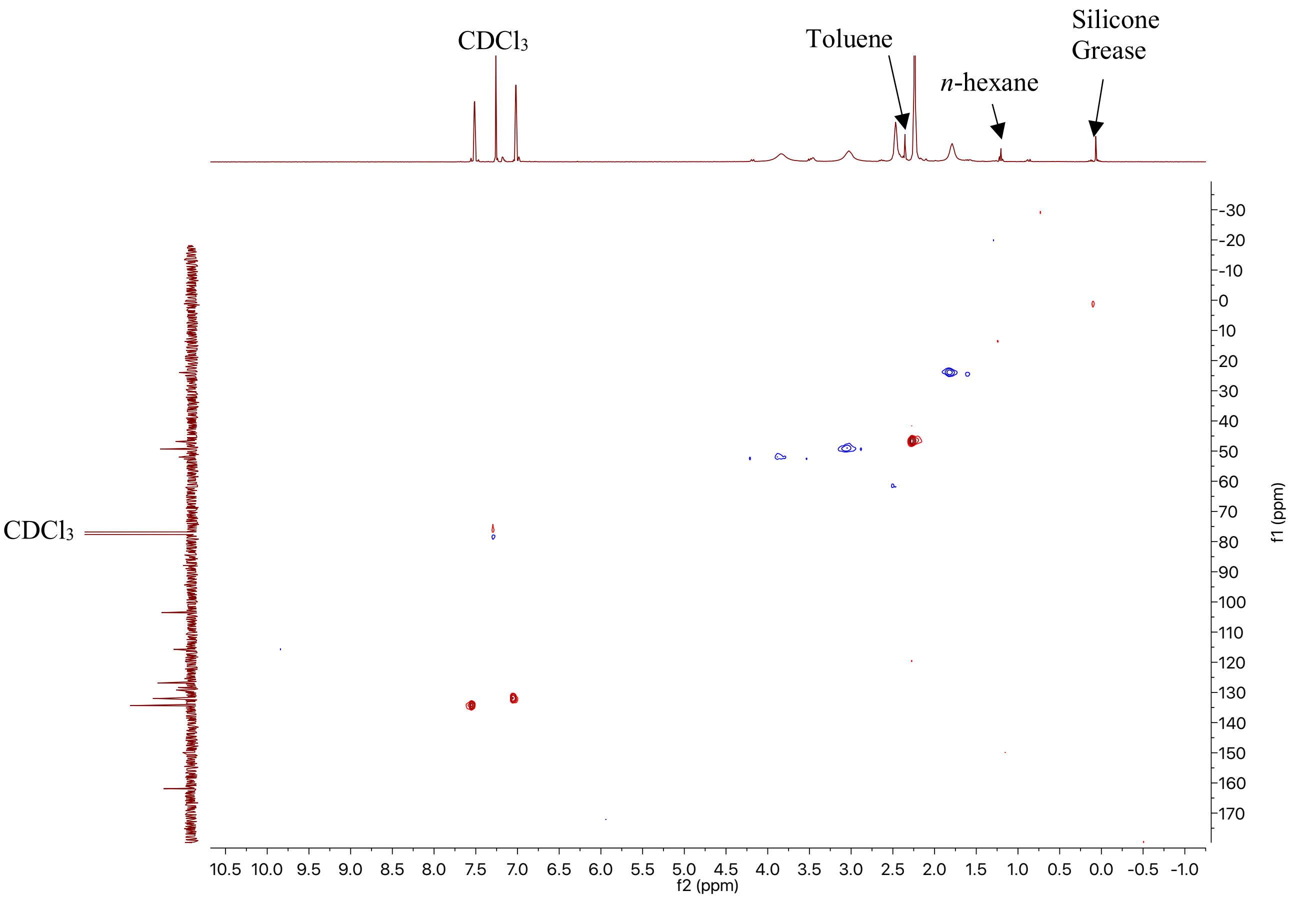

Figure S25. HSQC NMR ($CDCl_3$, 125 MHz) spectrum of Zn(**2**)$_2$.

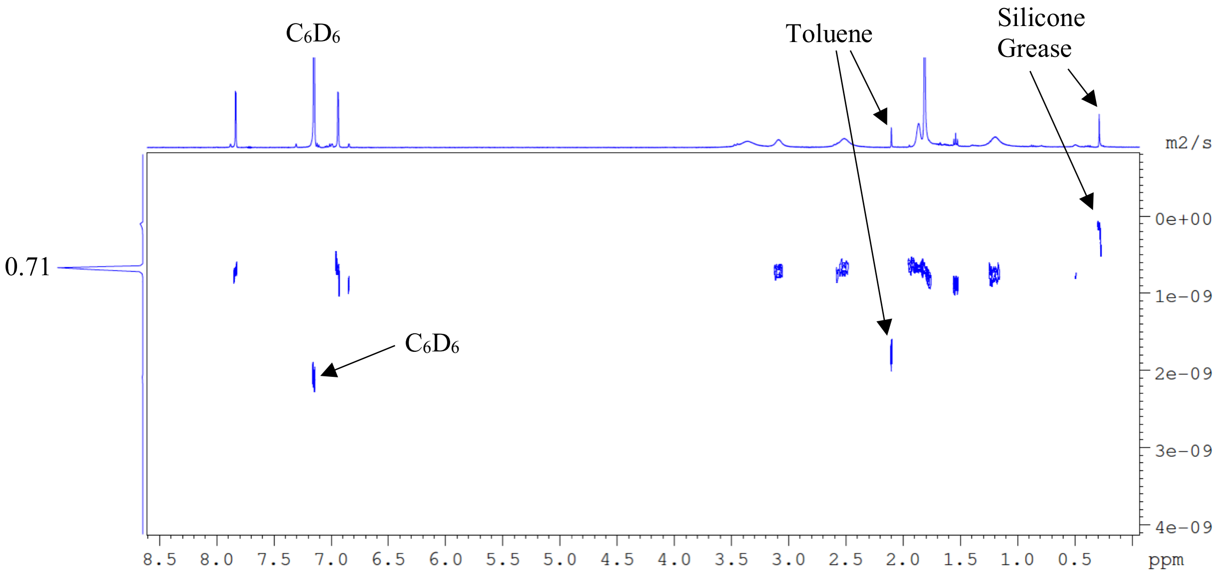

Figure S26. DOSY NMR (C_6D_6, 500 MHz) spectrum of Zn(**2**)$_2$, indicating only one species present in solution, with a diffusion constant of 0.71×10^{-9} m^2 s^{-1}.

Zn(**2**)Et:

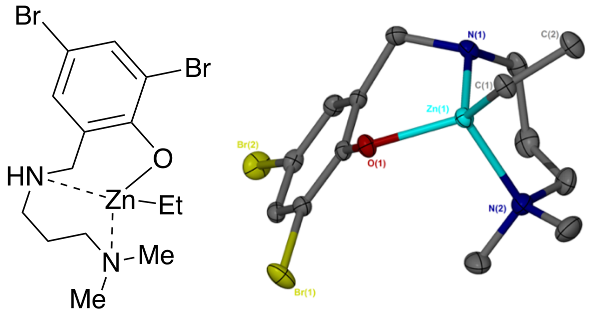

To a solution of **2**H (0.54 g, 1.5 mmol) in dry toluene (7.5 mL), $ZnEt_2$ (1.5 mL, 1.5 mmol, 1.0 M in hexane) was added dropwise with stirring, observing rapid precipitation of a white powder. The resulting suspension was stirred for 3 days at room temperature, after which a white solid was isolated by cannula filtration and dried *in vacuo* at 80 °C for 4 h.

^{1}H NMR (C_6D_6, 500 MHz): δ = 7.88 (s, 1H; ArH), 6.86 (s, 1H; ArH), 3.48 (d, J = 12 Hz, 1H; Ar-CH), 2.62 (d, J = 11 Hz, 1H; Ar-CH), 2.21 – 1.63 (br m, 9H; $N(CH_3)_2$, CH), 1.55 (t, J = 8 Hz, 4H; Zn-Et, CH), 1.44 – 1.37 (br m, 1H; CH), 0.85 (br s, 1H; NH), 0.52 (br s, 1H; CH), 0.45 – 0.33 (m, 1H; Zn-Et), 0.33 – 0.20 (m, 1H; Zn-Et). *N.B.* HSQC analysis assisted with -CH and -NH resonance assignment. -CH resonances were observed to be inequivalent and broad, indicative of catalyst asymmetry and structural fluxionality. Consequently, overlapping peaks between δ = 2.21 – 1.63 ppm were treated as single multiplet.

^{13}C{^{1}H} NMR (C_6D_6, 125 MHz): δ = 163.1, 135.4, 132.8, 124.2, 117.0, 102.7 (Ar), 61.0 (CH_2), 52.7 (Ar-CH_2), 47.5, 24.2 (CH_2), 14.2 (CH_3; Zn-Et), -3.5 (CH_2; Zn-Et). *N.B.* HSQC analysis assisted with peak assignment. Unaccounted for $N(CH_3)_2$ resonance, consistent with absence of well-defined resonance in ^{1}H NMR, indicating fluxionality at the ^{13}C{^{1}H} NMR time scale.

Yield = 0.54 g, 78%

Calculated for $C_{14}H_{22}Br_2N_2O_1Zn$: C, 36.59%; H, 4.83%; N, 6.10%. Found: C, 36.55%; H, 4.76%; N, 6.10%.

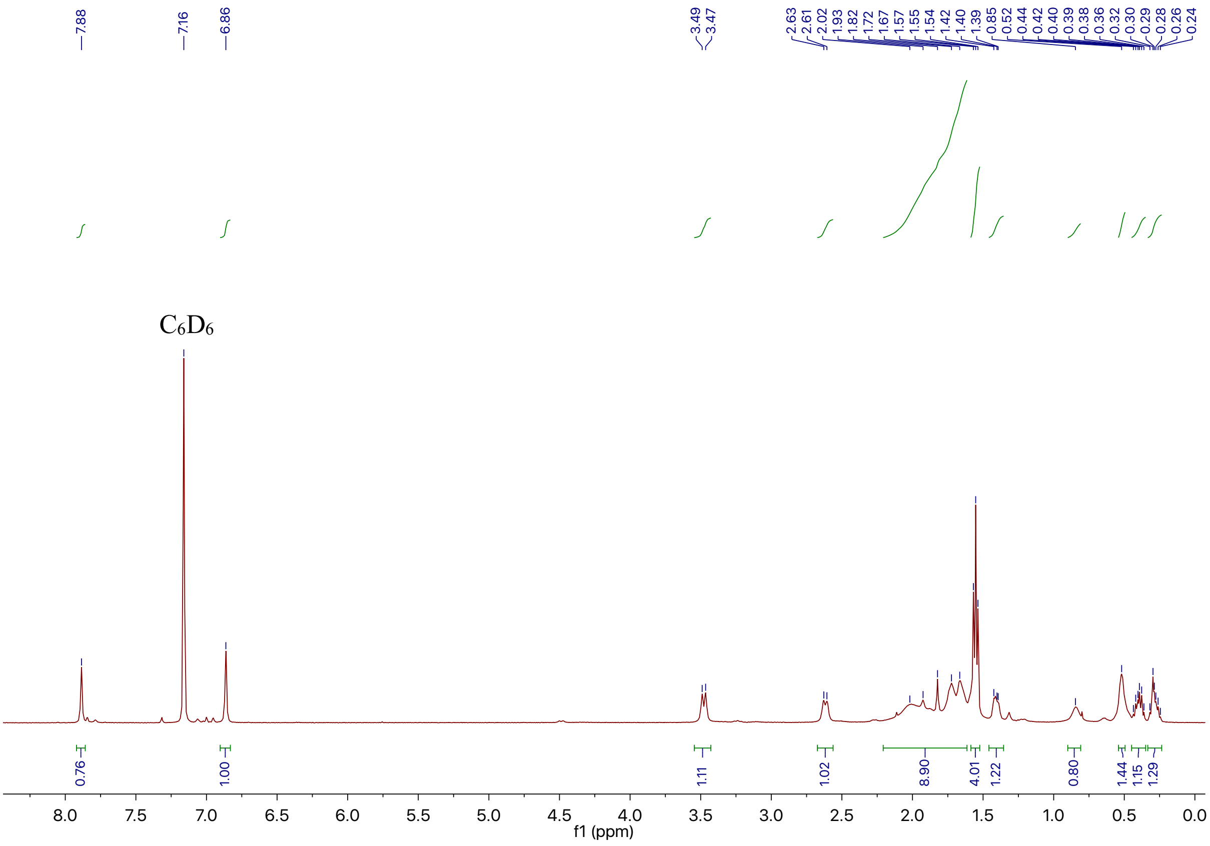

Figure S27. ^{1}H NMR (C_6D_6, 500 MHz) spectrum of Zn(**2**)Et.

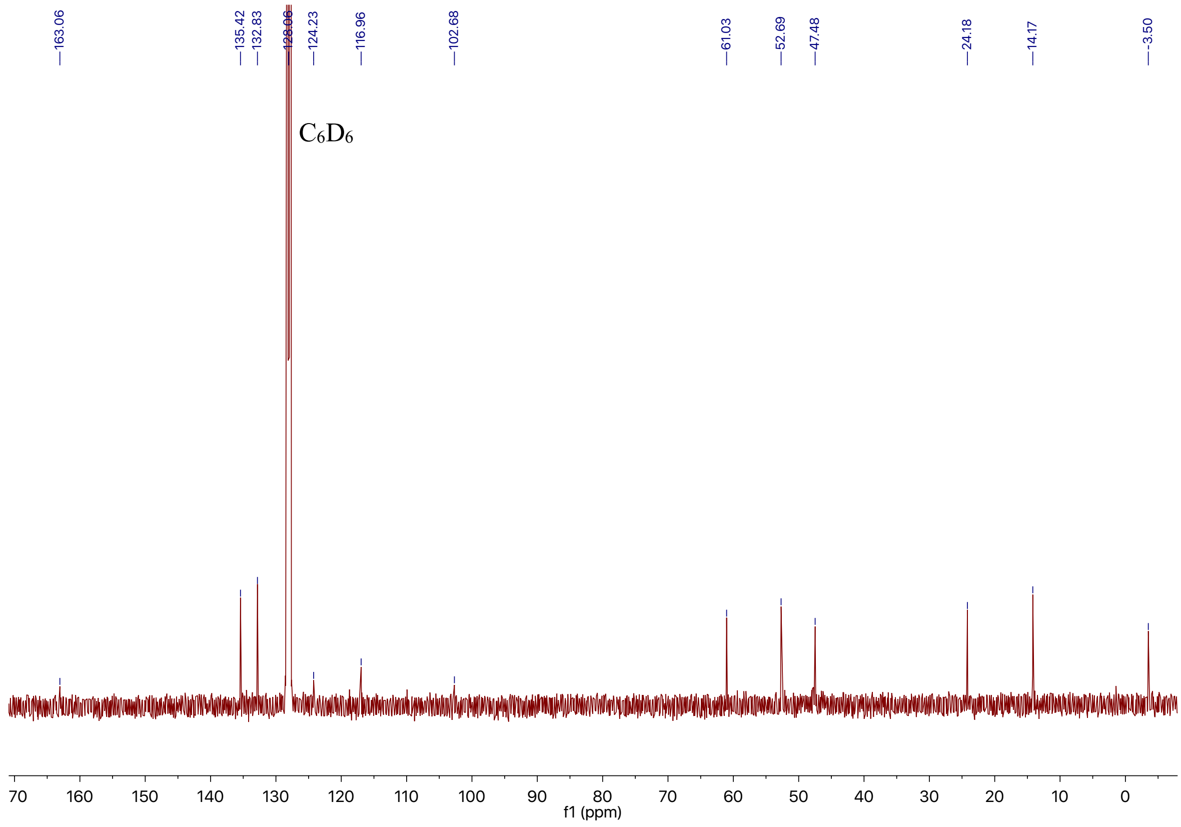

Figure S28. $^{13}C\{^{1}H\}$ NMR (C_6D_6, 125 MHz) spectrum of Zn(**2**)Et.

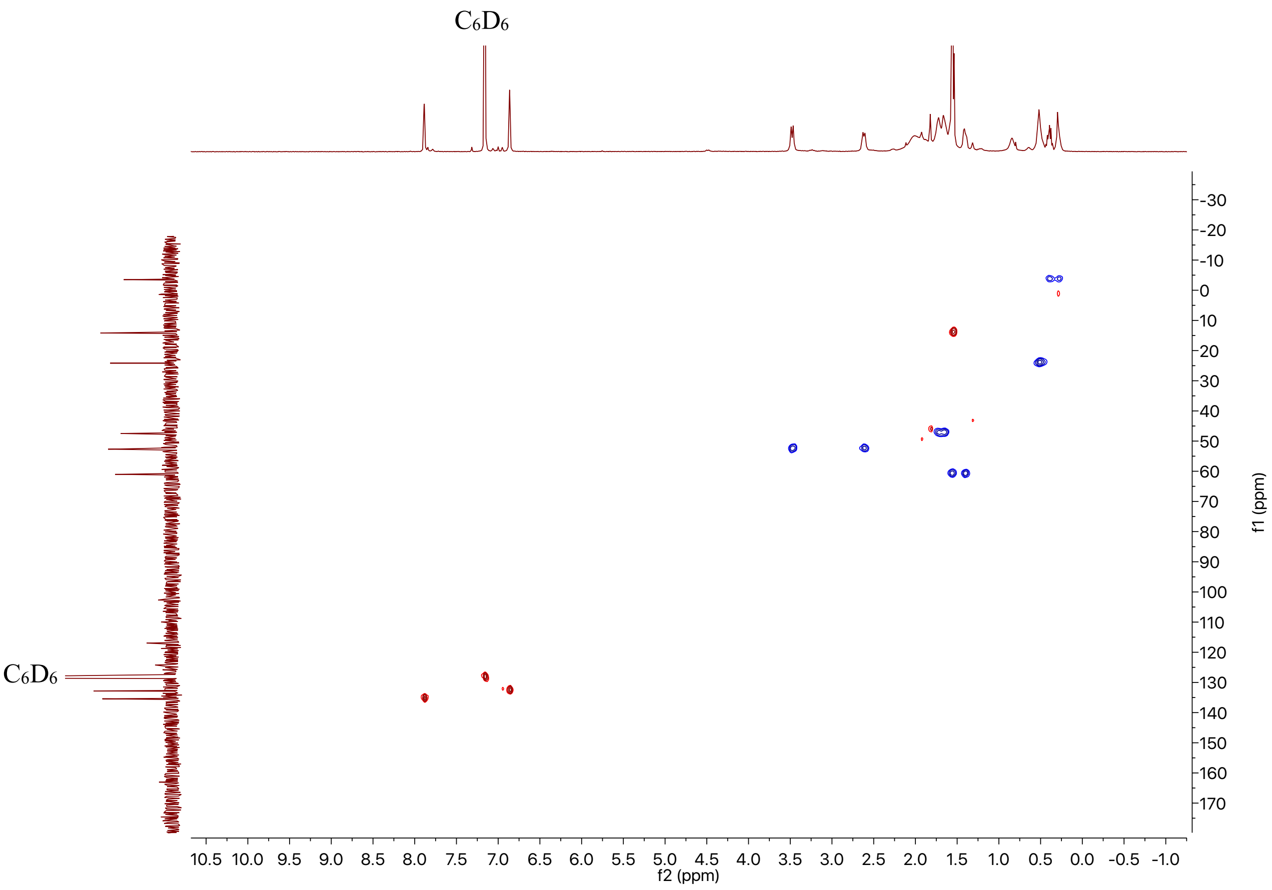

Figure S29. HSQC NMR (C_6D_6, 125 MHz) spectrum of Zn(**2**)Et.

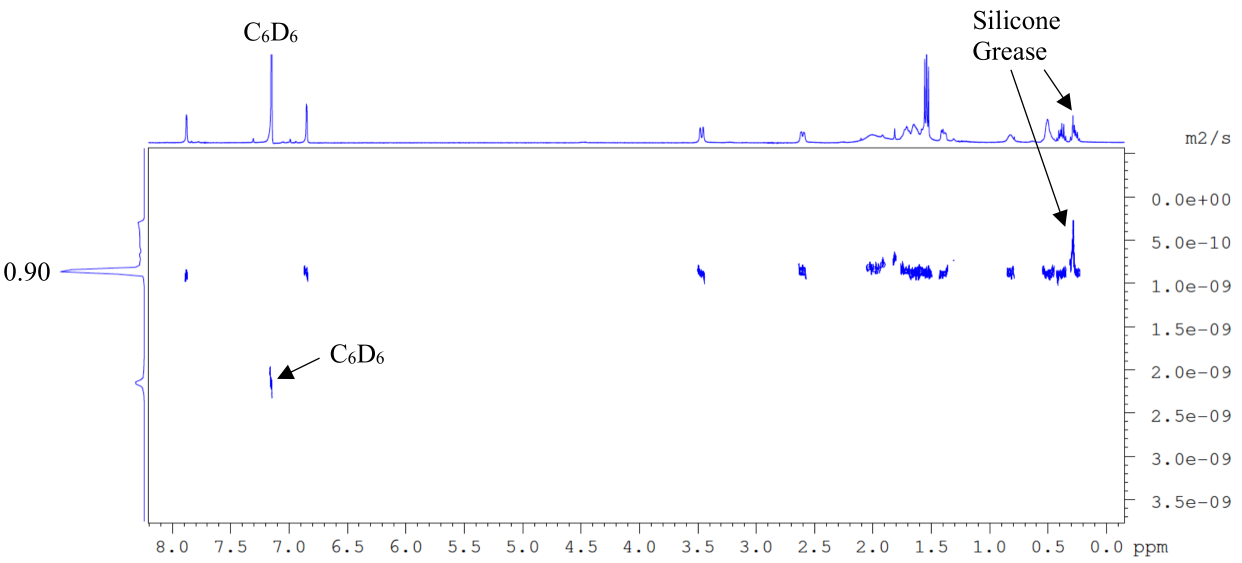

Figure S30. DOSY NMR (C_6D_6, 500 MHz) spectrum of Zn(**2**)Et, indicating only one species present in solution, with a diffusion constant of 0.90×10^{-9} m^2 s^{-1}.

Zn(**3**)$_2$:

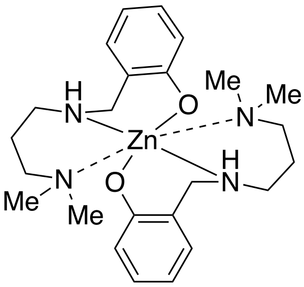

To a solution of **3**H (0.41g, 2 mmol) in dry toluene (10 mL), $ZnEt_2$ (1 mL, 1 mmol, 1.0 M in hexane) was added dropwise with stirring, observing rapid precipitation of a white powder. The suspension was stirred at 80 °C overnight under a static argon atmosphere. The solution was allowed to cool slowly to room temperature and left to stand for 1 h. A white powder was isolated by cannula filtration and dried *in vacuo* at 80 °C for 3 h.

^{1}H NMR ($CDCl_3$, 500 MHz): δ = 7.12 (td, J = 7, 2 Hz, 1H; ArH), 6.94 (d, J = 7 Hz, 1H; ArH), 6.75 (d, J = 8 Hz, 1H; ArH), 6.48 (t, J = 7 Hz, 1H; ArH), 3.92 (s, 2H; Ar-CH_2), 2.90 (t, J = 6 Hz, 2H; CH_2), 2.33 (t, J = 6 Hz, 2H; CH_2), 2.14 (s, 6H; $N(CH_3)_2$), 1.64 (p, J = 6 Hz, 2H; CH_2). *N.B.* HSQC analysis assisted with -CH and -NH peak assignment. Unaccounted for -NH resonance, likely due to rapid proton exchange in solution.

$^{13}C\{^1H\}$ NMR ($CDCl_3$, 125 MHz): δ = 166.9, 130.5, 130.3, 123.1, 120.2, 114.3 (Ar), 60.0 (CH_2), 53.2 (Ar-CH_2), 49.7 (CH_2), 46.3 ($N(CH_3)_2$), 24.6 (CH_2). *N.B.* HSQC analysis assisted with peak assignment.

Yield = 0.21 g, 44%

Calculated for $C_{24}H_{38}N_4O_2Zn$: C, 60.06%; H, 7.98%; N, 11.67%. Found: C, 59.96%; H, 7.84%; N, 11.60%.

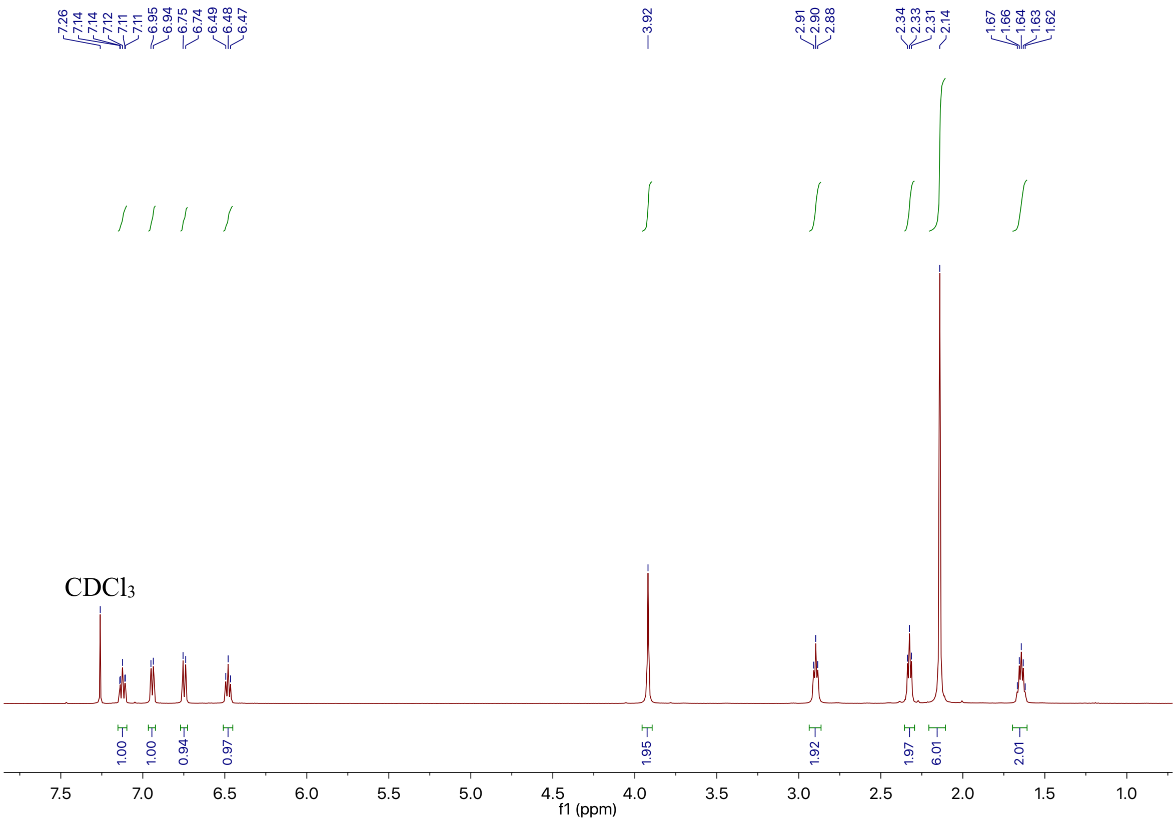

Figure S31. ^{1}H NMR ($CDCl_3$, 500 MHz) spectrum of Zn(**3**)$_2$.

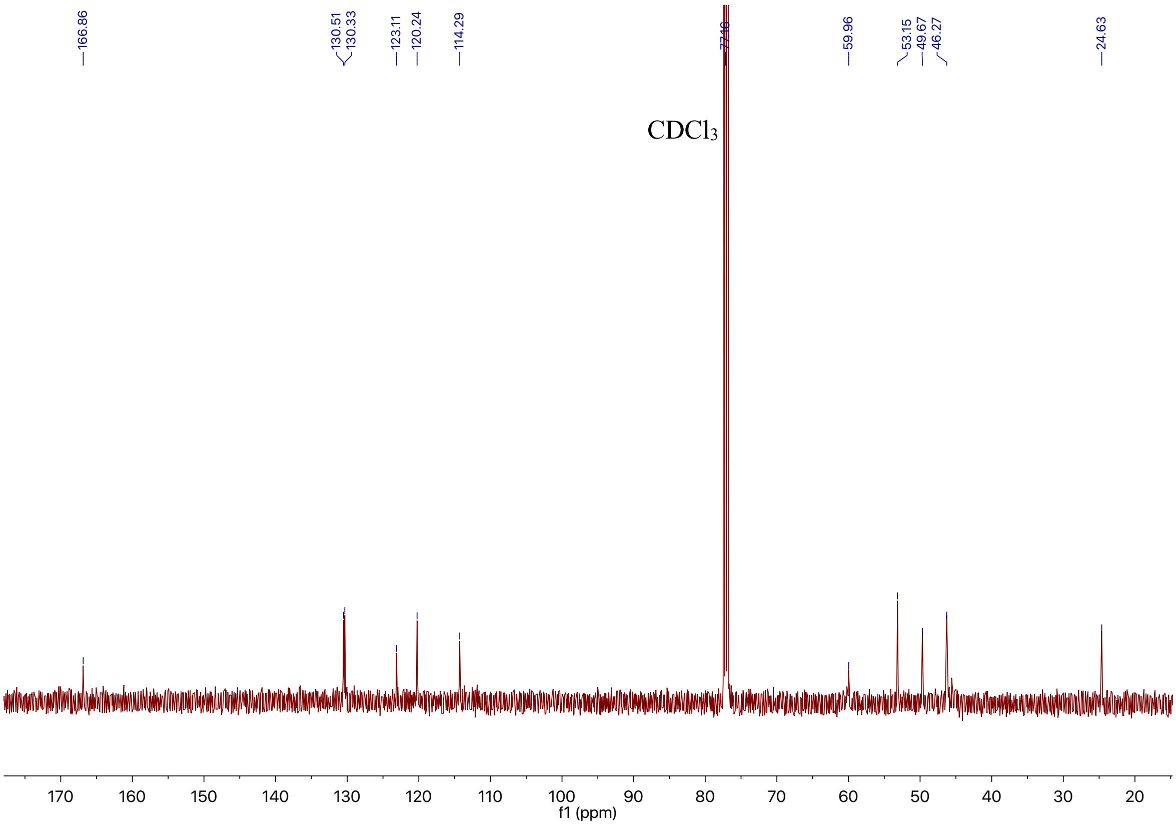

Figure S32. ^{13}C{^{1}H} NMR ($CDCl_3$, 125 MHz) spectrum of Zn(**3**)$_2$.

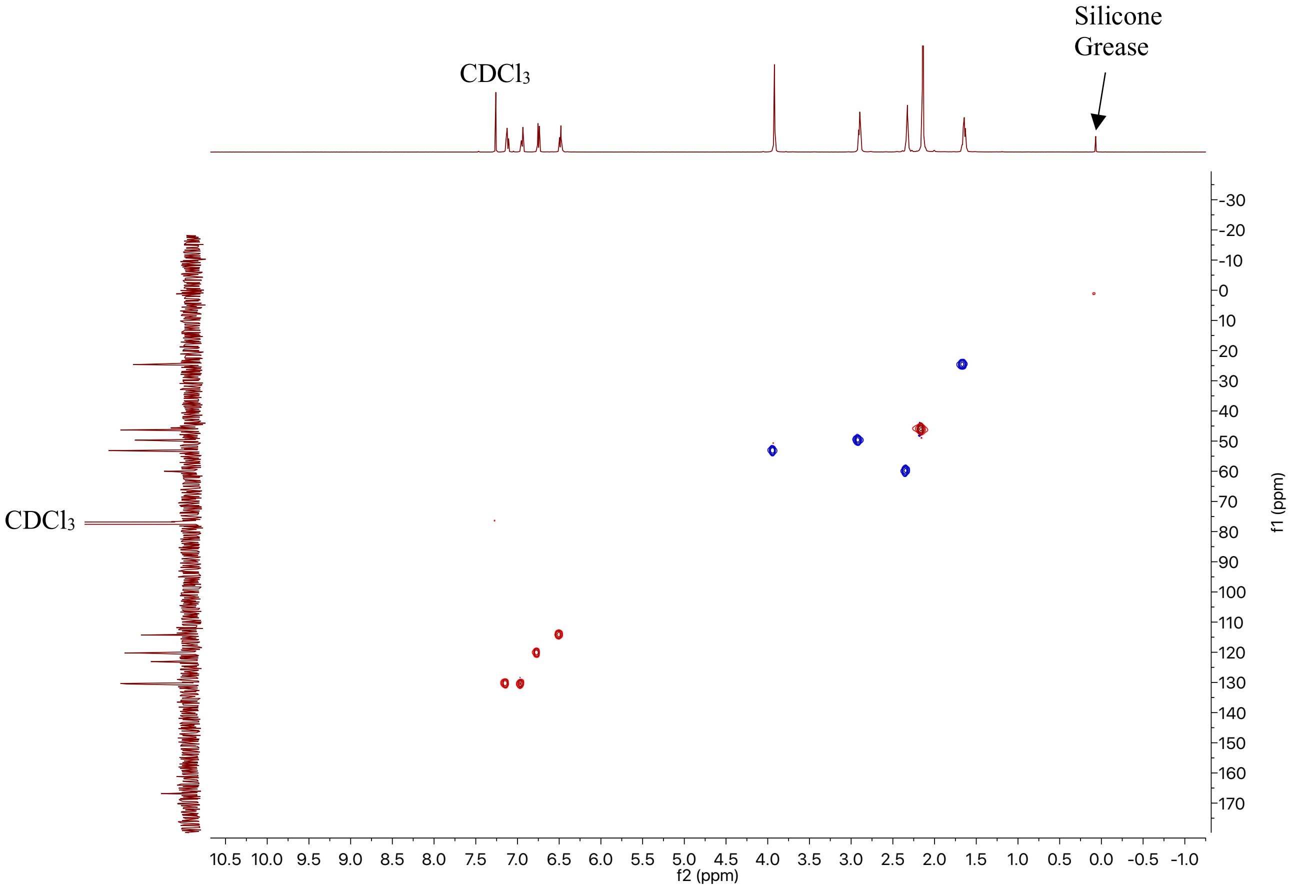

Figure S33. HSQC NMR ($CDCl_3$, 125 MHz) spectrum of Zn(**3**)$_2$.

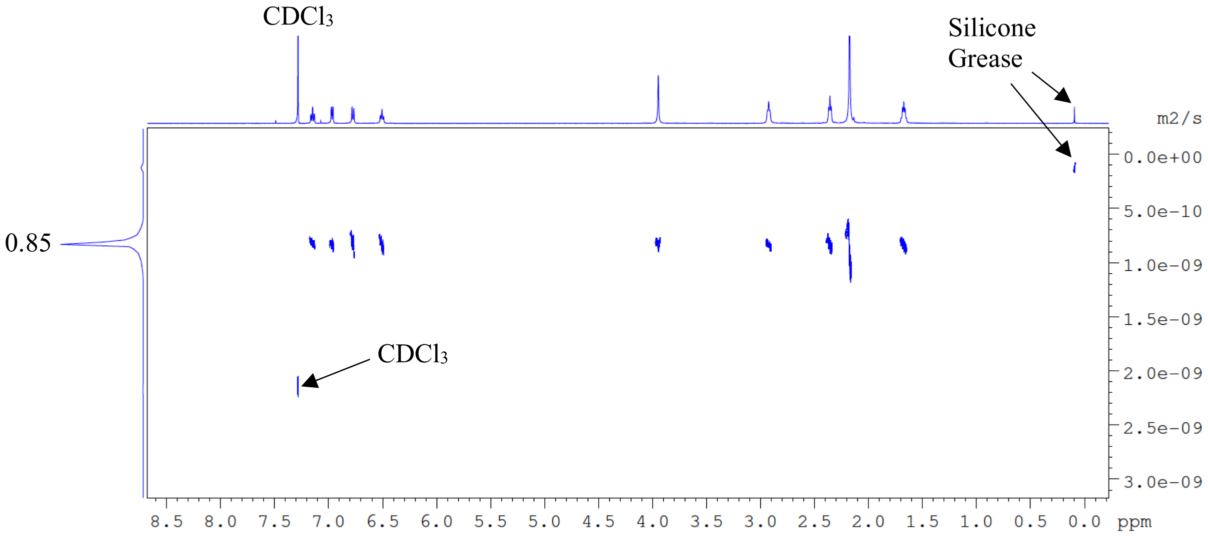

Figure S34. DOSY NMR ($CDCl_3$, 500 MHz) spectrum of Zn(**3**)$_2$, indicating only one species present in solution, with a diffusion constant of 0.85×10^{-9} m^2 s^{-1}.

[Zn(**3**)Et]$_2$:

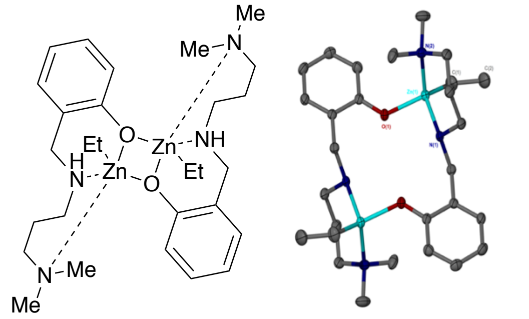

To a solution of **3**H (0.29 g, 1.4 mmol) in dry toluene (7 mL), $ZnEt_2$ (1.4 mL, 1.4 mmol, 1.0 M in hexane) was added dropwise with stirring, observing rapid precipitation of a white powder. The resulting suspension was stirred at room temperature overnight, noting the formation of a dark grey suspension. A clear colourless solution was isolated by hot cannula filtration and transferred to a freezer and left to stand for 1 h at -18 °C. An off-white solid was isolated by cannula filtration and dried *in vacuo* at 80 °C for 4 h.

VT NMR data:

^{1}H NMR (Toluene-d_8, 248 K, 500 MHz): δ = 7.30 (t, J = 8 Hz, 1H; ArH), 7.06 (d, J = 7 Hz, 1H; ArH), 6.95 (s, 1H; ArH), 6.71 (t, J = 7 Hz, 1H; ArH), 4.77 (d, J = 13 Hz, 1H; Ar-CH), 4.11 (t, J = 12 Hz, 1H; NH), 3.20 (t, J = 12 Hz, 1H; Ar-CH), 2.63 (d, J = 11 Hz, 1H; CH), 2.04 – 1.94 (m, 1H; CH), 1.86 (s, 3H; N(CH_3)), 1.84 (t, J = 8 Hz, 3H; Zn-Et), 1.70 – 1.63 (m, 1H; CH), 1.52 (s, 3H; N(CH_3)), 1.14 (d, J = 13 Hz, 1H; CH), 0.65 (q, J = 8 Hz, 2H; Zn-Et), 0.58 – 0.38 (m, 2H; CH). *N.B.* HSQC analysis assisted with -CH and -NH resonance assignment.

^{13}C{^{1}H} NMR (Toluene-d_8, 248 K, 125 MHz): δ = 167.9, 131.2, 130.1, 125.9, 119.3, 113.1 (Ar), 61.3 (CH_2), 53.9 (Ar-CH_2), 49.5 (N(CH_3)), 48.6 (CH_2), 42.8 (N(CH_3)), 24.5 (CH_2), 15.3 (CH_3: Zn-Et), -3.3 (CH_2; Zn-Et). *N.B.* HSQC analysis assisted with peak assignment.

Yield = 0.23 g, 27%

Calculated for $C_{28}H_{48}N_4O_2Zn_2$: C, 55.73%; H, 8.02%; N, 9.28%. Found: C, 54.68%; H, 7.82%; N, 9.08%.

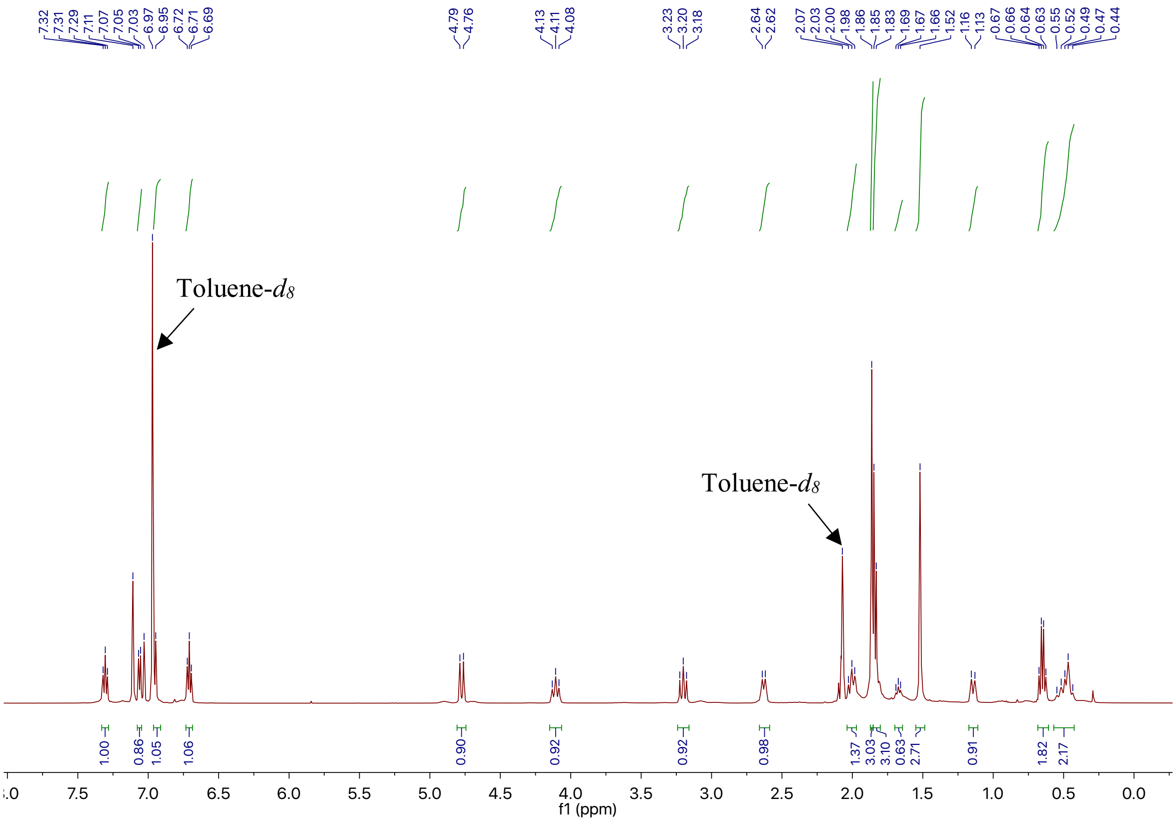

Figure S35. VT ^{1}H NMR (Toluene-d_8, 248 K, 500 MHz) spectrum of [Zn(**3**)Et]$_2$.

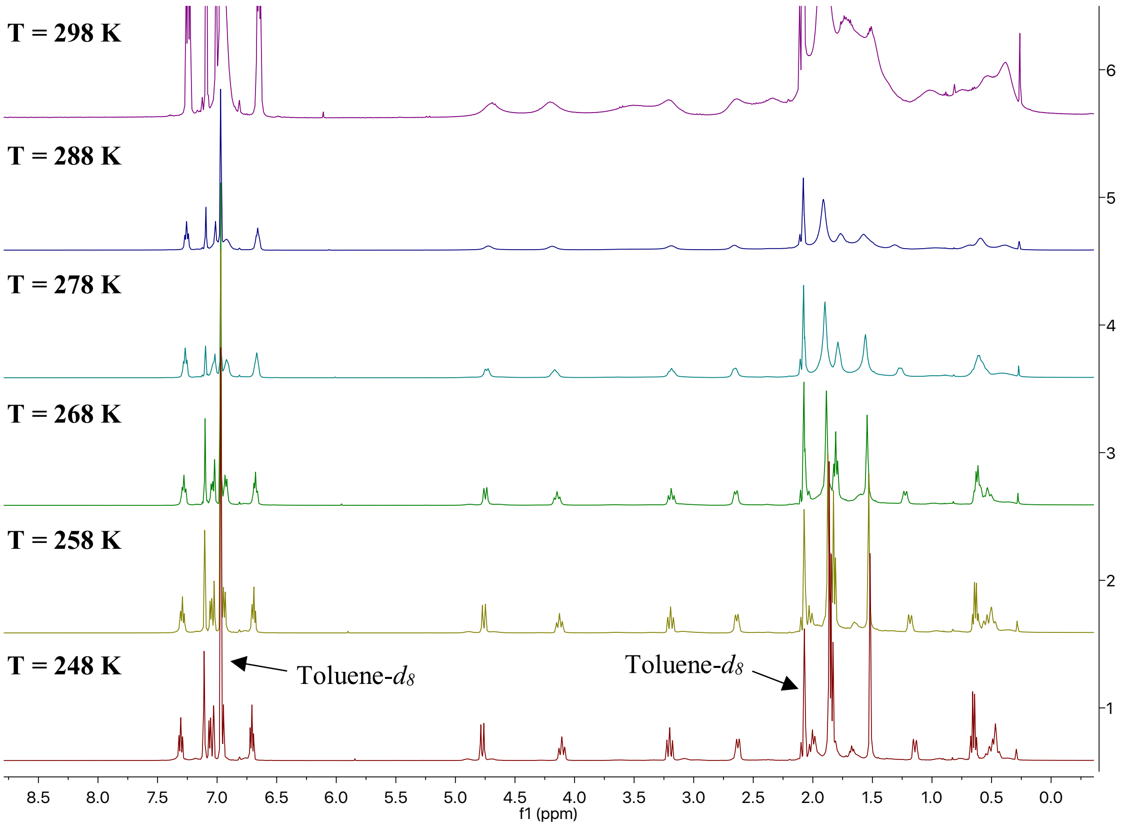

Figure S36. VT ^{1}H NMR (Toluene-d_8, 500 MHz) spectrum of [Zn(**3**)Et]$_2$ between 248 to 298 K.

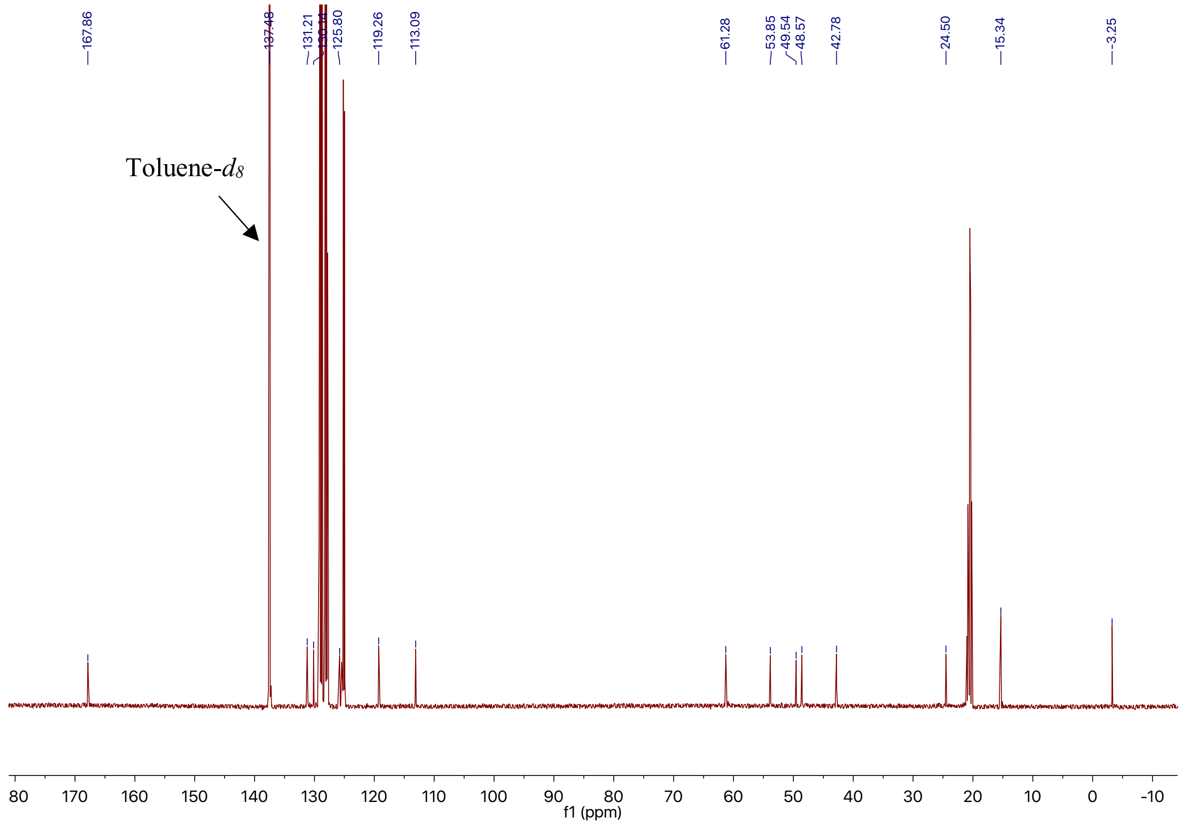

Figure S37. VT $^{13}C\{^{1}H\}$ NMR (Toluene-d_8, 248 K, 125 MHz) spectrum of $[Zn(\mathbf{3})Et]_2$.

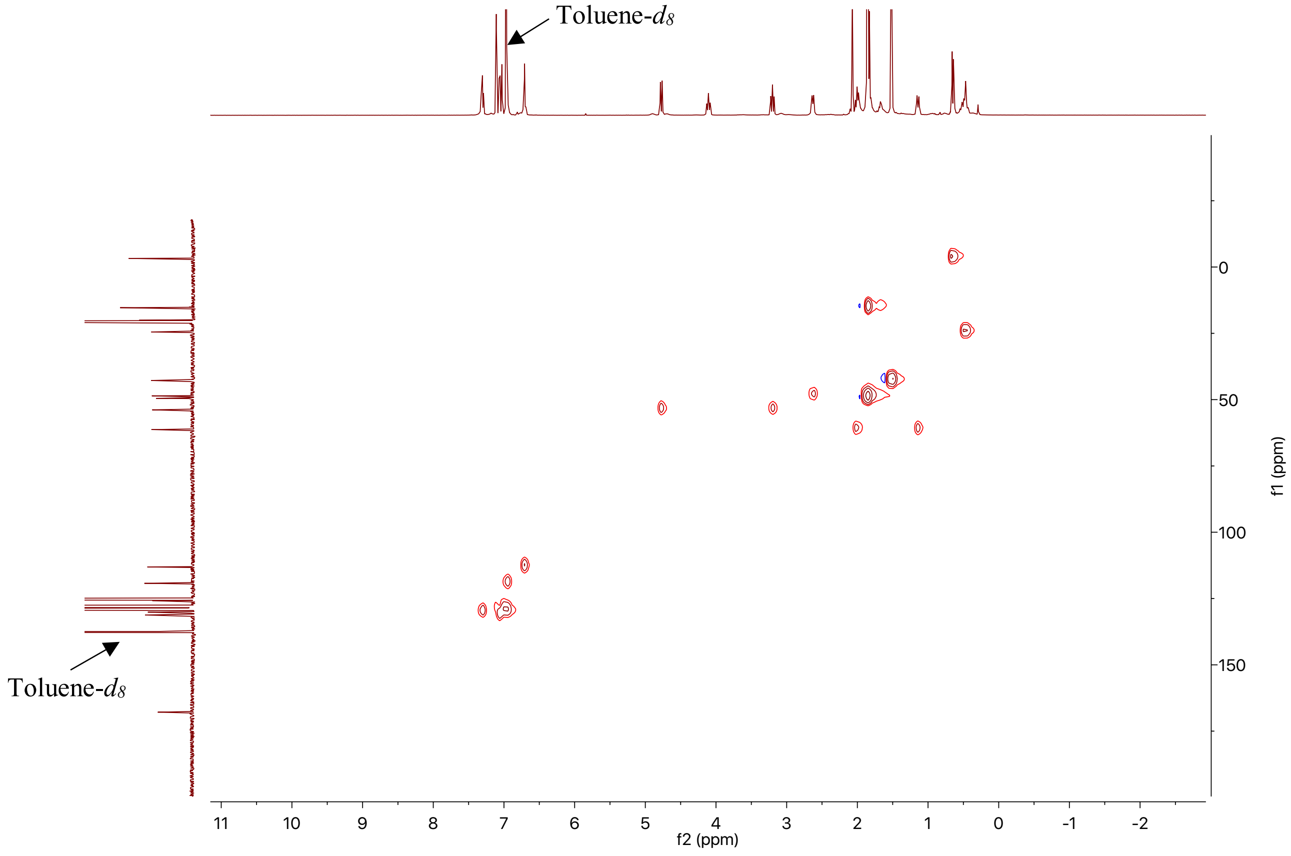

Figure S38. VT HSQC NMR (Toluene-d_8, 248 K, 125 MHz) spectrum of $[Zn(\mathbf{3})Et]_2$.

Zn(**4**)Et:

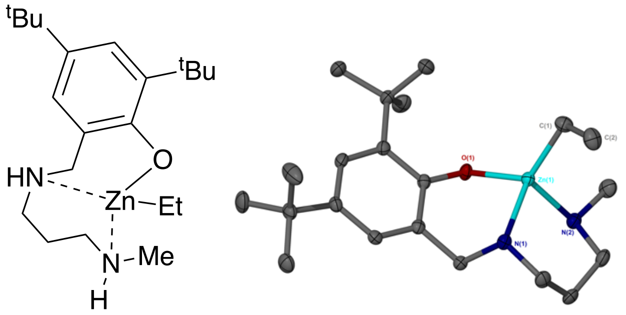

To a solution of **4**H (0.48 g, 1.56 mmol) in dry toluene (15.6 mL), $ZnEt_2$ (1.56 mL, 1.56 mmol) was added dropwise with stirring. The resulting solution was stirred at room temperature for 6 days, observing the formation of a dark grey suspension within 24 h. A clear pale-yellow solution was separated by cannula filtration and the solvent removed *in vacuo* to afford an off-white solid, which was redissolved in *n*-hexane (15 mL). The solution was concentrated to *ca.* 5 mL and placed in a freezer overnight. After *ca.* 18 h at – 18 °C, a white solid was isolated by cannula filtration and dried *in vacuo* at 80 °C for 4 h.

^{1}H NMR (C_6D_6, 500 MHz): δ = 7.64 (d, *J* = 3 Hz, 1H; ArH), 7.02 (d, *J* = 3 Hz, 1H; ArH), 3.32 (dd, *J* = 11, 8 Hz, 1H; Ar-CH), 3.20 (d, *J* = 10 Hz, 1H; Ar-CH), 2.07 – 1.99 (m, 2H; CH_2), 1.89 (s, 9H; $C(CH_3)_3$),1.86 (d, *J* = 6 Hz, 3H; $N(CH_3)H$), 1.79 – 1.70 (m, 1H; CH), 1.70 – 1.62 (m, 1H; CH), 1.55 (t, *J* = 8 Hz, 3H; Zn-Et), 1.52 (s, 9H; $C(CH_3)_3$), 1.30 – 1.25 (m, 1H; NH), 1.03 – 0.93 (m, 2H; CH, NH), 0.58 (d, *J* = 14 Hz, 1H; CH), 0.43 (qd, *J* = 8, 1 Hz, 2H; Zn-Et). *N.B.* HSQC analysis assisted with -CH and -NH resonance assignment. Evidence of peak broadening for -CH resonances, possibly indicating structural fluxionality.

$^{13}C\{^1H\}$ NMR (C_6D_6, 125 MHz): δ = 164.9, 138.6, 134.2, 125.0, 124.1 (Ar), 53.8 (Ar-CH_2), 52.2, 50.3 (CH_2), 37.3 ($N(CH_3)H$), 35.9, 34.3 ($C(CH_3)_3$), 32.5, 30.4 (CH_3), 25.5 (CH_2), 14.6 (CH_3; Zn-Et), -3.9 (CH_2; Zn-Et). *N.B.* HSQC analysis assisted with peak assignment. Unaccounted for Ar resonance likely due to peak overlapping with solvent. Additional peaks can be attributed to the presence of residual *n*-hexane.

Yield = 0.33 g, 53%

Calculated for $C_{21}H_{38}N_2O_1Zn$: C, 63.07%; H, 9.58%; N, 7.00%. Found: C, 62.52%; H, 9.58%; N, 6.94%.

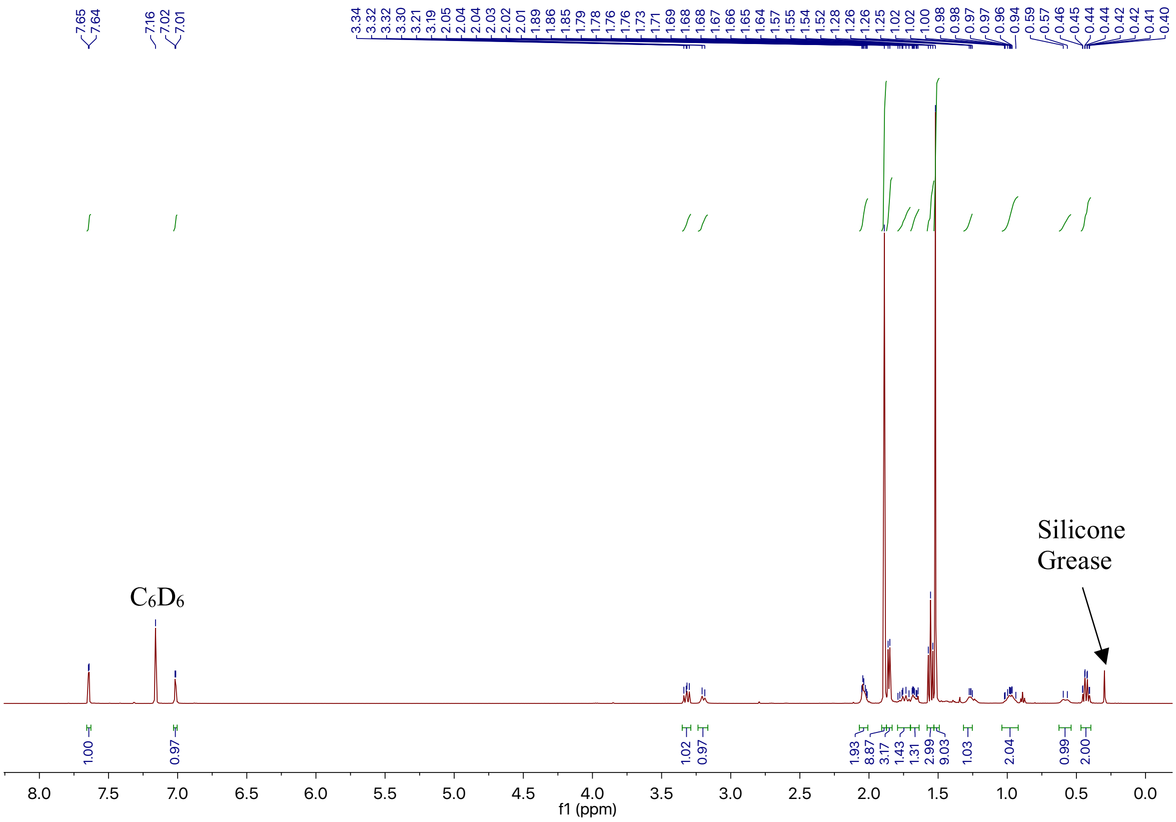

Figure S39. ^{1}H NMR (C_6D_6, 500 MHz) spectrum of Zn(**4**)Et.

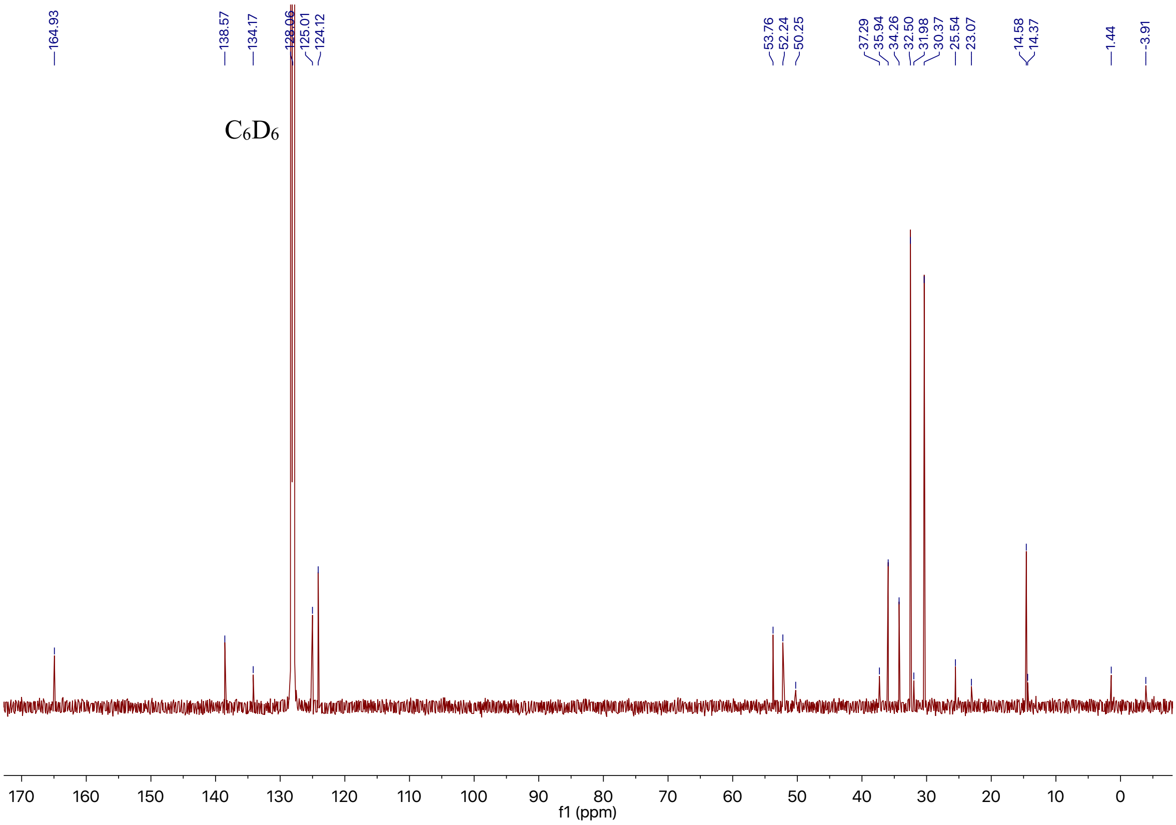

Figure S40. ^{13}C{^{1}H} NMR (C_6D_6, 125 MHz) spectrum of Zn(**4**)Et.

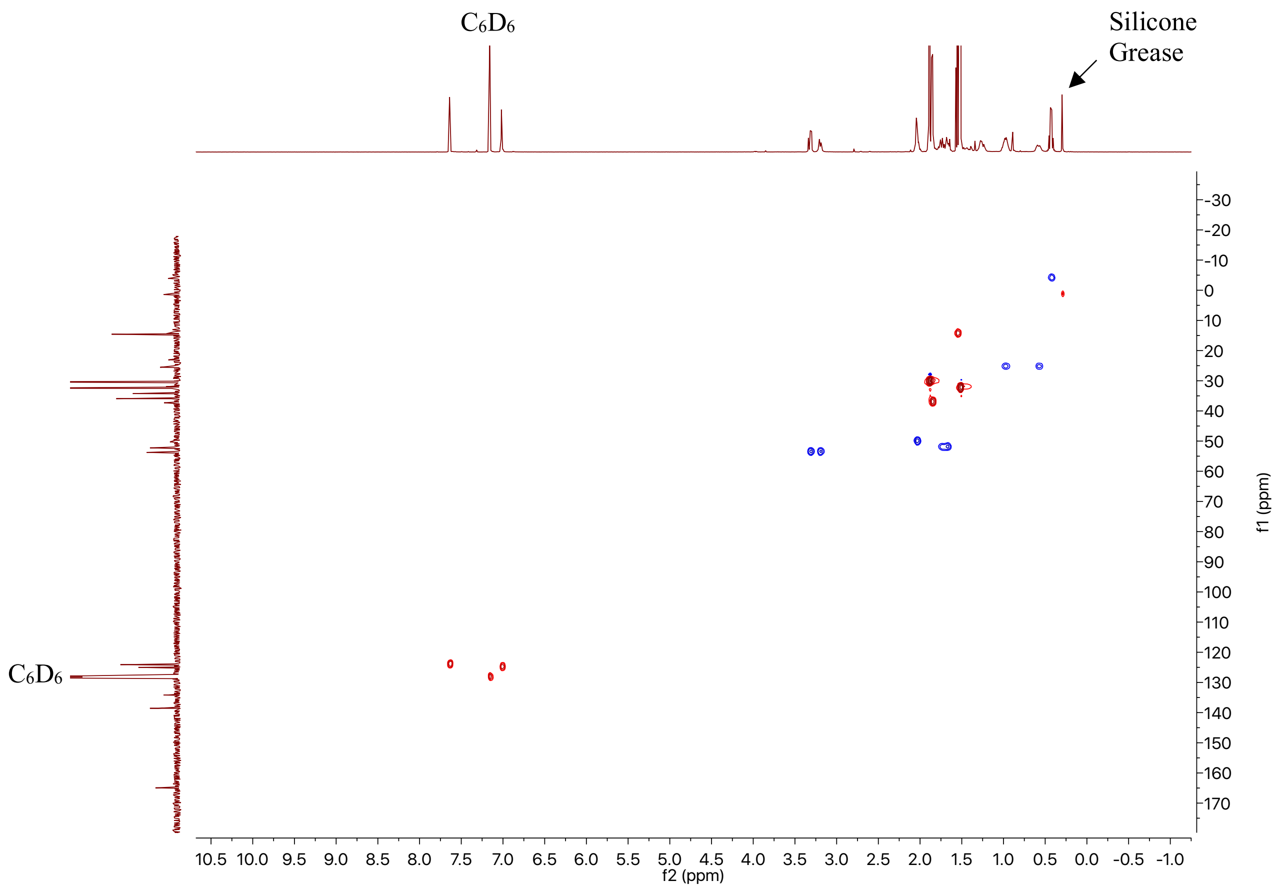

Figure S41. HSQC NMR (C_6D_6, 125 MHz) spectrum of Zn(**4**)Et.

Zn(**5**)Et:

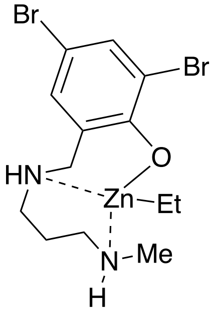

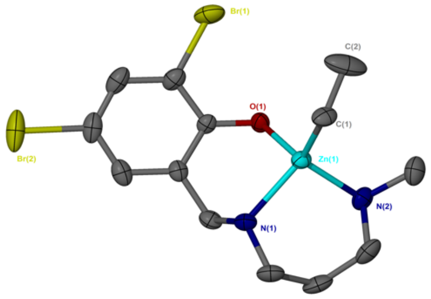

To a solution of **5**H (0.32 g, 0.9 mmol) in dry toluene (9 mL), $ZnEt_2$ (0.9 mL, 0.9 mmol) was added dropwise with stirring and the resulting solution stirred for 3 h at room temperature. The solvent was removed *in vacuo* to afford a white solid, which was washed in *n*-hexane (5 mL) overnight. A white powder was isolated by cannula filtration and dried *in vacuo* at 80 °C for 4 h.

^{1}H NMR (C_6D_6, 500 MHz): δ = 7.81 (d, *J* = 3 Hz, 1H; ArH), 6.82 (d, *J* = 2 Hz, 1H; ArH), 3.11 (br s, 1H; Ar-CH), 2.35 (br s, 2H; Ar-CH, CH), 2.17 (d, *J* = 6 Hz, 3H; N(CH_3)H), 1.91 – 1.73 (m, 3H; CH, NH), 1.64 (d, *J* = 12 Hz, 1H; CH), 1.55 (t, *J* = 8 Hz, 3H; Zn-Et), 0.89 – 0.78 (m, 2H; CH, NH), 0.63 (d, *J* = 15 Hz, 1H; CH), 0.43 – 0.31 (m, 2H; Zn-Et). *N.B.* HSQC analysis assisted with -CH and -NH peak assignment. Evidence of peak broadening for -CH resonances, possibly indicating structural fluxionality.

^{13}C{^{1}H} NMR (C_6D_6, 125 MHz): δ = 162.2, 135.5, 135.3, 132.3, 116.7, 104.0 (Ar), 52.8 (CH_2), 50.9 (Ar-CH_2), 49.9 (CH_2), 38.0 (N(CH_3)H), 25.2 (CH_2), 14.1 (CH_3; Zn-Et), -4.1 (CH_2; Zn-Et). *N.B.* HSQC analysis assisted with peak assignment.

Yield = 0.25 g, 62%

Calculated for $C_{13}H_{20}Br_2N_2O_1Zn$: C, 35.05%; H, 4.53%; N, 6.29%. Found: C, 33.36%; H, 4.22%; N, 5.70%. Elemental analysis more consistent with a degree of hydroxide during analysis, indicating poor hydrolytic stability. Zn(**5**)OH, theoretical: C, 30.48%; H, 3.72%; N, 6.46%

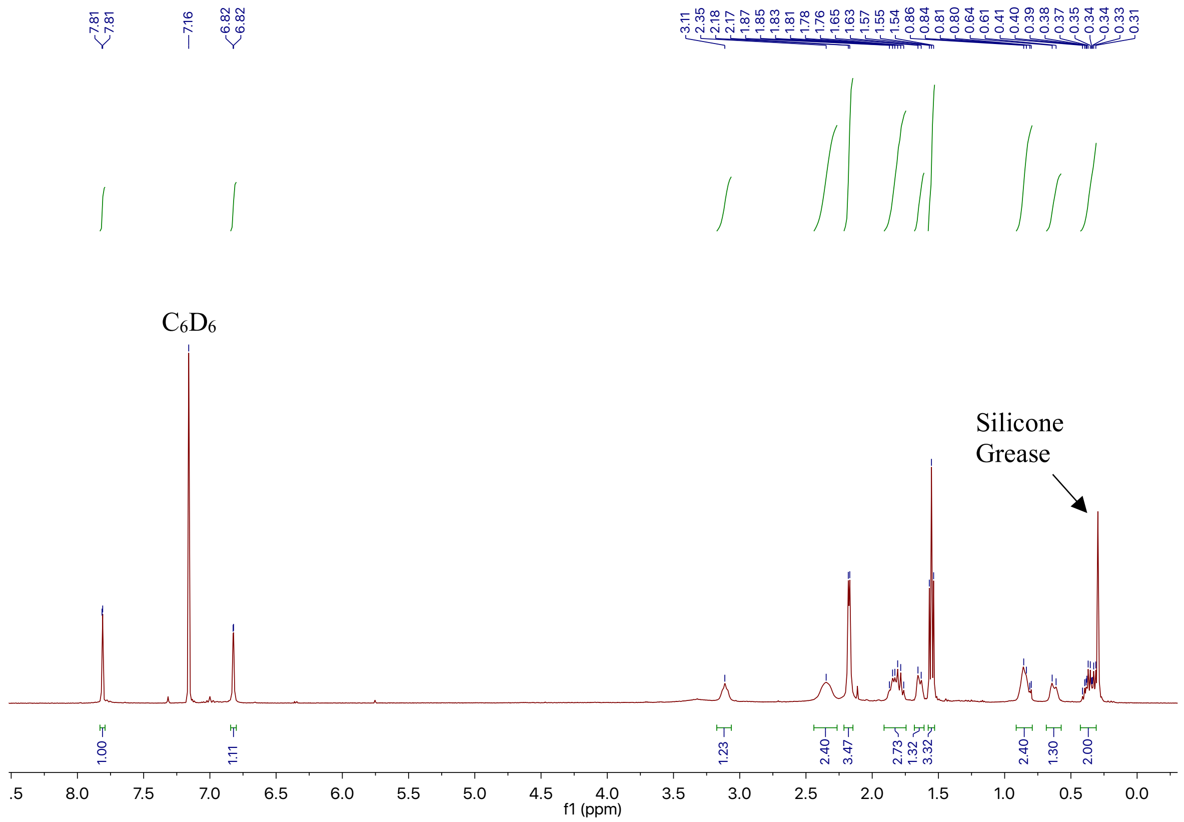

Figure S42. ^{1}H NMR (C_6D_6, 500 MHz) spectrum of Zn(**5**)Et.

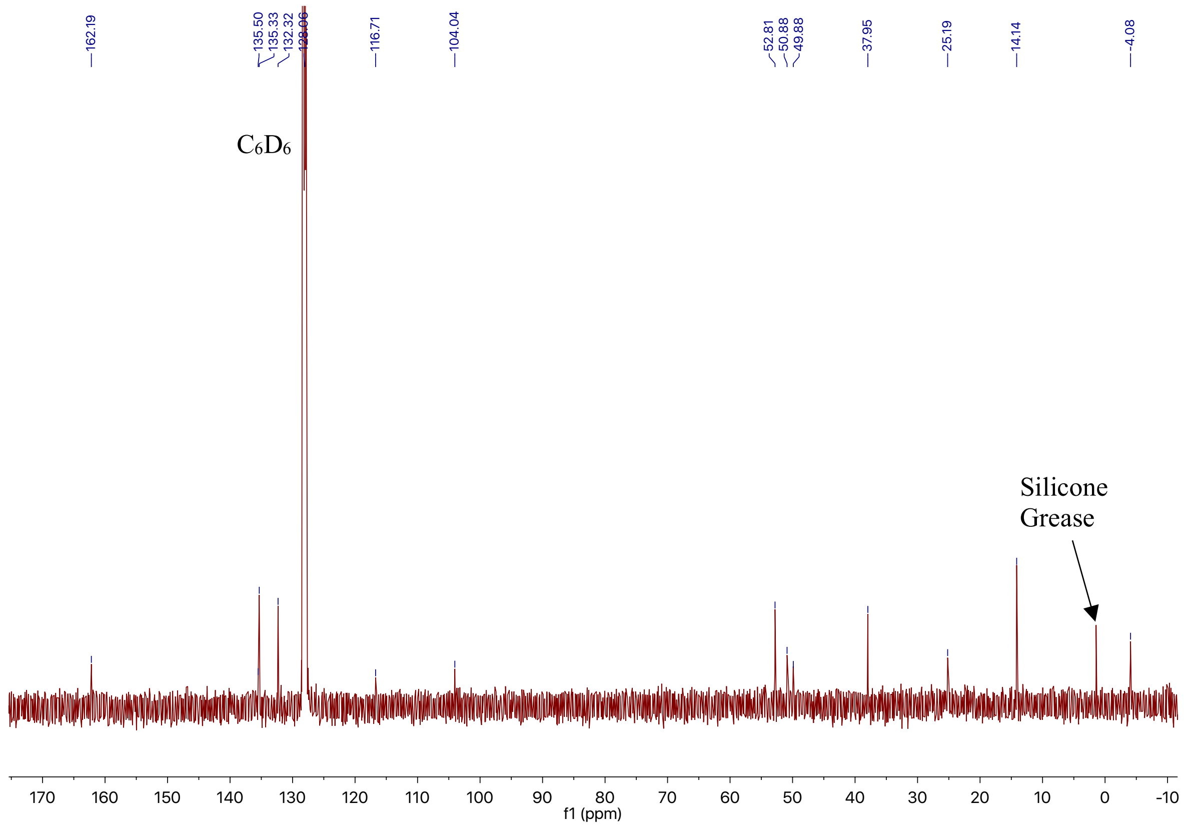

Figure S43. $^{13}C\{^{1}H\}$ NMR (C_6D_6, 125 MHz) spectrum of Zn(**5**)Et.

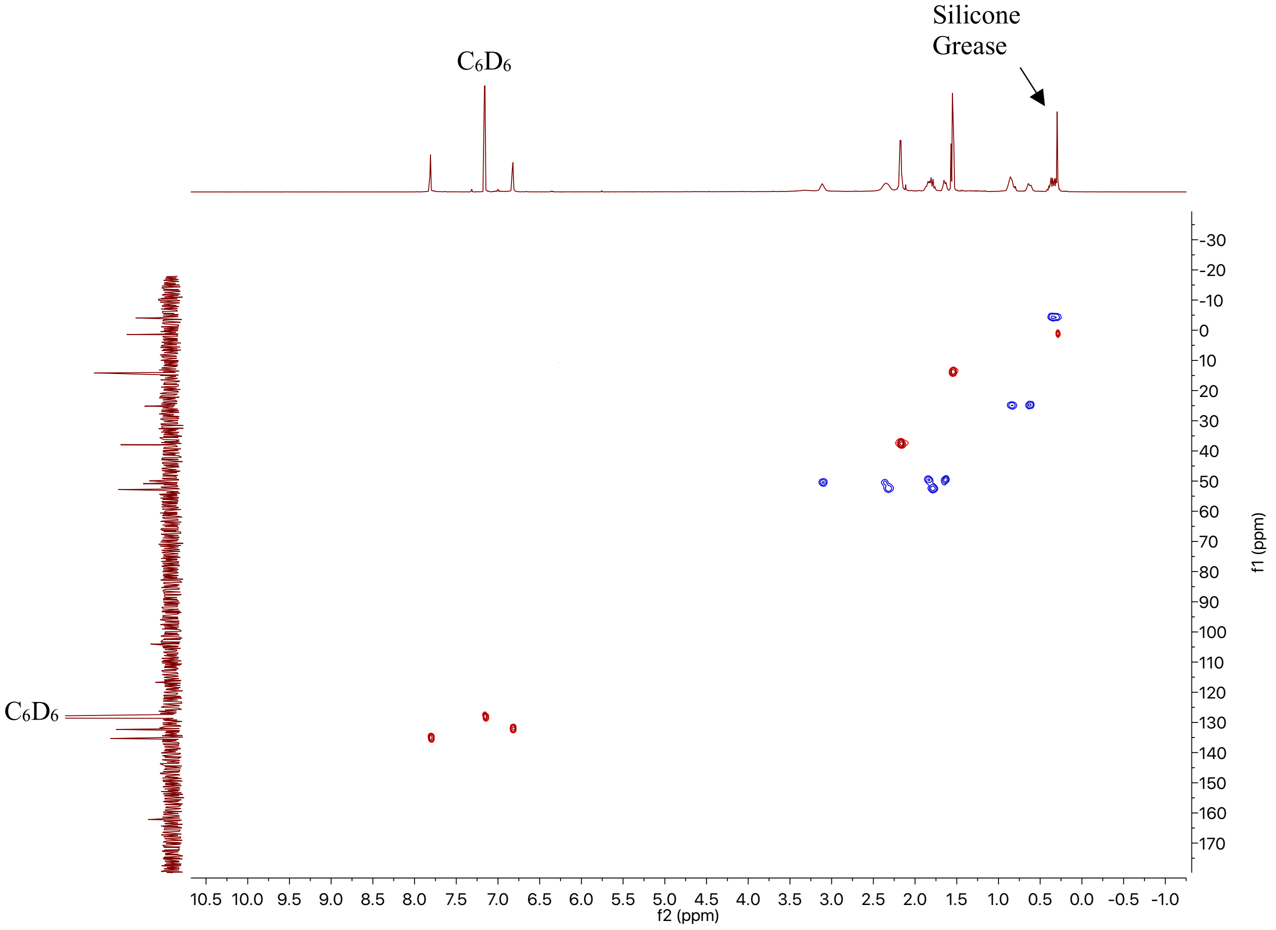

Figure S44. HSQC NMR (C_6D_6, 125 MHz) spectrum of Zn(**5**)Et.

2.3. Catalyst Stability Testing

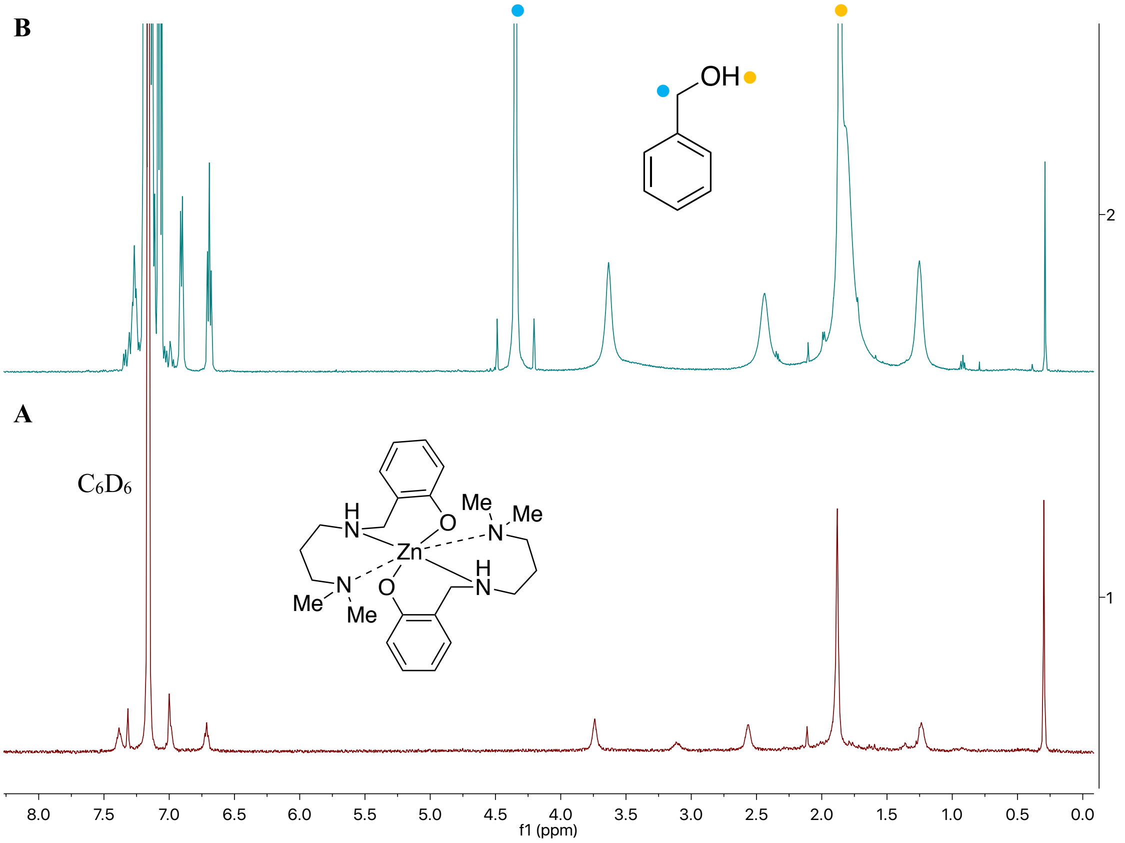

Figure S45. Stacked ^{1}H NMR (C_6D_6, 500 MHz) spectra assessing the stability of Zn(**3**)$_2$ with excess BnOH at room temperature: (**A**) Zn(**3**)$_2$, and; (**B**): Zn(**3**)$_2$ + BnOH. *N.B.* Zn(**3**)$_2$ exhibited greater structural fluxionality in C_6D_6 relative to $CDCl_3$, evidenced by peak broadening, although the structure remains unambiguous.

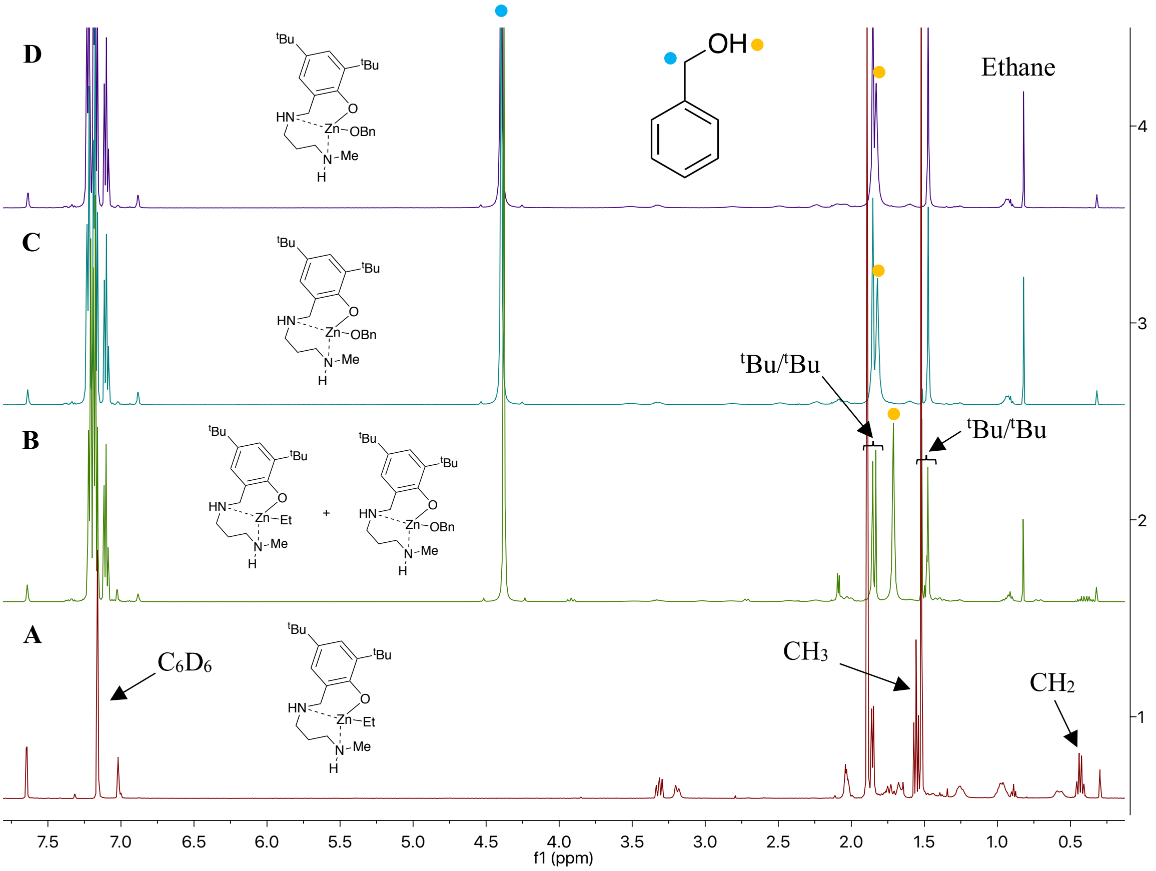

Figure S46. Stacked ^{1}H NMR (C_6D_6, 500 MHz) spectra assessing the stability of Zn(**4**)Et with excess BnOH at room temperature: (**A**) Zn(**4**)Et, (**B**) Zn(**4**)Et + BnOH after 15 mins, (**C**) Zn(**4**)Et + BnOH after 1 h, and; (**D**) Zn(**4**)Et + BnOH after 2 h. *N.B.* In the absence of *rac*-lactide, it remains unknown whether Zn(**4**)OBn adopts a monomeric or dimeric structure in solution. However, during ring-opening polymerization (ROP) the rate of propagation (k_p) >> rate of initiation (k_i), thus Zn(**4**)OBn is depicted as monomeric since the active species is likely consumed before a rearrangement to the dimeric counterpart ([Zn(**4**)OBn]$_2$) is possible. Dissolved ethane resonance appears at δ = 0.82 ppm, consistent with the literature.[1]

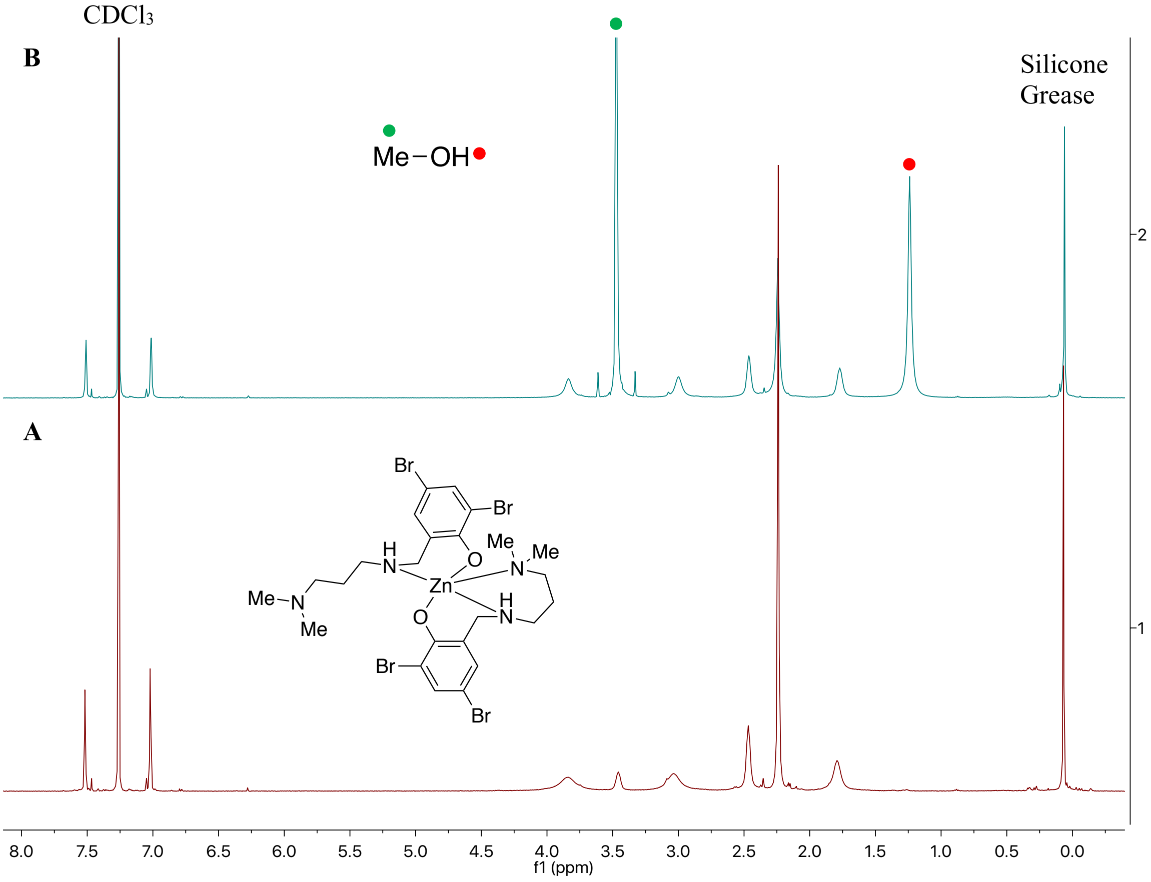

Figure S47. Stacked ^{1}H NMR ($CDCl_3$, 500 MHz) spectra assessing the stability of Zn(**2**)$_2$ with MeOH at room temperature: (**A**) Zn(**2**)$_2$, and; (**B**): Zn(**2**)$_2$ + MeOH.

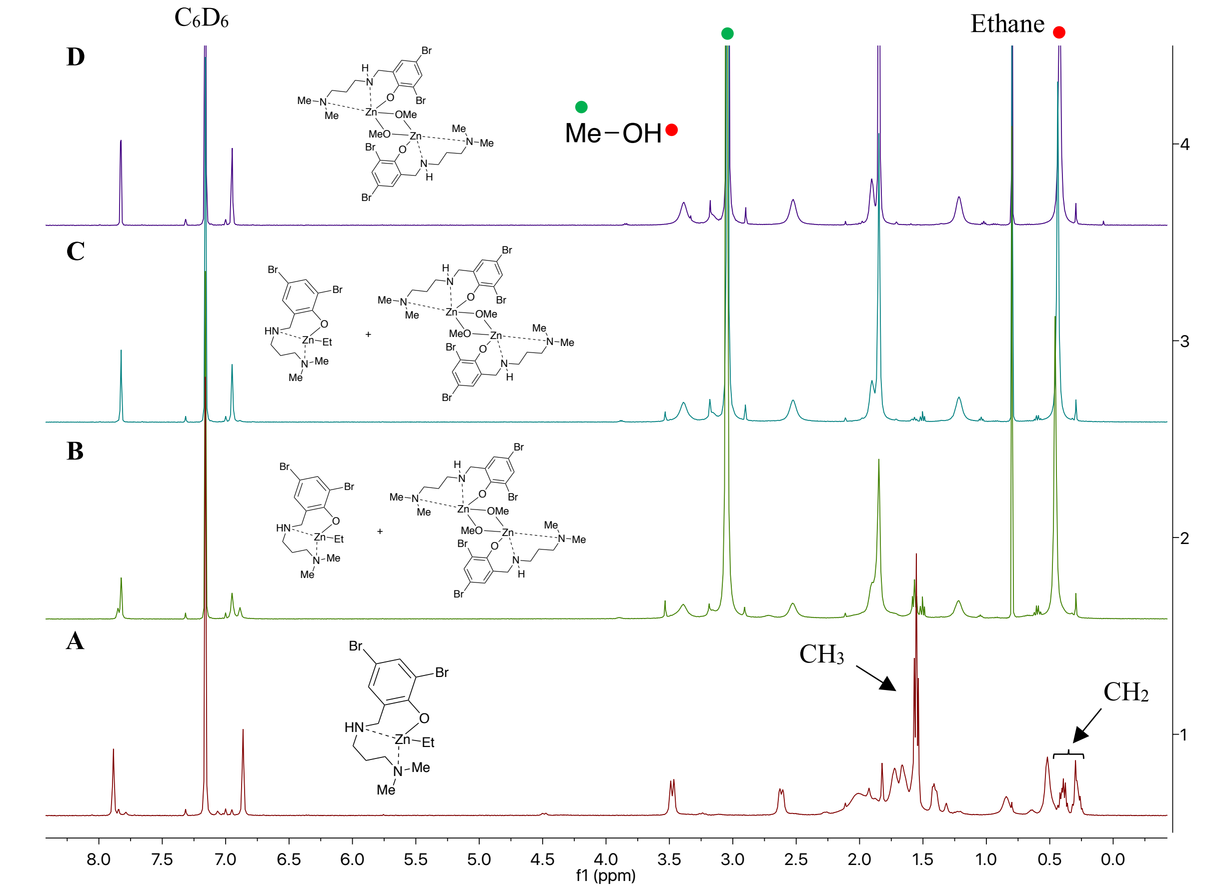

Figure S48. Stacked ^{1}H NMR (C_6D_6, 500 MHz) spectra assessing the stability of Zn(**2**)Et with excess MeOH at room temperature: (**A**) Zn(**2**)Et, (**B**) Zn(**2**)Et + MeOH after 15 mins, (**C**) Zn(**2**)Et + MeOH after 30 mins, and; (**D**) Zn(**2**)Et + MeOH after 1 h. *N.B.* Zn(**2**)OMe is shown to adopt a dimeric structure based on DOSY analysis. Dissolved ethane resonance appears at δ = 0.80 ppm, consistent with the literature.[1]

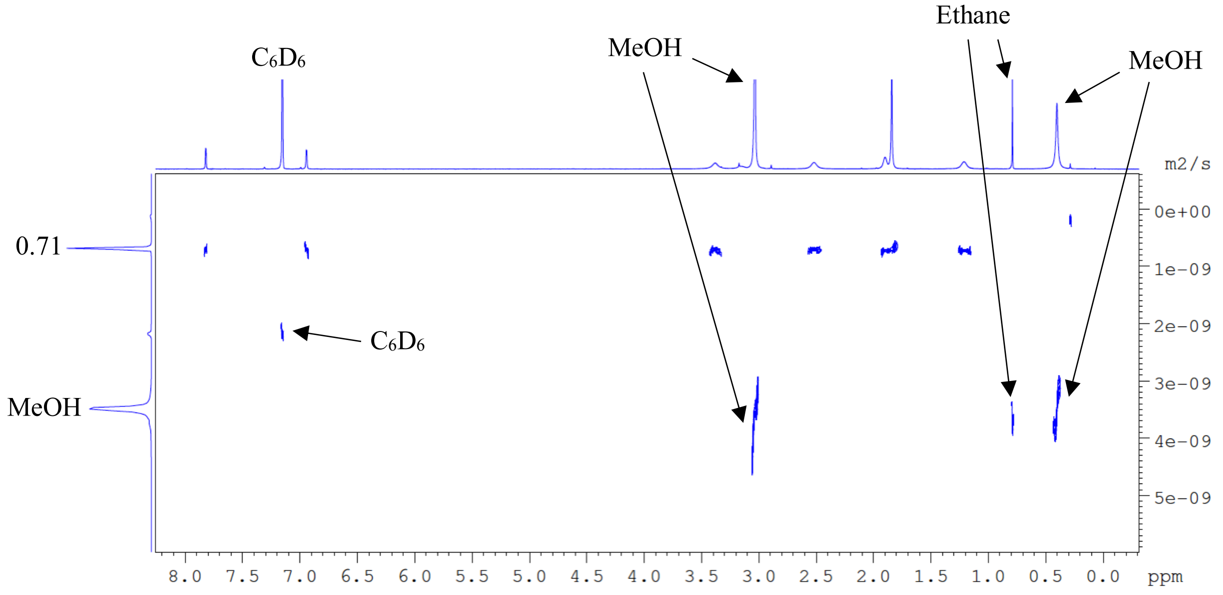

Figure S49. DOSY NMR (C_6D_6, 500 MHz) spectrum of Zn(**2**)OMe, indicating only one species present in solution, with a diffusion constant of 0.71×10^{-9} m^2 s^{-1}.

3. Lactide Polymerization

3.1. Polymerization of *rac*-LA

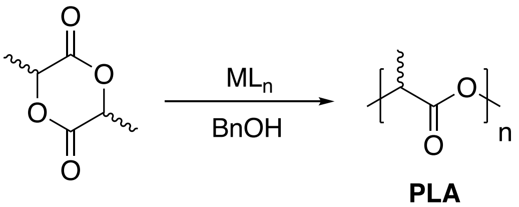

Scheme S2. Metal-mediated ring-opening polymerization (ROP) of *rac*-LA.

General procedure: polymerizations were conducted in a J Young's ampoule under argon. All melt polymerizations were performed in the absence of solvent. Melt polymerizations were performed with *rac*-LA (3.0 g, 21 mmol) to which the required amount of metal complex and benzyl alcohol (BnOH) co-initiator were loaded in a glovebox filled with argon (7.15 µL, 69 µmol) {[*rac*-LA]:[M]:[BnOH] = 10000:1:33}. The ampoule was then submerged in a preheated oil bath (130 °C) and the polymerization start time commenced on melting of the monomer. The reaction was deemed finished once a polymer melt of sufficient viscosity stopped the stirrer bar. The reaction was then quenched in air and the product dissolved in DCM (60 mL) with stirring. The solvent was removed *in vacuo* and a crude ^{1}H NMR ($CDCl_3$) spectrum of the polymer was obtained. The polymer was then washed with copious amounts of MeOH (200 mL) to remove initiator and any unreacted monomer, dried *in vacuo* and retained for materials characterization. *N.B. rac*-LA was recrystallized from anhydrous toluene once prior to use. Monomer conversion was determined *via* ^{1}H NMR analysis of the methine region (*ca.* δ = 4.9 – 5.2 ppm).

Table S1. Melt polymerization of *rac*-LA using Zn(II)-complexes.

Initiator	Time / min	Conversion[a] / %	$M_{n,theo}$[b] / g mol^{-1}	M_n[c] / g mol^{-1}	*Đ*[c]	P_r[d]
Zn(**1**)Et	2	84	36750	65150	3.23	0.57
	2[e]	88	12800	21450	3.83	0.57
	60[f]	55	8050	9200	1.55	0.55
Zn(**2**)Et	4	94	41100	75500[g]	3.97[g]	0.54
Zn(**2**)$_2$	2	85	37200	55800	1.91	0.57
	2[e]	91	13200	19350	1.69	0.58
	60[f]	62	9050	11350	1.69	0.59
	60[h]	24	10600	-[i]	-[i]	-[i]
Zn(**3**)$_2$	20	44	19300	15550[g]	2.38[g]	0.56
[Zn(**3**)Et]$_2$	20	21	9250	-[i]	-[i]	-[i]
Zn(**4**)Et	30	51	21900	25700	4.30	0.56
Zn(**5**)Et	2	78	34150	51450	3.68	0.55

Reaction conditions: *rac*-LA (3.0 g), solvent free (130 °C), {[*rac*-LA]:[M]:[BnOH] = 10000:1:33}. [a] Determined *via* ^{1}H NMR spectroscopy. [b] Theoretical average number molecular weight (M_n) dependent on conversion and co-initiator added {($M_{r,LA}$ × 3.03 × $\%_{conv.}$) + $M_{r,BnOH}$)}. [c] Determined *via* SEC analysis (in THF). [d] Determined *via* homonuclear decoupled NMR spectroscopy. [e] {[*rac*-LA]:[M]:[BnOH] = 10000:1:100}. [f] {[*rac*-LA]:[M]:[BnOH] = 10000:1:100} at 180 °C. [g] Bimodal SEC. Calculated M_n and *Đ* values correspond to peaks treated together. [h] *L*-lactide used (*L*-LA). [i] Insufficient polymeric material isolated for material characterization due to low monomer conversion. *N.B.* [M]:[BnOH] = 1:1 corresponds to 1 equivalent of BnOH per Zn(II) centre.

3.1.1. Representative ^{1}H NMR Spectrum

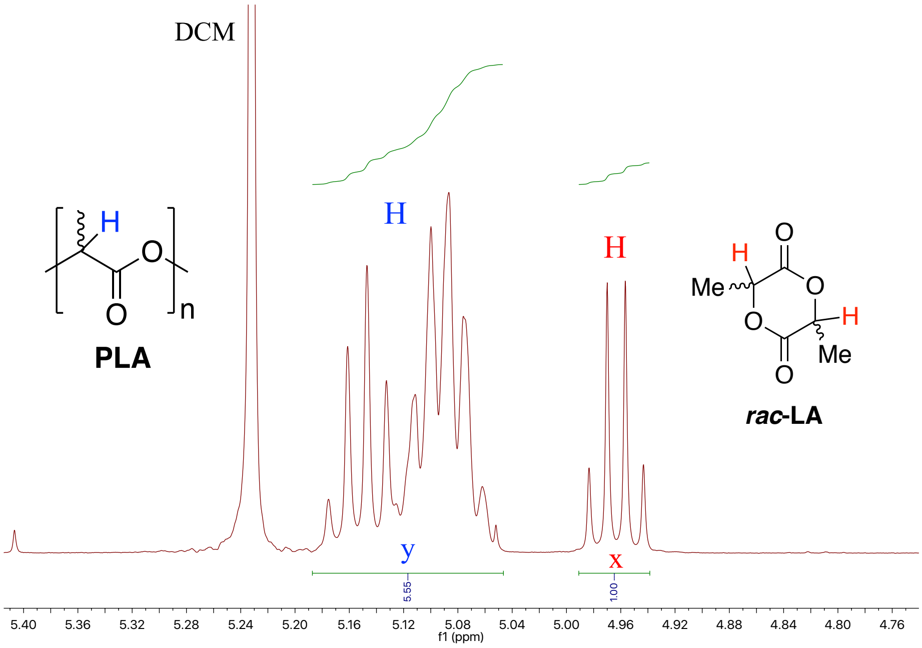

Figure S50. ^{1}H NMR ($CDCl_3$, 500 MHz) spectrum of crude PLA product obtained from the melt polymerization of *rac*-LA at 130 °C using Zn(**2**)$_2$ {[*rac*-LA]:[M]:[BnOH] = 10000:1:33}. PLA conversion = [y/(x+y)] x 100%.

3.1.2. Representative SEC Spectra

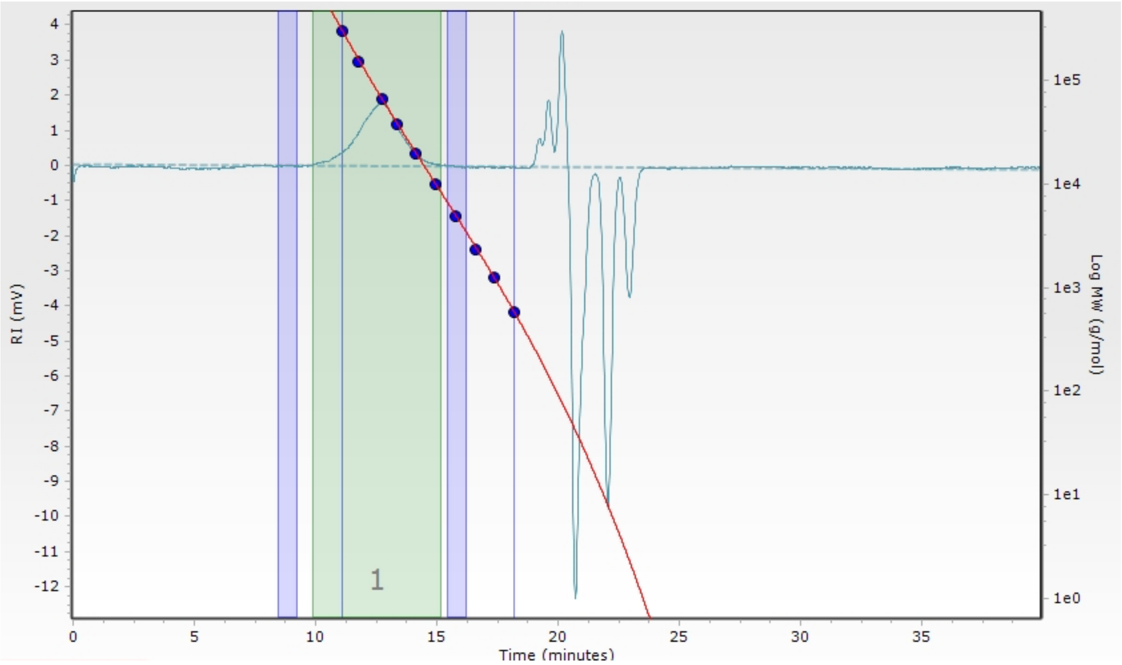

Figure S51. Monomodal SEC spectrum of purified PLA product obtained from the melt polymerization of *rac*-LA at 130 °C using Zn(**2**)$_2$ {[*rac*-LA]:[M]:[BnOH] = 10000:1:33}.

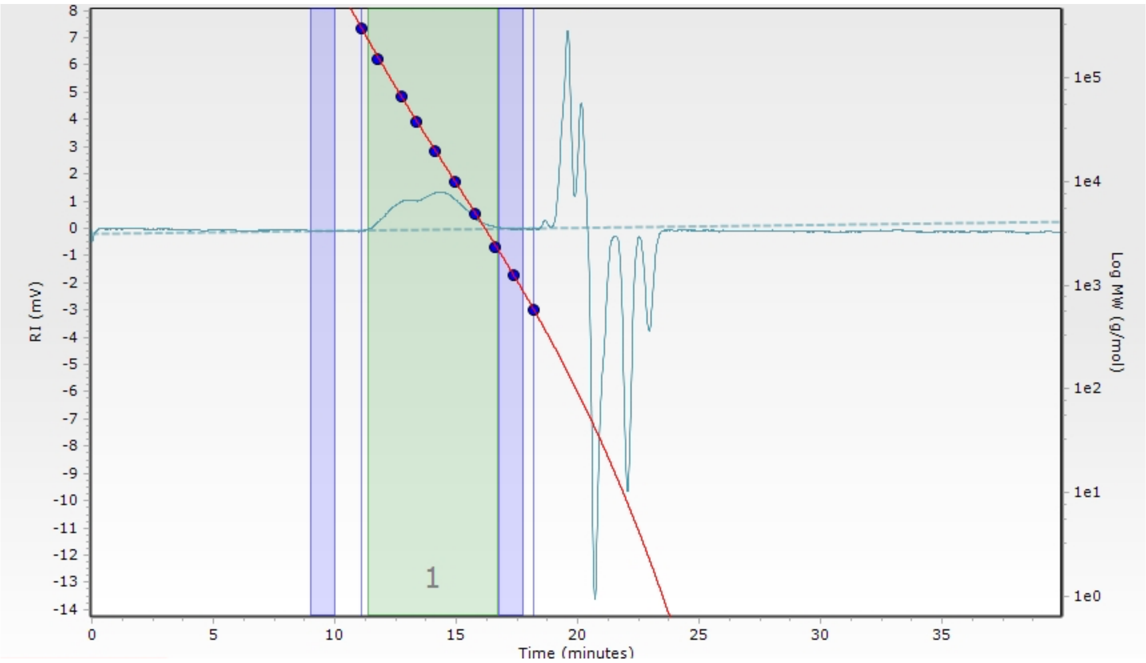

Figure S52. Bimodal SEC spectrum of purified PLA product obtained from the melt polymerization of *rac*-LA at 130 °C using Zn(**3**)$_2$ {[*rac*-LA]:[M]:[BnOH] = 10000:1:33}.

3.1.3. Representative Homonuclear Decoupled ^{1}H NMR Spectrum

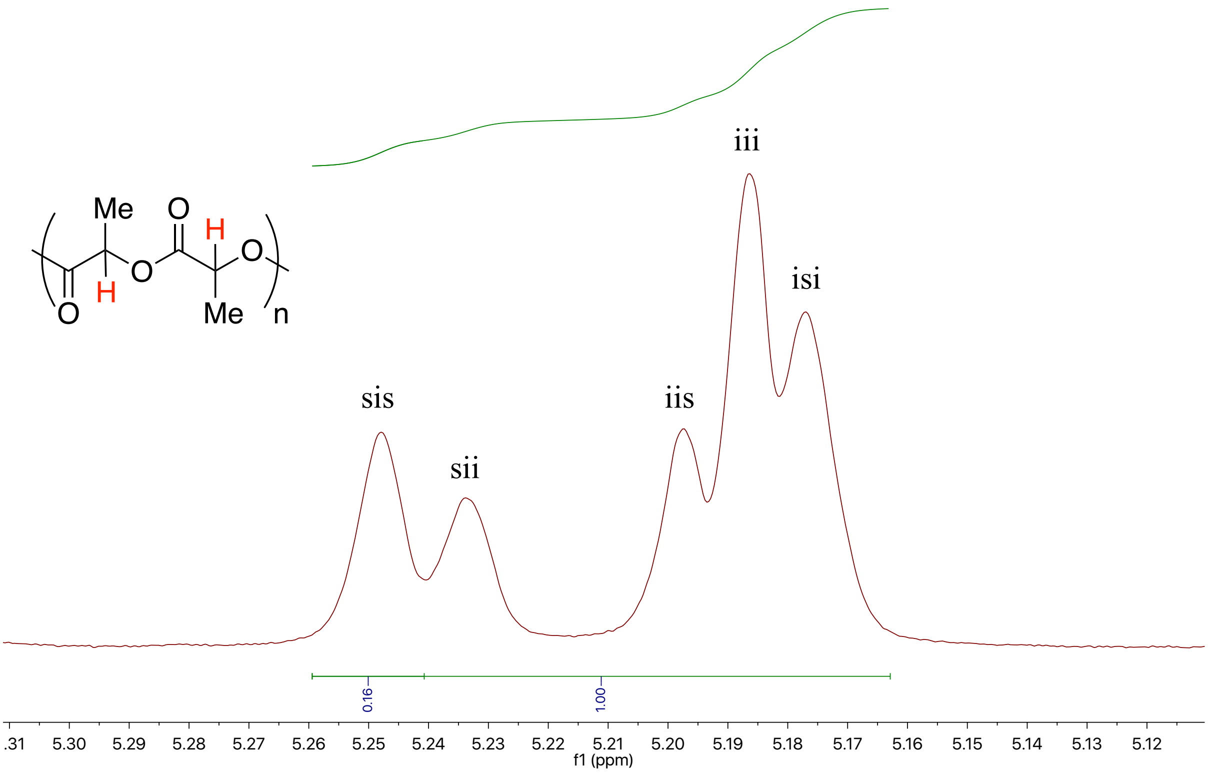

Figure S53. Homonuclear decoupled ^{1}H NMR ($CDCl_3$, 400 MHz) spectrum of crude atactic PLA product (P_r = 0.57) from the melt polymerization of *rac*-LA at 130 °C using Zn(**1**)Et {[*rac*-LA]:[M]:[BnOH] = 10000:1:33}, displaying the five tetrad possibilities in the methine region (red).[3]

3.1.4. MALDI-ToF Spectra

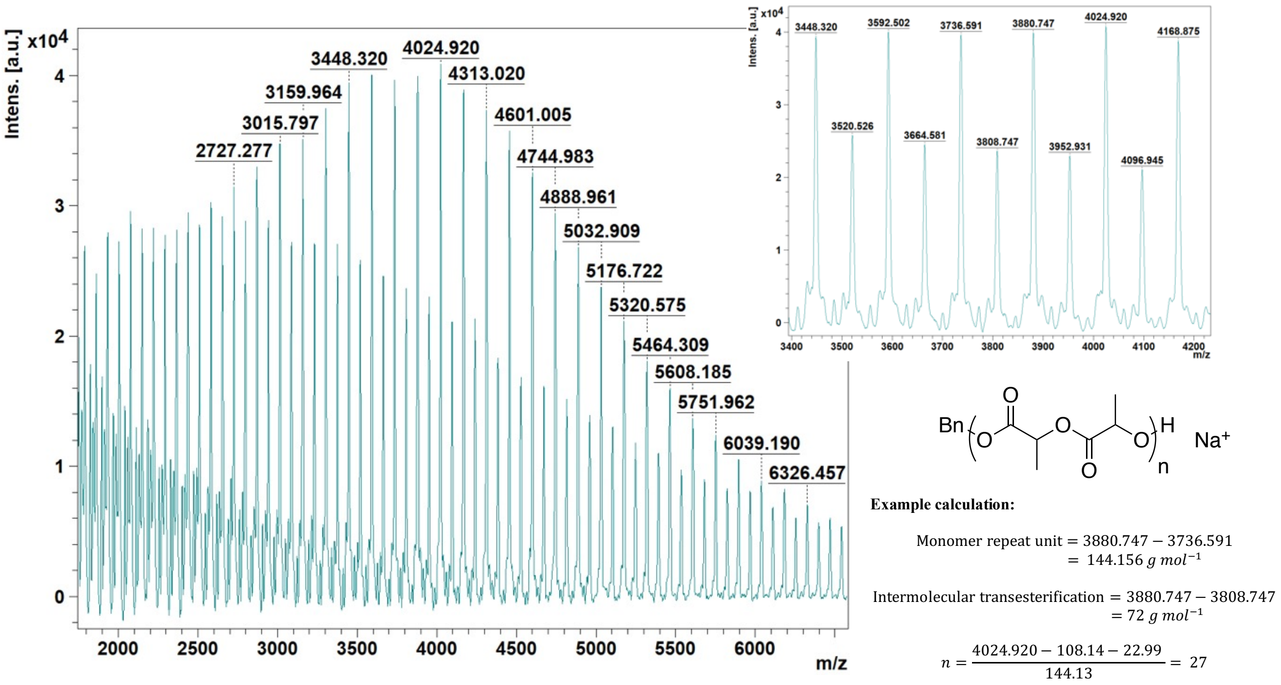

Figure S54. MALDI-ToF spectrum of purified PLA product obtained from the melt polymerization of *rac*-LA using Zn(**1**)Et at 180 °C {[*rac*-LA]:[M]:[BnOH] = 10000:1:100}. Magnified version of secondary series provided to assist in identifying the repeat unit and end group with example calculation provided.

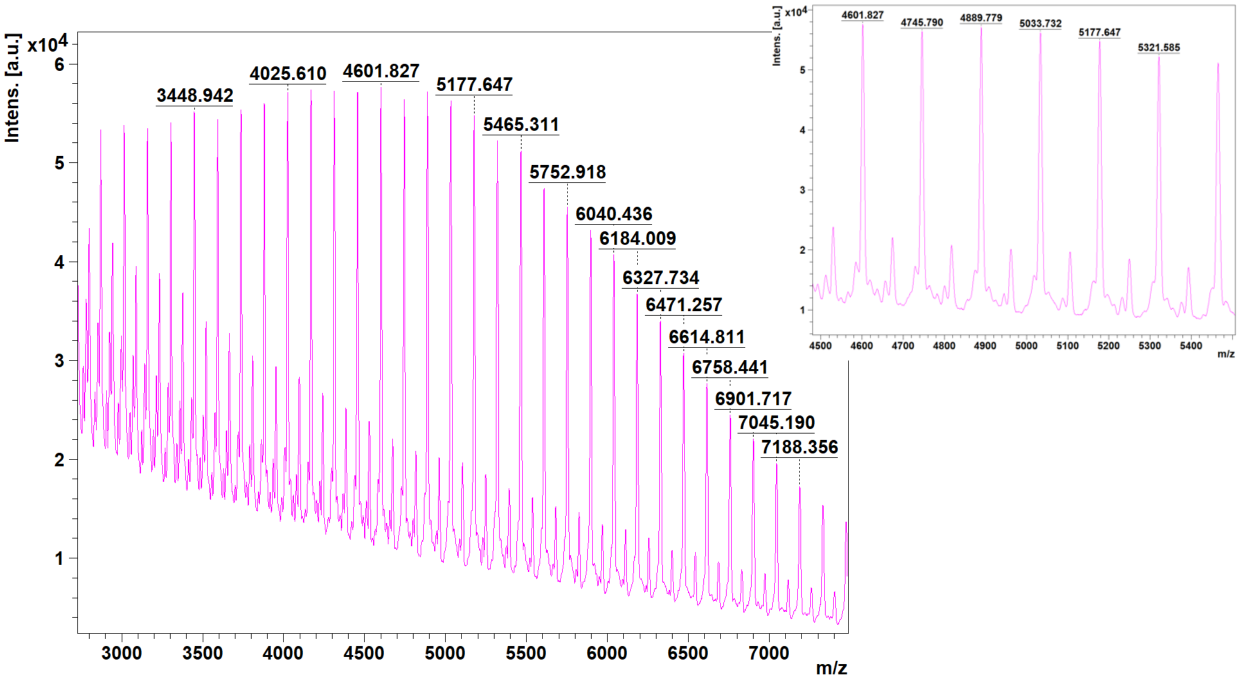

Figure S55. MALDI-ToF spectrum of purified PLA product obtained from the melt polymerization of *rac*-LA using Zn(**2**)$_2$ at 180 °C {[*rac*-LA]:[M]:[BnOH] = 10000:1:100}. Magnified version of secondary series provided to assist in identifying the repeat unit and end group.

4. PLA Degradation

4.1. PLA Methanolysis

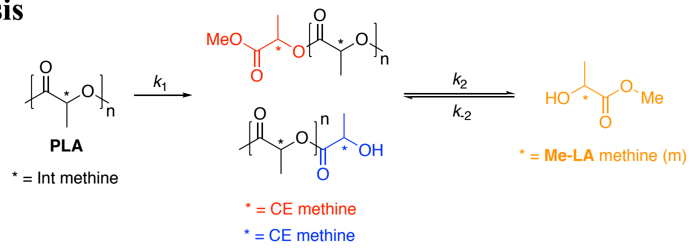

Scheme S3. Two-step reaction sequence for the production of methyl lactate (Me-LA) from PLA *via* the intermediate formation of chain-end groups. Consequently, the methine groups can be categorized as internal (int), chain-end (CE) and those corresponding directly to the alkyl lactate (Me-LA).[4]

General procedure: A J Young's ampoule was charged with metal complex (4 - 8 wt%, 0.01 - 0.02 g, 0.36 – 1.4 mol% relative to ester linkages) and PLA pieces (0.25 g, 0.1 × 0.1 cm^2, Vegware^{TM}, PLLA cup, M_n = 45,510 g mol^{-1}) in a glovebox filled with argon. The polymer was then dissolved in THF (4 mL) with heating and stirring assisting dissolution following submersion in a preheated oil bath (50 °C). MeOH (1 mL) was then added under a flow of argon and aliquots were taken for ^{1}H NMR ($CDCl_3$) analysis of the methine region. After the reaction, the solvent was removed *in vacuo* and the residual methyl lactate (Me-La) was analyzed further.

Table S2. PLA methanolysis using Zn(II)-complexes.

Catalyst	Time / h	T / °C	Cataylst loading / wt%	$Y_{Me\text{-}LA}$ / %	$S_{Me\text{-}LA}$ / %	X_{int} / %
Zn(**1**)Et	3	50	4	7	16	44
Zn(**2**)Et	3	50	4	2	10	22
Zn(**2**)$_2$	3	50	4	70	72	97
				0[a]	0[a]	22[a]
				0[b]	0[b]	17[b]
				22[c]	32[c]	67[c]
				12[d]	24[d]	70[d]
	1.5	50	8	94	94	100
Zn(**3**)$_2$	3	50	4	9	19	49
	3	50	8	42	48	89
[Zn(**3**)Et]$_2$	3	50	4	5	11	45
Zn(**4**)Et	3	50	4	5	13	35
	3	50	8	29	36	81
Zn(**5**)Et	3	50	4	62	64	96
	1.5	50	8	85	85	100

Reaction conditions: PLLA cup (0.25 g, M_n = 45510 g mol^{-1}), V_{THF}:V_{MeOH} = 4:1, n_{MeOH}:n_{ester} = 7:1, Zn(II)-complex = 4 – 8 wt% (0.01 – 0.02 g, 0.36 – 1.4 mol% relative to ester linkages). $Y_{Me\text{-}LA}$, $S_{Me\text{-}LA}$ *and* X_{int} determined by ^{1}H NMR ($CDCl_3$) upon solvent removal. [a] Solvent: 2-methyltetrahydrofuran (2-Me-THF). [b] PLA-based 3D printing material. [c] Mixed feed: PLLA cup (0.25 g, M_n = 45510 g mol^{-1}) + bottle-grade PET (0.25 g, M_n ~ 40000 g mol^{-1}). [d] Mixed feed: PLLA cup (0.25 g, M_n = 45510 g mol^{-1}) + bottle-grade PET (0.25 g, M_n ~ 40000 g mol^{-1}) + PVC (0.025 g, 10 wt%, M_n = 22000 g mol^{-1}).

4.1.1. Degradation Kinetics

Reaction kinetic analysis was performed as described for the degradation procedure in section 4.1. Aliquots were taken under a dynamic flow of argon to obtain a minimum of 4 data points across the total reaction time. The pseudo-first-order rate constant (k_{app}) was determined by plotting $\ln([Int]_0/[Int]_t)$ against time.

Table S3. PLLA cup methanolysis using Zn(**2-3**)$_2$ and Zn(**4-5**)Et in THF.

Catalyst	Time / h	T / °C	Catalyst loading / wt%	Y_{Me-LA} / %	k_{app} / min^{-1}
Zn(**2**)$_2$	3	50	4	71	0.026 ± 0.00077
	1.5	50	8	76	0.053 ± 0.0016
Zn(**3**)$_2$	3	50	8	42	0.015 ± 0.0010
Zn(**4**)Et	3	50	8	27	0.014 ± 0.00027
Zn(**5**)Et	1.5	50	8	63	0.044 ± 0.0018

Reaction conditions: PLLA cup (0.25 g, M_n = 45510 g mol^{-1}), V_{THF}:V_{MeOH} = 4:1, n_{MeOH}:n_{ester} = 7:1, Zn(II)-complex = 4 – 8 wt% (0.01 – 0.02 g, 0.36 – 1.4 mol% relative to ester linkage). *N.B.* Y_{Me-LA} refers to maximum Me-LA conversion in solution determined *via* ^{1}H NMR ($CDCl_3$).

4.1.2. Representative ^{1}H NMR Spectra

Int conversion (X_{Int}), Me-La selectivity (S_{Me-La}) and Me-La yield (Y_{Me-La}) were calculated using Equations 1 to 3 below:[4]

$$X_{int} = 1 - \frac{[Int]}{[Int]_0} \quad (1)$$

$$S_{Me\text{-}LA} = \frac{[Me-LA]}{[Int]_0-[Int]} \quad (2)$$

$$Y_{Me\text{-}LA} = X_{int}\, S_{Me\text{-}LA} \quad (3)$$

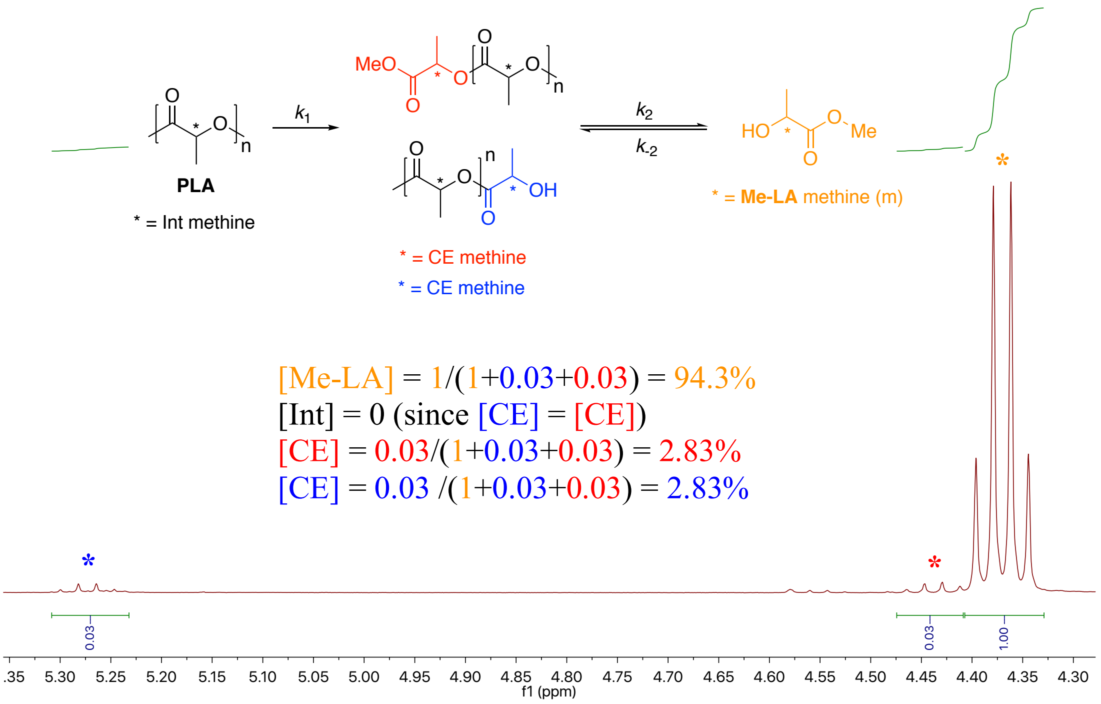

Figure S56. ^{1}H NMR ($CDCl_3$, 400 MHz) spectrum of PLA Vegware cup degradation into methyl lactate (Me-LA) using Zn(**2**)$_2$ (8 wt % cat. loading, 0.72 mol% relative to ester linkages) at 50 °C for 1.5 h in THF (solvent removed). *N.B.* [Int] = 0 indicative of complete consumption of the PLA cup. Consequently, no internal methine (*) resonance was observed, which would appear in the same region as (*).

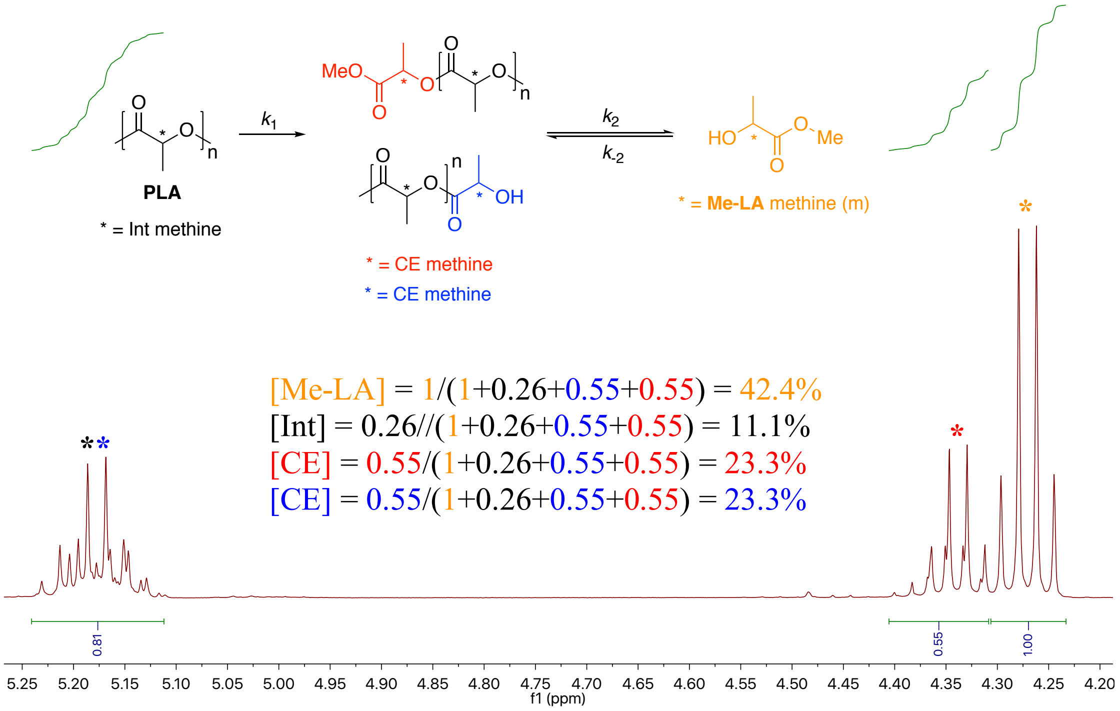

Figure S57. ^{1}H NMR ($CDCl_3$, 400 MHz) spectrum of PLA Vegware cup degradation into methyl lactate (Me-LA) using Zn(**3**)$_2$ (8 wt % cat. loading, 1.2 mol% relative to ester linkages) at 50 °C for 3 h in THF (solvent removed).

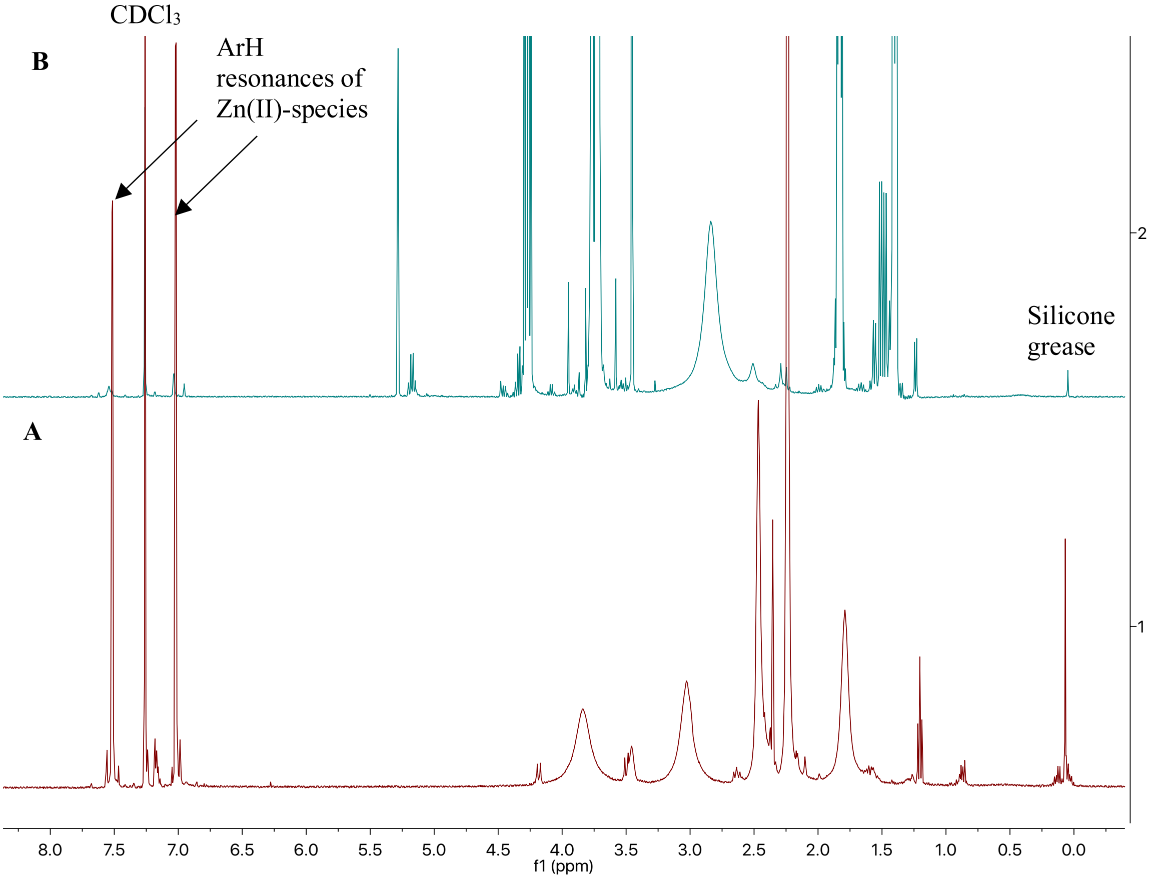

Figure S58. Stacked ^{1}H NMR ($CDCl_3$, 500 (**A**) and 400 (**B**) MHz) spectrum assessing the stability of Zn(**2**)$_2$ before and after PLA methanolysis: (**A**) Zn(**2**)$_2$, and; (**B**) PLA Vegware cup degradation into methyl lactate (Me-LA) using Zn(**2**)$_2$ (8 wt % cat. loading, 0.72 mol% relative to ester linkages) at 50 °C for 1.5 h in THF (solvent removed).

5. PET Degradation

5.1. PET Glycolysis

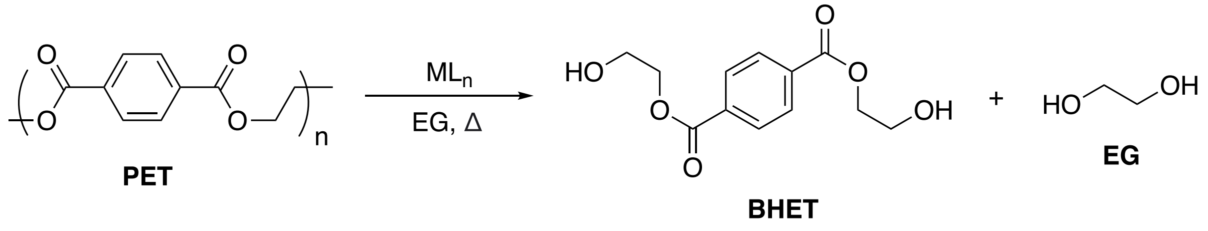

Scheme S4. Metal-mediated glycolysis of PET into BHET and EG.

General procedure A J Young's ampoule was charged with Zn(**2**)$_2$ (4 – 8 wt%, 0.01 – 0.02 g, 0.96 – 1.90 mol% relative to ester linkages) and bottle-grade PET pieces (0.25 g, 0.1 × 0.1 cm^2, *The Coca-Cola Company*^{TM}, M_n ~ 40,000 g mol^{-1}) in a glovebox filled with argon. Ethylene glycol (EG) (1.5 mL, 20.6 equivalents relative to ester linkages) was added under a flow of argon before submerging the ampoule in a preheated oil bath (180 °C) and stirred until a homogenous solution was observed, indicating complete consumption of PET. Following this the ampoule was removed and allowed to cool for a few minutes after which deionized H_2O (5 mL) was added, the ampoule vigorously shaken and rapidly filtered. The ampoule was rinsed with an additional portion of deionized H_2O (5 mL), which was used to stir the filter cake. The filtrate was retained and refrigerated (*ca.* 4 °C) for 18 h. Needle-like crystals of bis(2-hydroxyethyl) terephthalate (BHET) were isolated by filtration, washed with deionized H_2O (2 × 1 mL) and dried *in vacuo* at 90 °C for 3 h.

Table S4. PET glycolysis using Zn(**2**)$_2$ at 180 °C.

Catalyst	Time / h	T / °C	Catalyst loading / wt%	EG / equiv.	Y_{BHET} / g
Zn(OAc)$_2$·2H_2O	3[a]	180	4	20.6	0.17 (52%)
	4	180	8	20.6	0.14 (42%)
Zn(**2**)$_2$	3	180	4	20.6	0.14 (42%)
	1[a]	180	4	20.6	0.20 (61%)
	3[b]	180	4	20.6	0.06 (18%)
	1.5	180	8	20.6	0.16 (48%)

Reaction conditions: Bottle-grade PET (0.25 g, M_n ~ 40000 g mol^{-1}), 20.6 (1.5 mL) equiv. of EG (relative to ester linkages), Zn(OAc)$_2$·2H_2O = 4 – 8 wt% (0.01 – 0.02 g, 3.4 – 6.5 mol% relative to ester linkages), Zn(**2**)$_2$ = 4 – 8 wt% (0.01 – 0.02 g, 0.96 – 1.9 mol% relative to ester linkages). Y_{BHET} refers to the isolated yield of pure BHET recrystallized from deionized H_2O followed by drying at 90 °C for 3 h *in vacuo*. Both mass (g) and % yield (bracketed value) are reported for Y_{BHET}. [a] PET thin-film (0.25 g). [b] Mixed feed: bottle-grade PET (0.25 g, M_n ~ 40000 g mol^{-1}) + PVC (0.025 g, 10 wt%, M_n = 22000 g mol^{-1}).

5.1.1. Representative ^{1}H NMR Spectrum

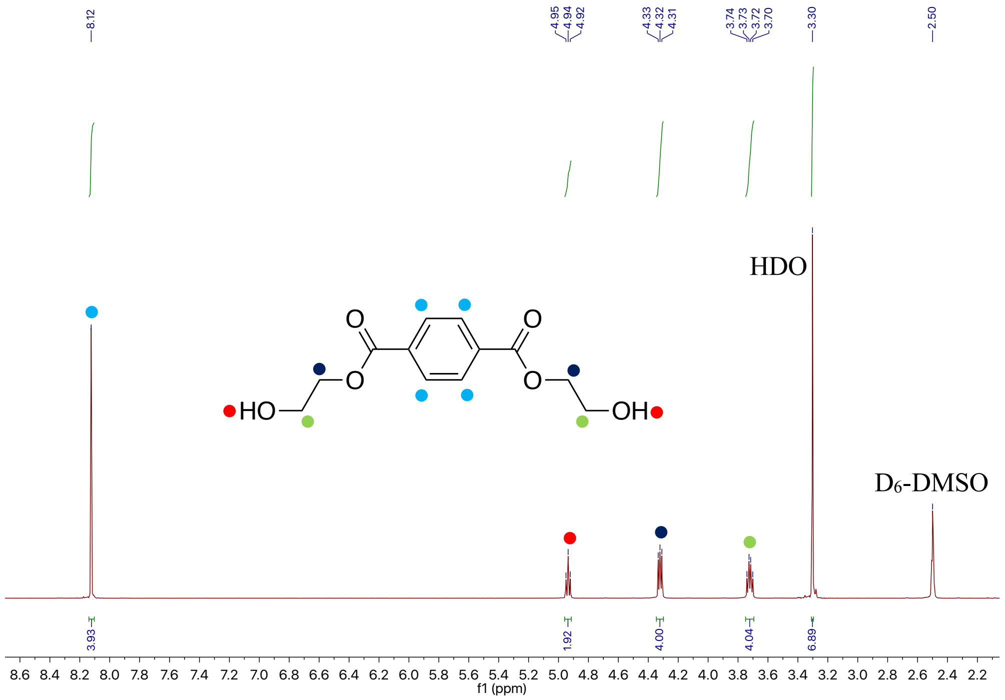

Figure S59. ^{1}H NMR (D_6-DMSO, 400 MHz) spectrum of recrystallized BHET obtained from the glycolysis of bottle-grade PET using EG (1.5 mL, 20.6 equivalents) at 180 °C for 1.5 h using Zn(**2**)$_2$ (8 wt% cat. loading, 1.90 mol% relative to ester linkages).

5.2. PET Aminolysis

Diamine terephthalamide products derived from PET aminolysis were prepared in accordance to methods described previously by Fukushima *et al.*[5]

Table S5. PET aminolysis into terephthalamides using Zn(**2**)$_2$.

Catalyst	Time / h	T / °C	Cat. loading / wt%	Amine / equiv.	TPA	Y_{BHET} / g
No cat.	2	110	-	16	BAETA	0.034 (11%)
Zn(**2**)$_2$	1	110	8	16		0.11 (36%)
No cat.	3	120	-	6.4	BHETA	0.28 (88%)
Zn(**2**)$_2$	2	120	8	6.4		0.29 (93%)

Reaction conditions: Bottle-grade PET (0.25 g, M_n ~ 40000 g mol^{-1}), Zn(**2**)$_2$ = 8 wt% (0.02 g, 1.9 mol% relative to ester linkages). Ethylenediamine (16 equiv.) and ethanolamine (6.4 equiv.) were used for the production of BAETA and BHETA respectively. Y_{TPA} refers to the isolated yield of terephthalamide. Both mass (g) and % yield (bracketed value) are reported for Y_{TPA}.

5.2.1. *N,N'*-bis(2-aminoethyl)terephthalamide (BAETA)

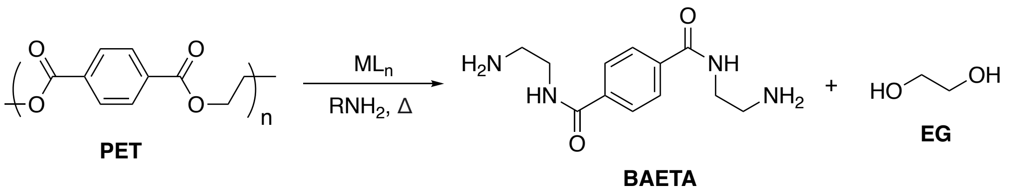

Scheme S5. Metal-mediated aminolysis of PET into BAETA and EG.

A J Young's ampoule was charged with bottle-grade PET pieces (0.24 g, 0.1 × 0.1 cm^2, *The Coca-Cola Company^{TM}, M_n* ~ 40,000 g mol^{-1}) and Zn(**2**)$_2$ (8 wt% cat. loading, 0.019 g, 1.90 mol% relative to ester linkages) in a glovebox filled with argon. Ethylenediamine (1.34 mL, 20 mmol) was added under a dynamic flow of argon. The ampoule was submerged in a preheated oil bath (110 °C) and stirred for 1 h, observing the formation of an off-white slurry. The homogenous slurry was poured into hot toluene (25 mL) and filtered hot. The residual solid was redissolved in hot MeOH (25 mL) and filtered hot to remove a small amount of insoluble. The filtrate was collected and the solvent removed *in vacuo* to afford an off-white solid, which was washed with copious amounts of isopropanol (50 mL) and dried *in vacuo* at 100 °C. The product was isolated as a white powder.

^{1}H NMR (D_6-DMSO, 500 MHz): δ = 8.52 (t, J = 5 Hz, 2H; NH), 7.91 (s, 4H; ArH), 3.33 (br s, 4H; NH_2), 3.26 (q, J = 6 Hz, 4H; CH_2), 2.68 (t, J = 6.5 Hz, 4H; CH_2). *N.B.* HSQC analysis assisted with -CH, -NH and -NH_2 resonance assignment. Resonance at δ = 3.26 ppm observed as a quartet due to coupling with both -NH and -CH_2, contrary to Fukushima *et al.*[5]

^{13}C{^{1}H} NMR (D_6-DMSO, 125 MHz): δ = 165.7 (ArCO), 136.7, 127.1 (Ar), 43.2, 41.3 (CH_2). *N.B.* peak assignments consistent with literature example.[5]

Yield = 0.11 g, 36

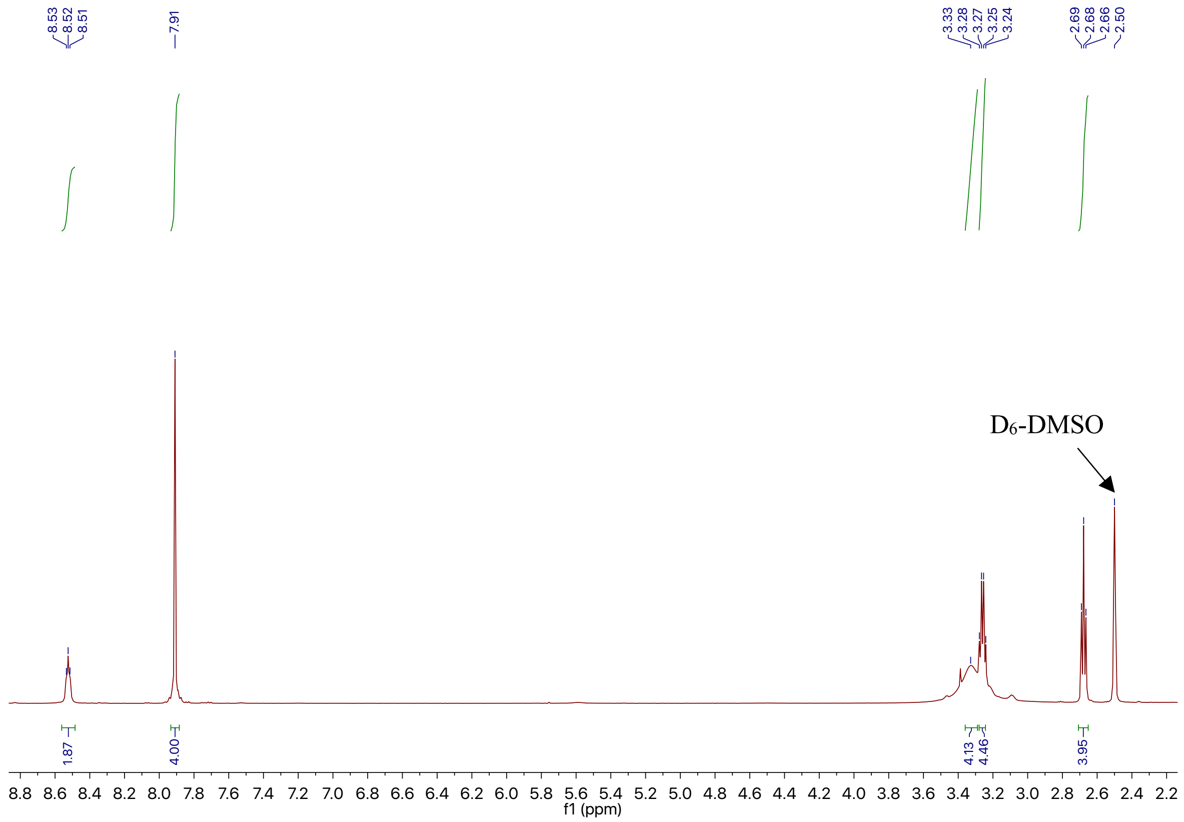

Figure S60. ^{1}H NMR (D_6-DMSO, 500 MHz) spectrum of BAETA.

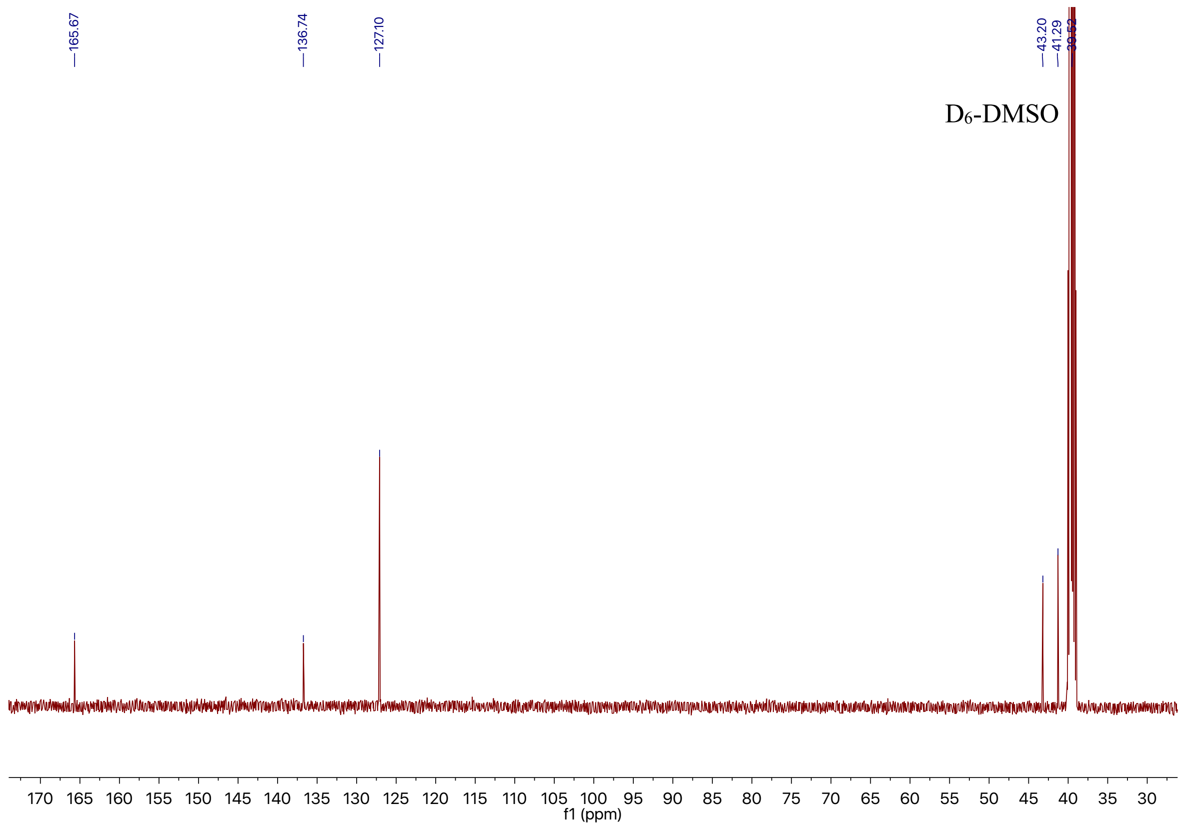

Figure S61. $^{13}C\{^{1}H\}$ NMR (D_6-DMSO, 125 MHz) spectrum of BAETA.

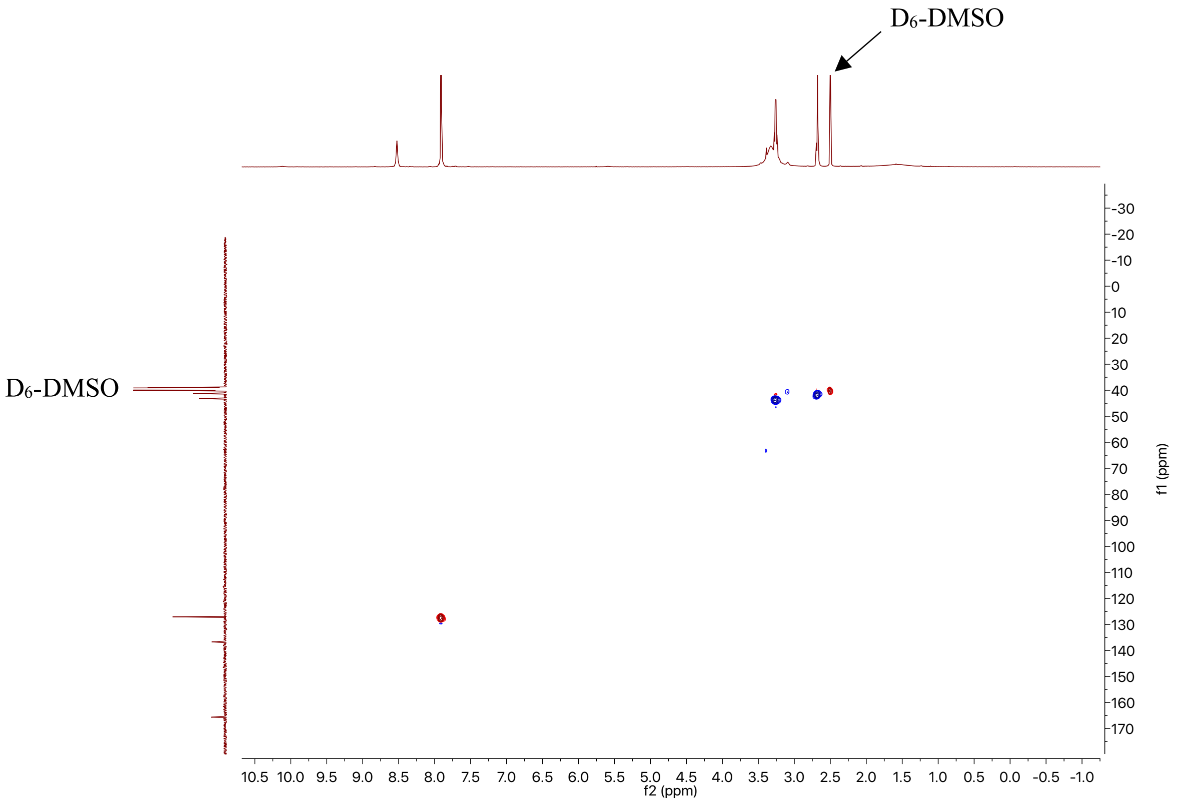

Figure S62. HSQC NMR (D_6-DMSO, 125 MHz) spectrum of BAETA.

5.2.2. *N,N'*-bis(2-hydroxyethyl)terephthalamide (BHETA)

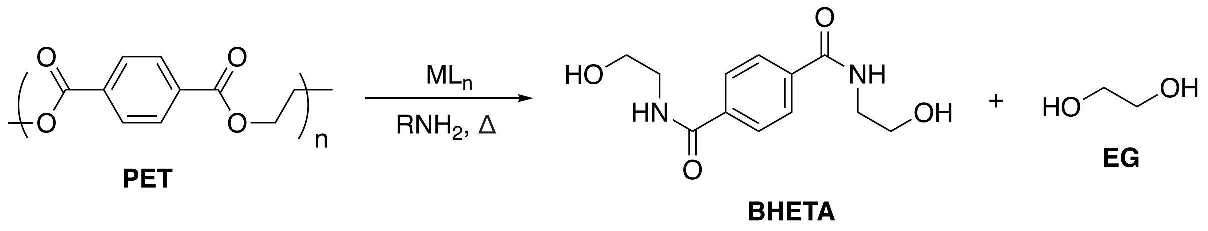

Scheme S6. Metal-mediated aminolysis of PET into BHETA and EG.

A J Young's ampoule was charged with bottle-grade PET pieces (0.24 g, 0.1 × 0.1 cm^2, *The Coca-Cola Company™, M_n* ~ 40,000 g mol^{-1}) and Zn(**2**)$_2$ (8 wt% cat. loading, 0.019 g, 1.90 mol% relative to ester linkages) in a glovebox filled with argon. Ethanolamine (0.5 mL, 8 mmol) was added under a dynamic flow of argon. The ampoule was submerged in a preheated oil bath (120 °C) and stirred for 2 h, observing the formation of a white slurry. The slurry was washed in DCM (25 mL), filtered and washed with THF (25 mL) to afford *N,N'*-bis(2-hydroxyethyl)terephthalamide as a white powder, which was dried *in vacuo.*

^1H NMR (*D_6*-DMSO, 400 MHz): δ = 8.52 (t, *J* = 6 Hz, 2H; NH), 7.91 (s, 4H; ArH), 4.71 (t, *J* = 6 Hz, 2H; OH), 3.52 (q, *J* = 6 Hz, 4H; CH_2OH), 3.34 (q, *J* = 6 Hz, 4H; CH_2NH). *N.B.* All peak assignments consistent with literature example.[5]

$^{13}C\{^1H\}$ NMR (*D_6*-DMSO, 125 MHz): δ = 165.6 (ArCO), 136.7, 127.1 (Ar), 59.7, 42.2 (CH_2). *N.B.* All peaks assignment consistent with literature example.[5]

Yield = 0.29 g, 93%

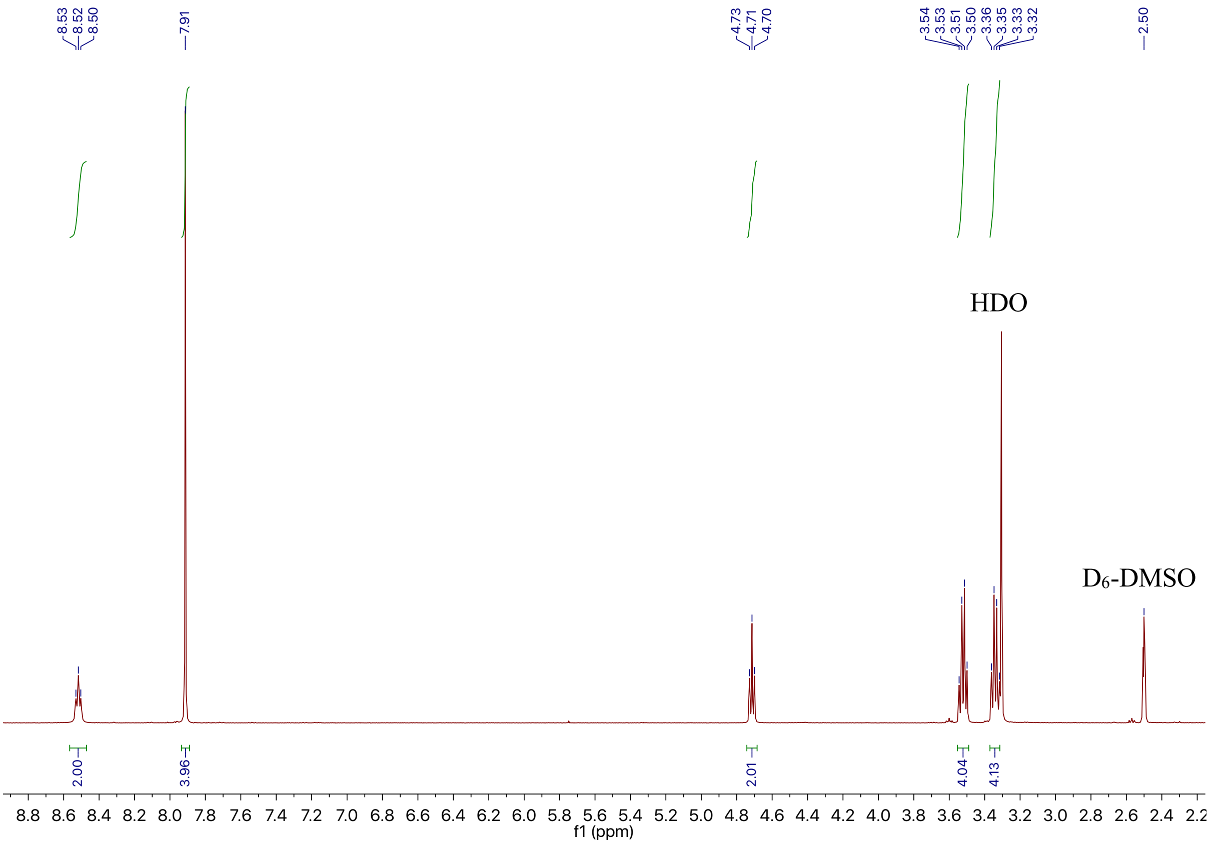

Figure S63. ^{1}H NMR (D_6-DMSO, 400 MHz) spectrum of BHETA.

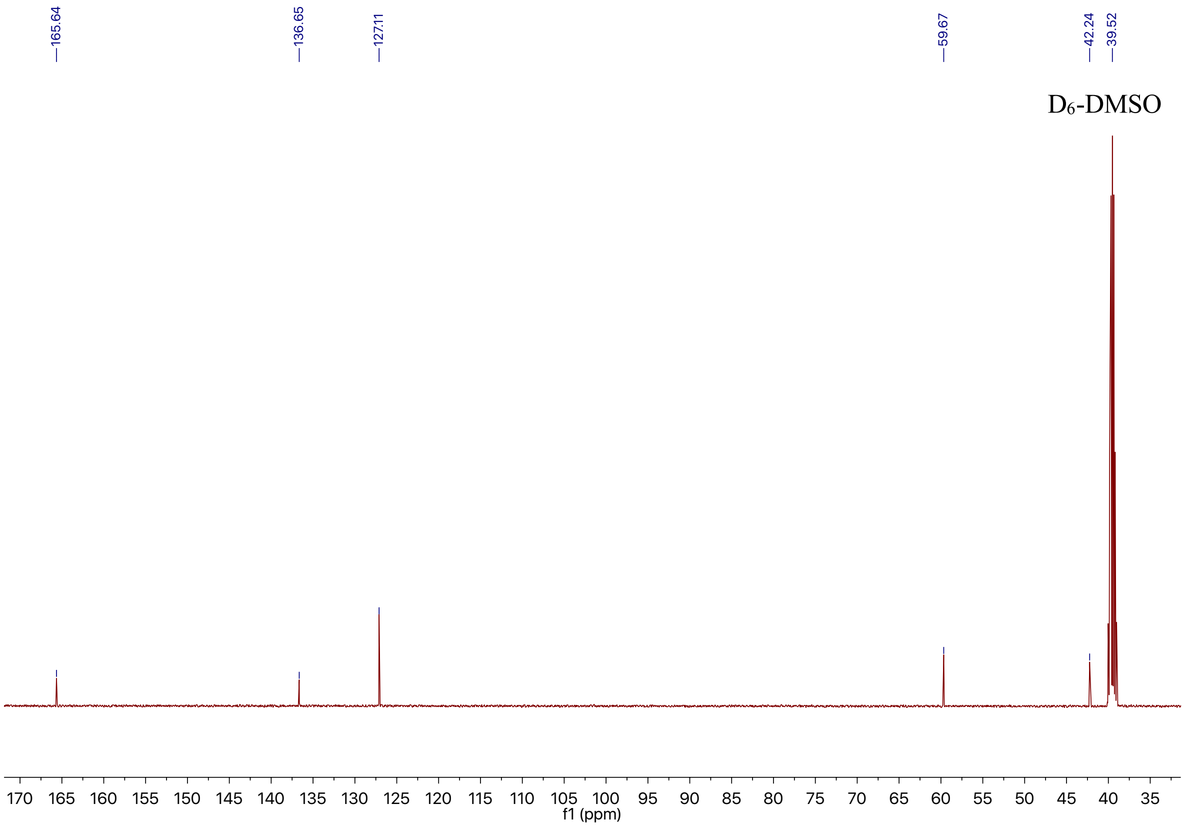

Figure S64. $^{13}C\{^1H\}$ NMR (D_6-DMSO, 125 MHz) spectrum of BHETA.

5.3. Renewable Poly(ester-amide)s (PEAs)

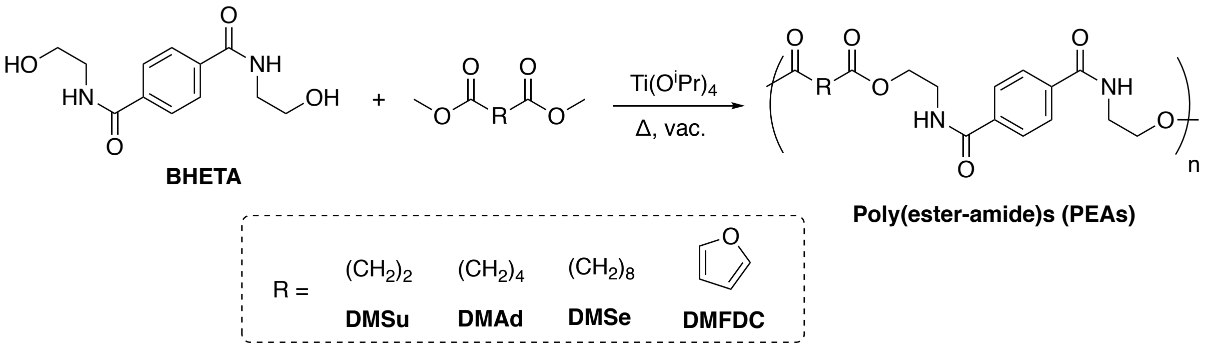

Scheme S7. Melt polycondensation of BHETA with various renewable diesters for the production of poly(ester-amide)s (PEAs).

5.3.1. Melt Polycondensation Method

General procedure: Diester (0.13 – 0.21 g, 0.92 mmol) and BHETA (0.23 g, 0.92 mmol) were charged into a glass vial with a magnetic stirrer bar to which Ti(O^{i}Pr)$_4$ catalyst (400 ppm, stock solution, 20 μL mL^{-1} in toluene) was added. The vial was then positioned within the heating block, which was connected to a vacuum/argon line. The heating block was evacuated once and purged with argon for at least 45 minutes before initiating the reaction. The first transesterification step was conducted under a constant flow of argon for 1 h at both 190 and 200 °C, and then at 210 °C for 30 minutes. The polycondensation step was carried out at 210 °C for 3 hours under dynamic vacuum (< 1 mbar). Finally, the heating was stopped, and the vacuum removed with the introduction of argon. The samples were allowed to cool gradually to room temperature and retained for materials characterization. Samples were analyzed as prepared and without any purification.

5.3.2. Poly(ester-amide) Characterization

5.3.2.1. ^{1}H NMR spectra

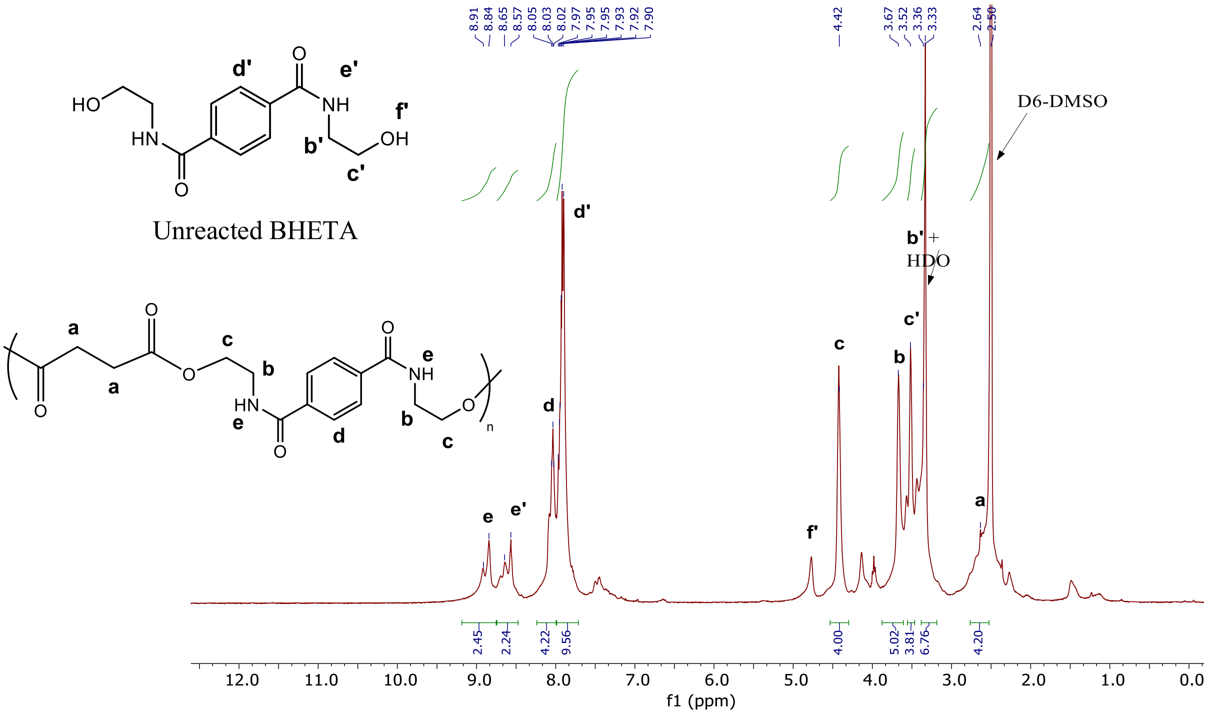

Figure S65. ^{1}H NMR (D_6-DMSO, 500 MHz) spectrum of poly(ester-amide) produced from the melt polycondensation of BHETA and DMSu in the presence of $Ti(O^iPr)_4$.

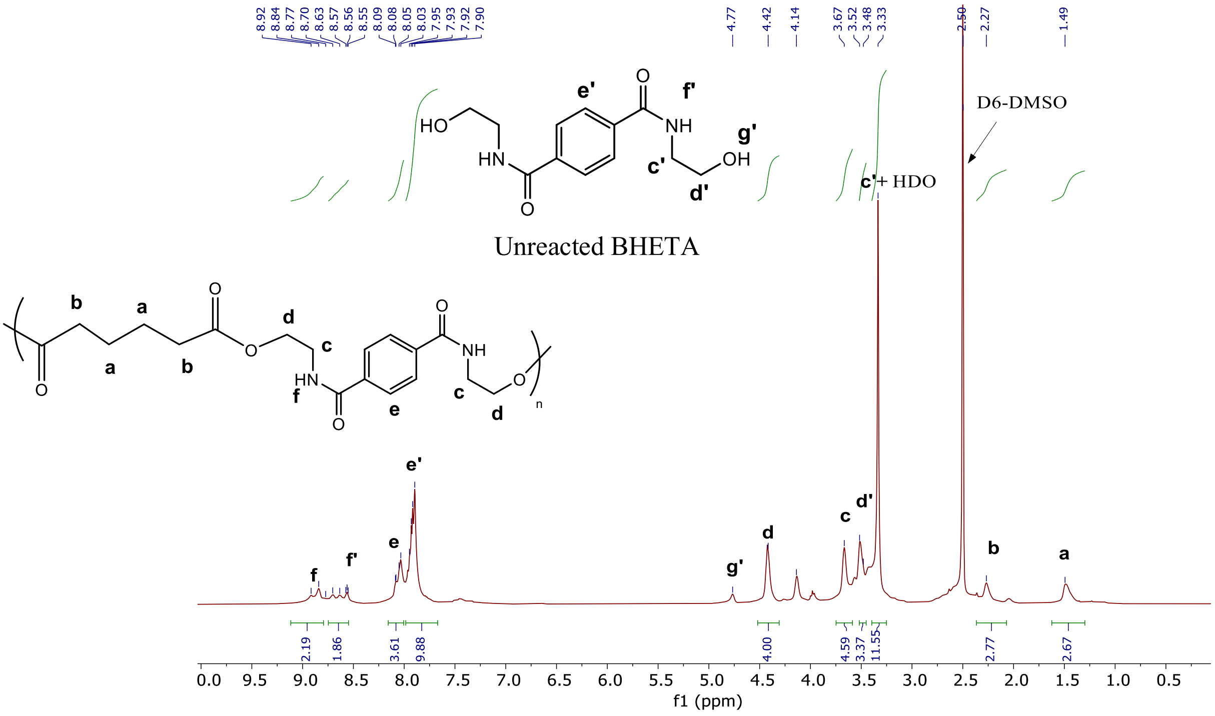

Figure S66. ^{1}H NMR (D_6-DMSO, 500 MHz) spectrum of poly(ester-amide) produced from the melt polycondensation of BHETA and DMAd in the presence of $Ti(O^iPr)_4$.

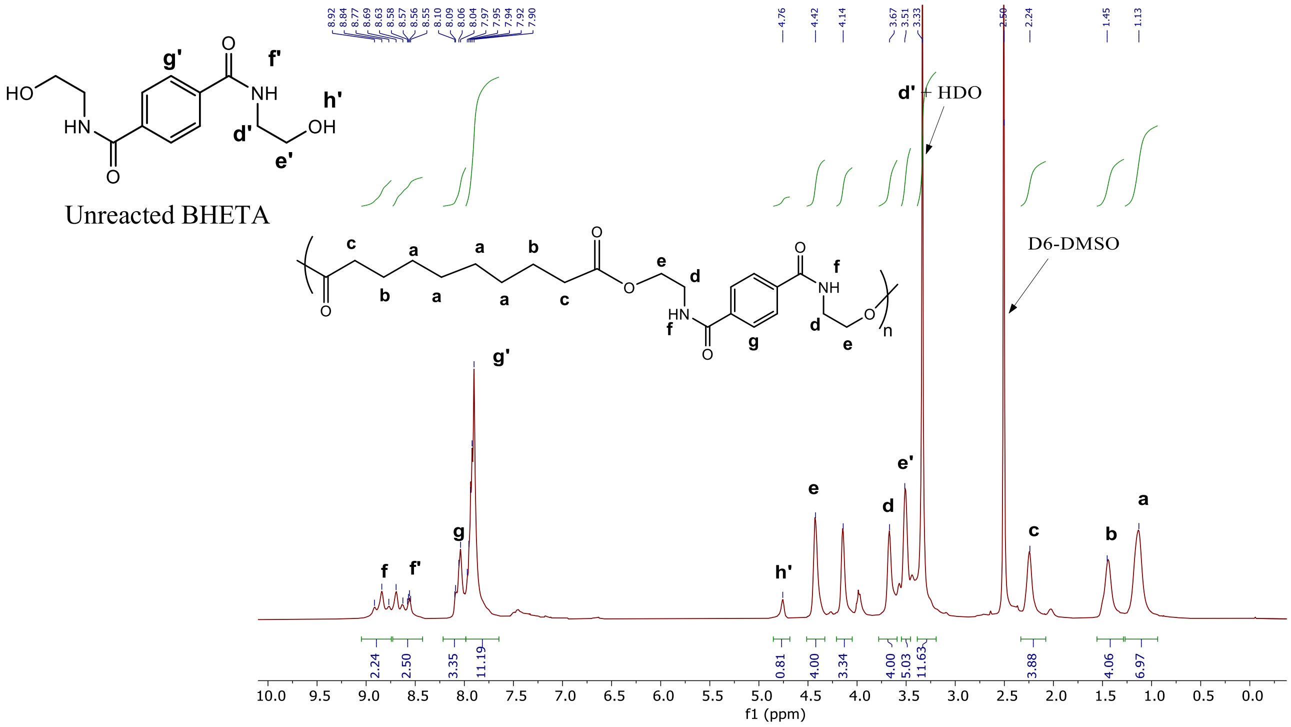

Figure S67. ^{1}H NMR (D_6-DMSO, 500 MHz) spectrum of poly(ester-amide) produced from the melt polycondensation of BHETA and DMSe in the presence of Ti(O^{i}Pr)$_4$.

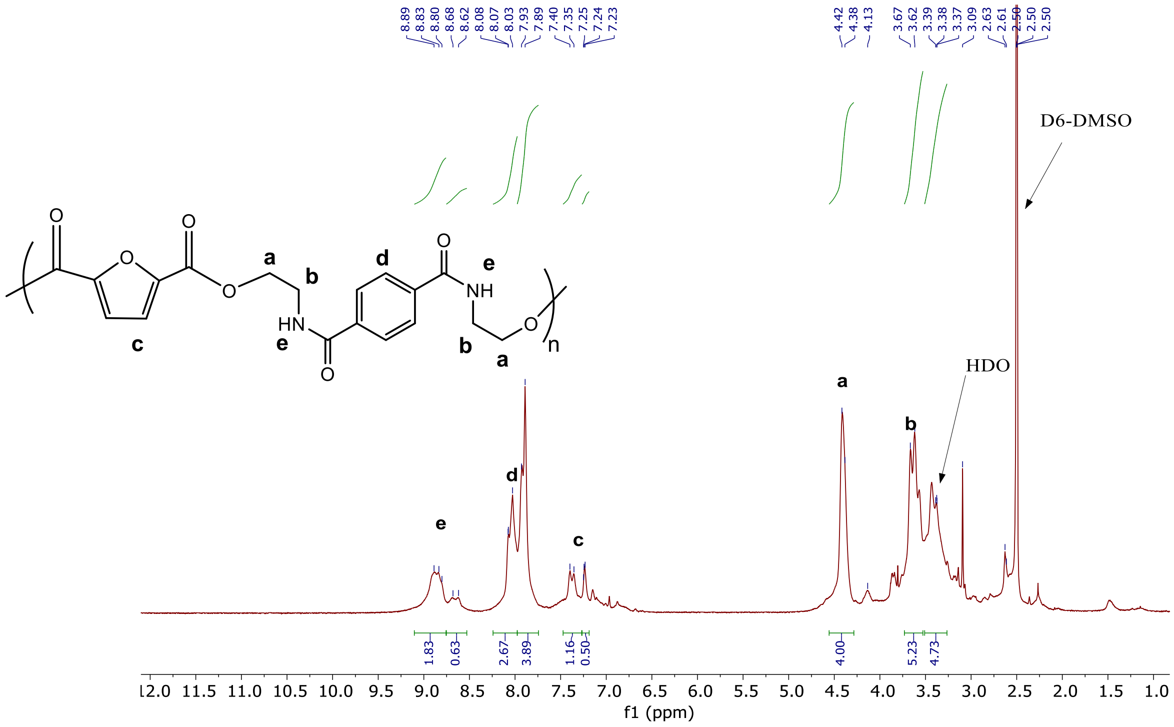

Figure S68. ^{1}H NMR (D_6-DMSO, 500 MHz) spectrum of poly(ester-amide) produced from the melt polycondensation of BHETA and DMFDC in the presence of Ti(O^{i}Pr)$_4$.

5.3.2.2. Representative SEC spectrum

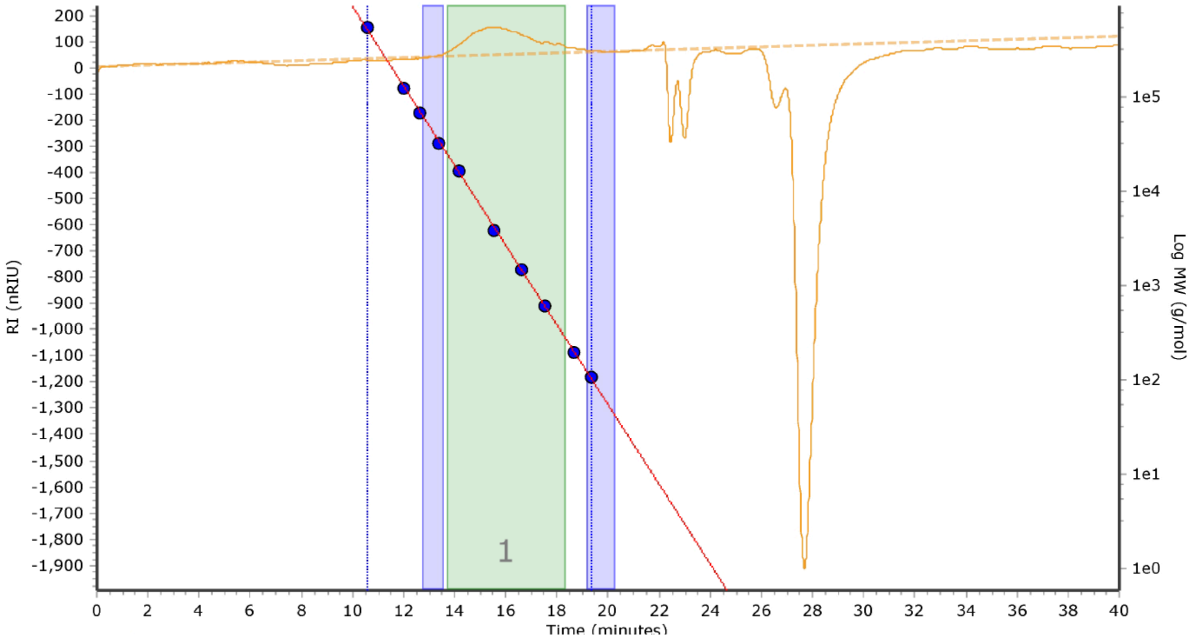

Figure S69. Monomodal SEC spectrum of poly(ester-amide) produced from the melt polycondensation of BHETA and DMFDC in the presence of Ti(O^{i}Pr)$_4$.

5.3.2.3. Thermal Analysis

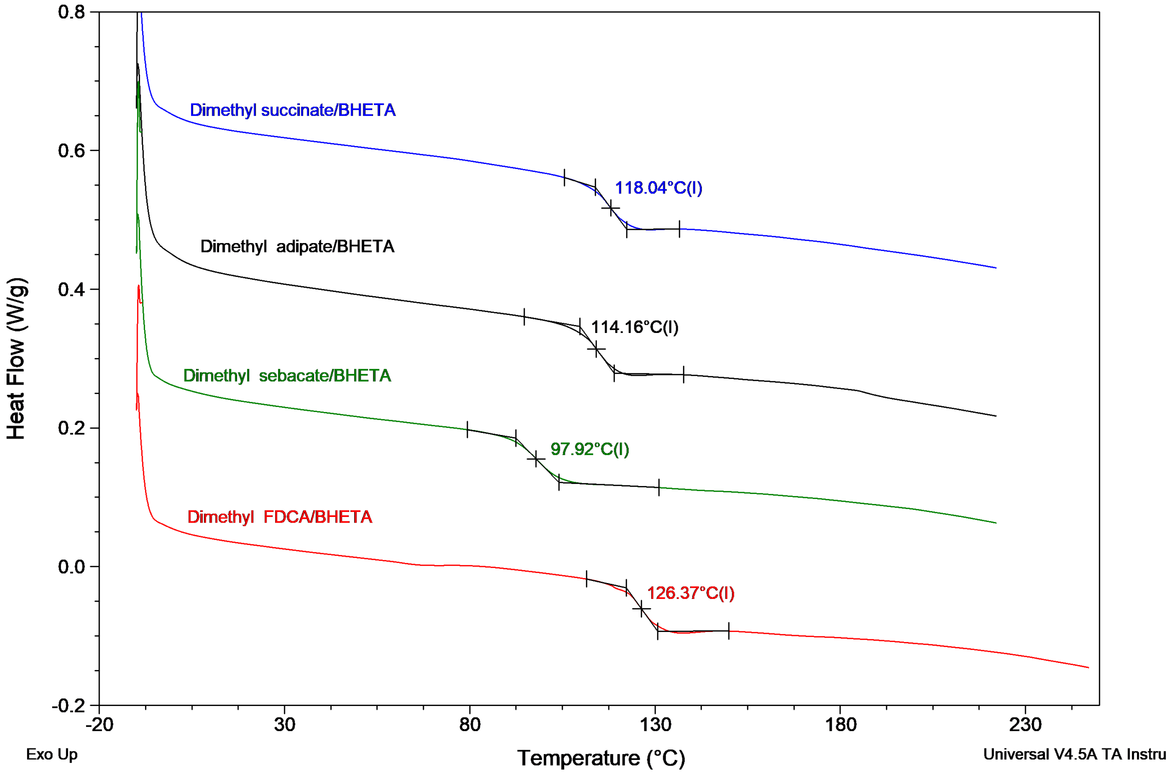

Figure S70. Stacked DSC traces of poly(ester-amide)s produced from the melt polycondensation of BHETA and renewable diesters in the presence of Ti(O^{i}Pr)$_4$.

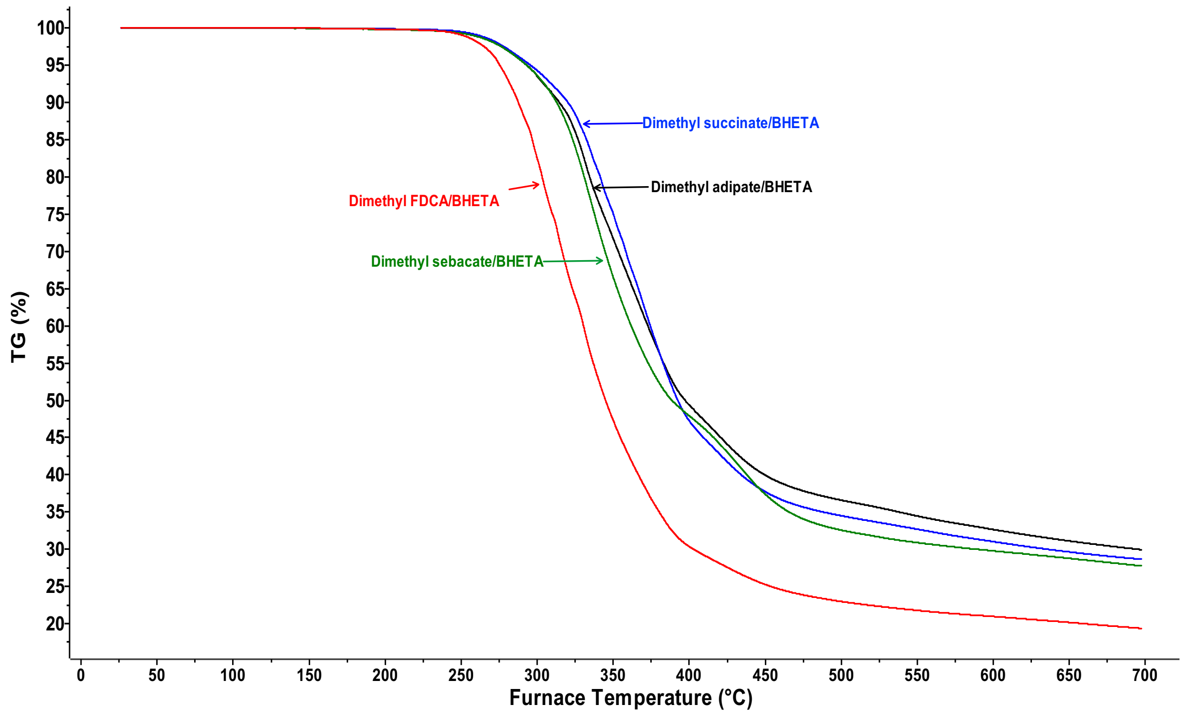

Figure S71. TGA plot of poly(ester-amide)s produced from the melt polycondensation of BHETA and renewable diesters in the presence of $Ti(O^{i}Pr)_4$.

6. Polycarbonate Degradation

6.1. BPA-PC Methanolysis

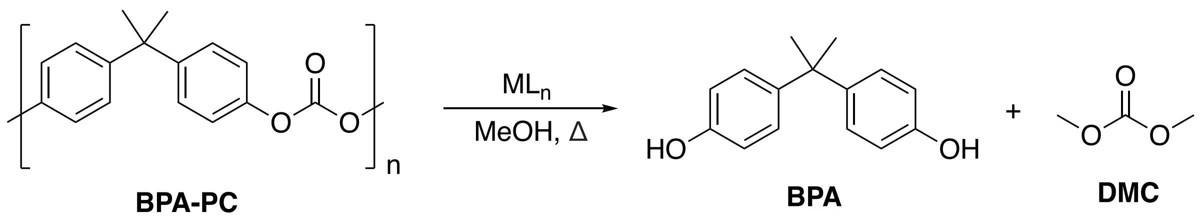

Scheme S8. Metal-mediated methanolysis of BPA-PC into BPA and DMC.

General procedure: A J Young's ampoule was charged with BPA-PC pellets (0.25 g, M_w ~ 45,000 g mol^{-1}) and metal complex (1 - 8 wt%, 0.0025 - 0.02 g, 0.32 – 2.5 mol% relative to carbonate linkages) in a glovebox filled with argon. 2-Me-THF (4 mL) and a desired amount of MeOH (0.21 – 1 mL, 5 – 25 equiv.) were added under a dynamic flow of argon and the ampoule was submerged in a pre-heated oil bath (50 °C), observing complete polymer dissolution within 15 to 30 minutes. Sample aliquots were taken under a flow of argon and analyzed by ^{1}H NMR ($CDCl_3$) spectroscopy.

General procedure for BPA isolation: Following reaction completion, diethyl ether (10 mL) was added and the volatile components were removed *in vacuo* to afford a crude white solid, which was recrystallized from hot deionized H_2O. The resulting solid was isolated by Buchner filtration and dried *in vacuo* at 90 °C for 3 h prior to afford a white powder.

Table S6. Additional BPA-PC methanolysis data using Zn(**2**)$_2$ and Zn(**2**)Et.

Catalyst	Time / h	T / °C	Catalyst loading / wt%	Alcohol / equiv.	S_{BPA} / %	S_{OC} / %
Zn(**2**)$_2$	0.5[a]	75	4	25	80	-
	1[a]	50	4	25	68	-
	1[b]	50	4	10	87	76
	4	25	4	25	82	73
	4	25	4	10	85	79
Zn(**2**)Et	2[c]	50	4	10	88	84
	1[d]	50	4	10	94	85
	1[b]	50	4	10	96	86
	1[e]	50	4	10	93	84
	1[f]	50	4	10	0	0
	2	25	4	10	91 [85]	80
	2[g]	25	4	10	88 [89]	75
$Zn(OAc)_2 \cdot 2H_2O$	1[h]	50	4	10	0	0
HCl	4[i]	25	1[j]	10	0	0

Reaction conditions: BPA-PC pellets (0.25 g, M_w ~ 45,000 g mol^{-1}), 10 – 25 equiv. (0.41 – 1 mL) of MeOH (relative to carbonate linkages), Zn(II)-complexes = 4 wt% (0.01 g, 1.3 – 2.2 mol% relative to carbonate linkages), $Zn(OAc)_2 \cdot 2H_2O$ = 4 wt% (0.01 g, 4.4 mol% relative to carbonate linkages), solvent: 2-Me-THF (4 mL). S_{BPA} and S_{OC} refer to BPA and organic carbonate selectivity respectively. Bracketed values correspond to isolated yields. Unless otherwise stated, complete BPA-PC consumption was observed. For reactions where incomplete polymer

dissolution was observed, BPA-PC was recovered by filtration and dried at 140 °C to constant weight. [a] Solvent: dimethyl carbonate (DMC). [b] Mixed feed: BPA-PC pellets (0.25 g, M_w ~ 45,000 g mol^{-1}) + bottle-grade PET (0.25 g, M_n ~ 40000 g mol^{-1}) + PVC (0.025 g, 10 wt%, M_n = 22000 g mol^{-1}). [c] Ethanolysis, yielding diethyl carbonate (DEC). [d] Mixed feed: BPA-PC pellets (0.25 g, M_w ~ 45,000 g mol^{-1}) + bottle-grade PET (0.25 g, M_n ~ 40000 g mol^{-1}). [e] Compact disc (CD) (0.25 g). [f] Solvent-free. Incomplete BPA-PC consumption, m(BPA-PC recovered) = 0.2240 g, corresponding to 10% depolymerization by weight. [g] Mixed feed scale-up: BPA-PC pellets (2.5 g, M_w ~ 45,000 g mol^{-1}) + bottle-grade PET (2.5 g, M_n ~ 40000 g mol^{-1}). [h] Incomplete BPA-PC consumption, m(BPA-PC recovered) = 0.2460 g, corresponding to 2% depolymerization by weight. [i] Incomplete BPA-PC consumption, m(BPA-PC recovered) = 0.2526 g, corresponding to 0% depolymerization by weight. [j] Molar ratio of [catalyst]/[BPA-PC].

6.1.1. Degradation Kinetics

Reaction kinetic analysis was performed as described previously by Song and co-workers.[6] A total of 4 data points were obtained in equal intervals prior to the complete consumption of BPA-PC using the methanolysis method detailed in section 6.1. After the desired amount of time elapsed, non-depolymerized BPA-PC was recovered by filtration and dried at 140 °C for 1 h or until constant weight was achieved. The pseudo-first-order rate constant (k_{app}) was determined by plotting ln(1/1-X) against time, where X corresponds to the conversion of BPA-PC, equating to depolymerization by weight at a reaction time t.

Table S7. BPA-PC methanolysis using Zn(**2**)$_2$ and Zn(**2**)Et in 2-MeTHF at room temperature.

Catalyst	Time / h	T / °C	Catalyst loading / wt%	S_{BPA} / %	k_{app} / min^{-1}
Zn(**2**)$_2$	4	25	4	85	0.28 ± 0.040
Zn(**2**)Et	2	25	4	91	0.47 ± 0.049

Reaction conditions: BPC-PC pellets (0.25 g, M_w ~ 45000 g mol^{-1}), $n_{carbonate}$:n_{MeOH} = 1:10, Zn(II)-complex = 4 wt% (0.01 g, 1.3 – 2.2 mol% relative to carbonate linkages), solvent: 2-MeTHF (4 mL).

6.1.2. Representative ^{1}H NMR Spectra

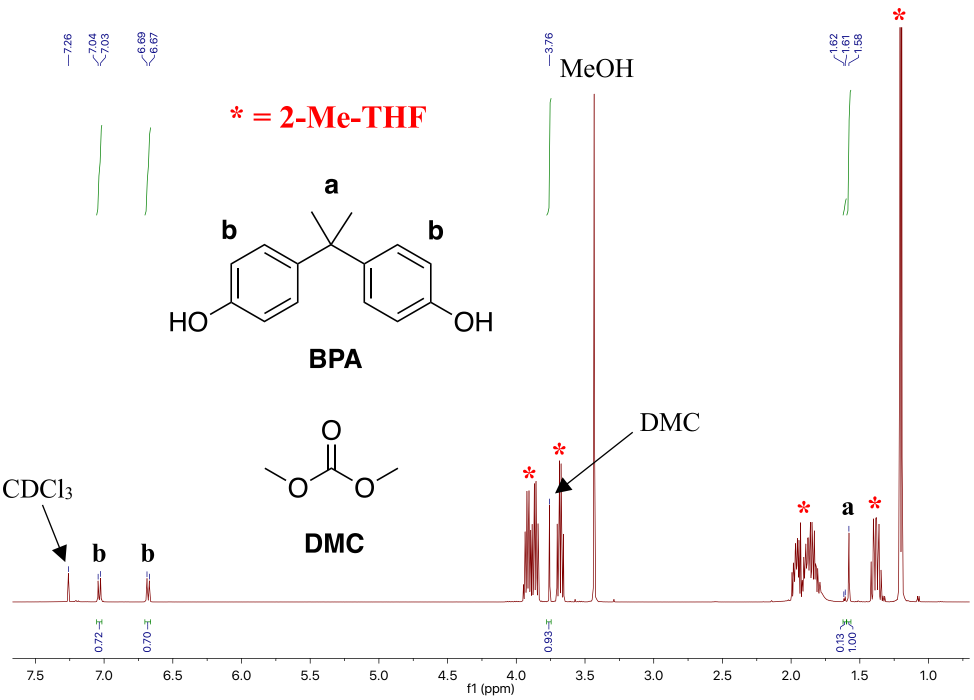

Figure S72. ^{1}H NMR ($CDCl_3$, 500 MHz) spectrum of BPA-PC (M_w ~ 45,000 g mol^{-1}) methanoylsis (10 equiv. MeOH) reaction aliquot after 1 h at 50 °C using Zn(**2**)$_2$ (4 wt% cat. loading, 1.3 mol% relative to carbonate linkages) in 2-Me-THF. ^{1}H NMR peak assignments based on literature example.[7] Selectivity calculated based on methyl region at δ = 1.60 ppm (**a**).

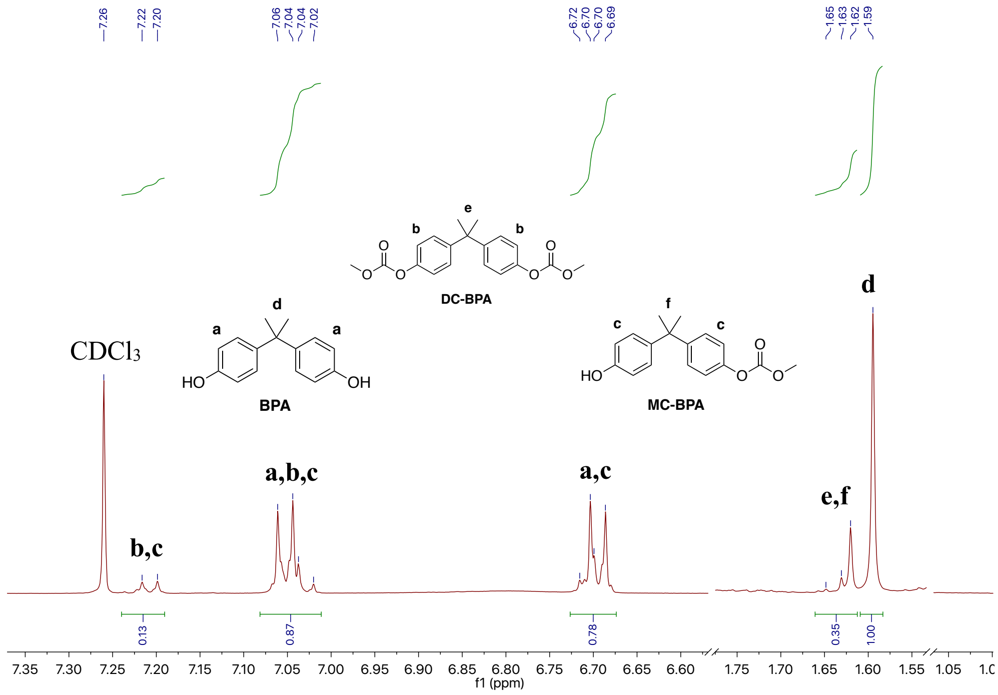

Figure S73. ^{1}H NMR ($CDCl_3$, 500 MHz) spectrum of BPA-PC (M_w ~ 45,000 g mol^{-1}) methanolysis (10 equiv. MeOH) reaction aliquot after 30 mins at 50 °C using Zn(**2**)$_2$ (4 wt% cat. loading, 1.3 mol% relative to carbonate linkages) in 2-MeTHF. ^{1}H NMR peak assignments based on literature example.[7] Selectivity calculated based on methyl region at δ = 1.60 ppm (**d**). Signals from carbonates (MC-BPA and DC-BPA) overlap with solvent resonances.

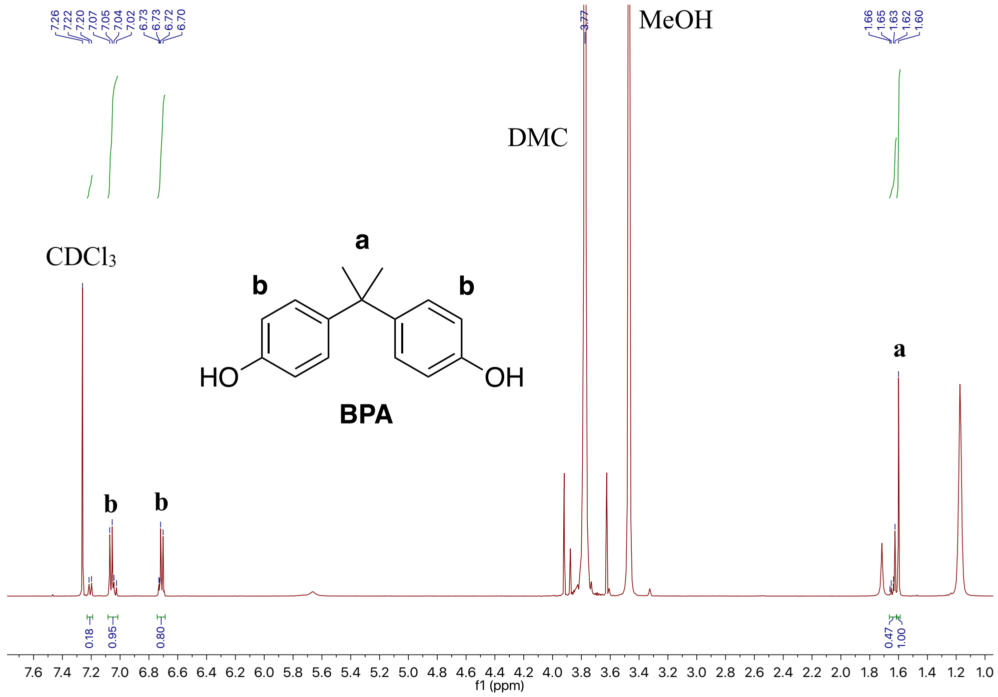

Figure S74. ^{1}H NMR ($CDCl_3$, 500 MHz) spectrum of BPA-PC (M_w ~ 45,000 g mol^{-1}) methanolysis (25 equiv. MeOH) reaction aliquot after 1 h at 50 °C using Zn(**2**)$_2$ (4 wt% cat. loading, 1.3 mol% relative to carbonate linkages) in DMC. ^{1}H NMR peak assignments based on literature example.[7] Selectivity calculated based on methyl region at δ = 1.60 ppm (**a**). Signals from carbonates (MC-DPA, DC-BPA and DMC) overlap with solvent resonances.

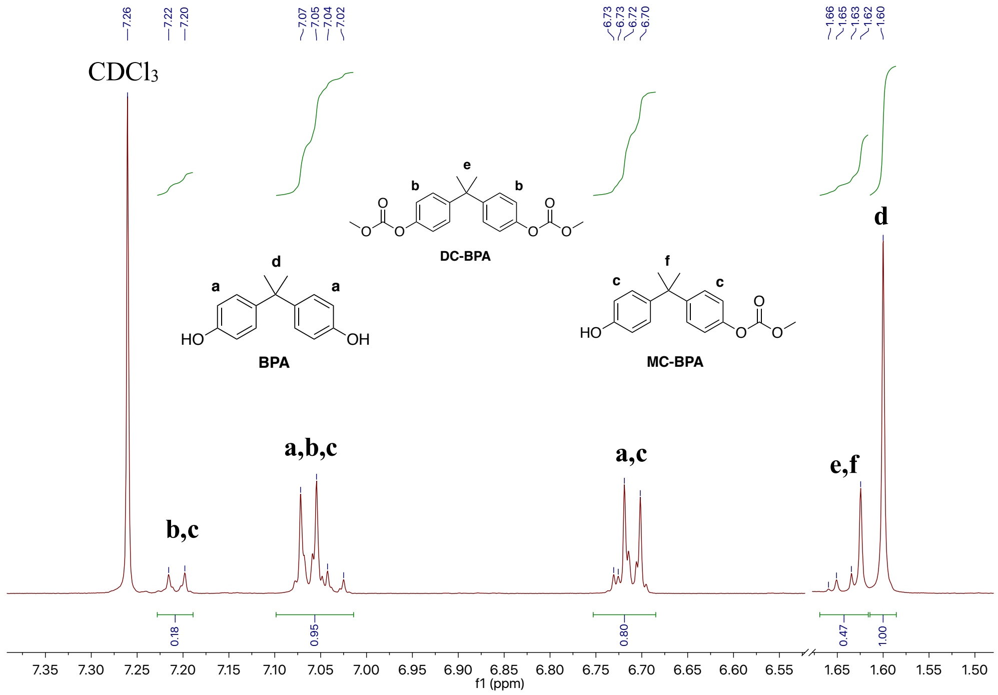

Figure S75. Magnified ^{1}H NMR ($CDCl_3$, 500 MHz) spectrum of BPA-PC (M_w ~ 45,000 g mol^{-1}) methanolysis (25 equiv. MeOH) reaction aliquot after 1 h at 50 °C using Zn(**2**)$_2$ (4 wt% cat. loading, 1.3 mol% relative to carbonate linkages) in DMC, highlighting carbonate product distribution. ^{1}H NMR peak assignments based on literature example.[7] Selectivity calculated based on methyl region at δ = 1.60 ppm (**d**). Signals from carbonates (MC-DPA, DC-BPA and DMC) overlap with solvent resonances.

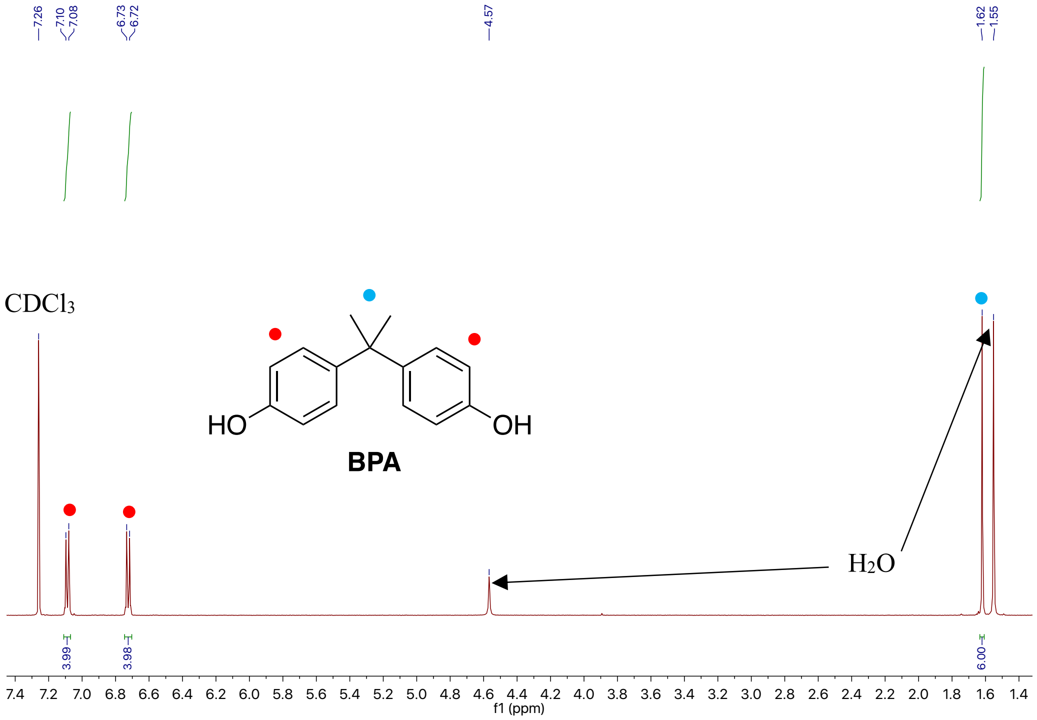

Figure S76. ^{1}H NMR ($CDCl_3$, 500 MHz) spectrum of recrystallized BPA from BPA-PC methanolysis (10 equiv. MeOH) reaction using Zn(**2**)Et (4 wt% cat. loading, 2.2 mol% relative to carbonate linkages) in 2-MeTHF at RT for 2 h.

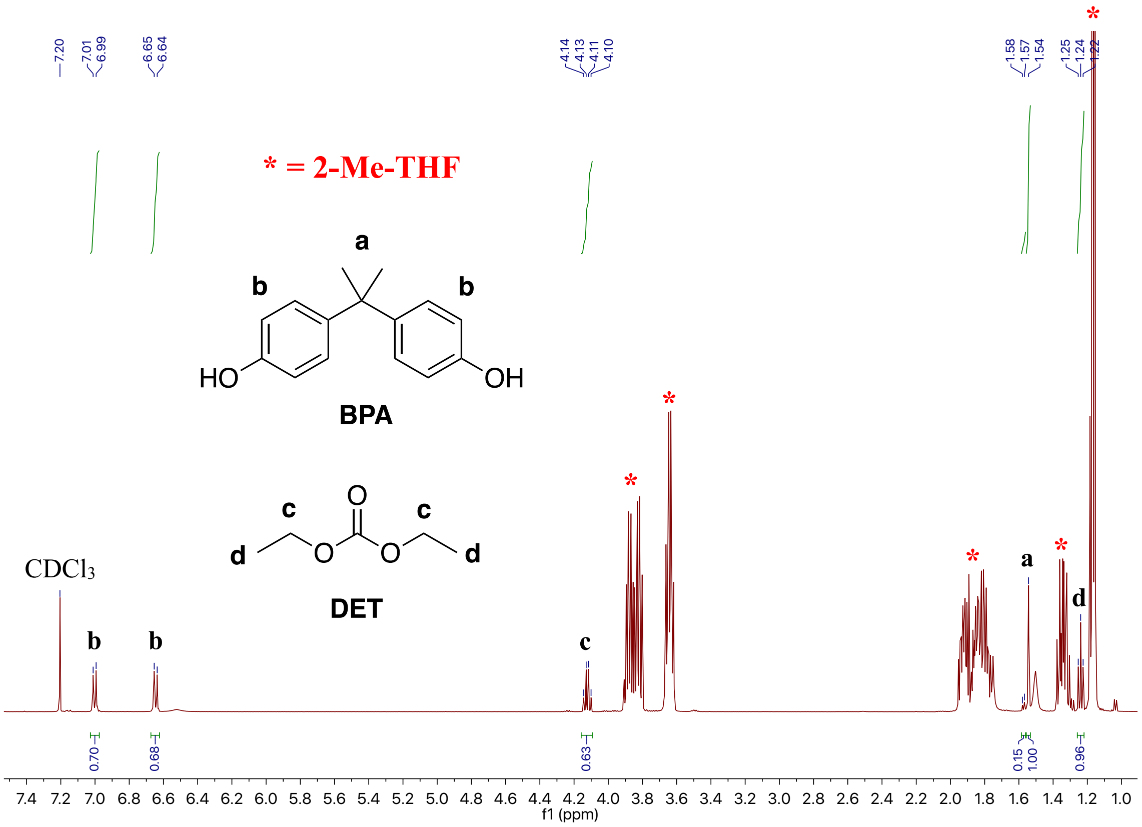

Figure S77. ^{1}H NMR ($CDCl_3$, 500 MHz) spectrum of BPA-PC (M_w ~ 45,000 g mol^{-1}) ethanolysis (10 equiv. EtOH) reaction aliquot after 2 h at 50 °C using Zn(**2**)Et (4 wt% cat. loading, 2.2 mol% relative to carbonate linkages) in 2-MeTHF. Selectivity calculated based on methyl region at δ = 1.60 ppm (**a**).

6.2. BPA-PC Glycolysis

Cyclic carbonates derived from BPA-PC glycolysis were prepared in accordance to methods described previously by Jehanno *et al.*[8]

General procedure: A J Young's ampoule was charged with BPA-PC pellets (0.25 g, M_w ~ 45,000 g mol^{-1}) and Zn(**2**)Et catalyst (4 wt% cat. loading, 0.01 g, 2.2 mol% relative to carbonate linkages) in a glovebox filled with argon. 2-Me-THF (4 mL) and the desired amount of glycol (1.5 – 10 equiv.) were added under a dynamic flow of argon and the ampoule was submerged into a pre-heated oil bath (75 °C). Sample aliquots of the crude (50 µL) were taken under a flow of argon and analyzed by ^{1}H NMR (D_6-DMSO) spectroscopy. Tetramethylsilane (TMS, 10 µL, 73.5 µmol) was employed as an internal standard for calculating conversion.

Table S8. BPA-PC glycolysis using Zn(**2**)Et.

Catalyst	Time / h	T / °C	Catalyst loading / wt%	Glycol	Glycol / equiv.	S_{BPA} / %	S_{CC} / %
Zn(**2**)Et	1	75	4	*rac*-butane-1,3-diol	10	96	78
	1	75	4	2,2-dimethyl-1,3-propanediol	5	99	12
	4	75	4		5	99	7
	1	75	4		1.5	96	48
	4	75	4		1.5	96	26

Reaction conditions: BPC-PC pellets (0.25 g, M_w ~ 45000 g mol^{-1}), solvent: 2-Me-THF (4 mL), 1.5 – 10 equiv. (0.15 – 0.89 g) of glycol (relative to carbonate linkages), Zn(**2**)Et = 4 wt% (0.01 g, 2.2 mol% relative to carbonate linkages). S_{BPA}, S_{CC} and S_{LC} refer to BPA, cyclic carbonate and linear carbonate respectively.

6.2.1. 4-Methyl-1,3-dioxan-2-one

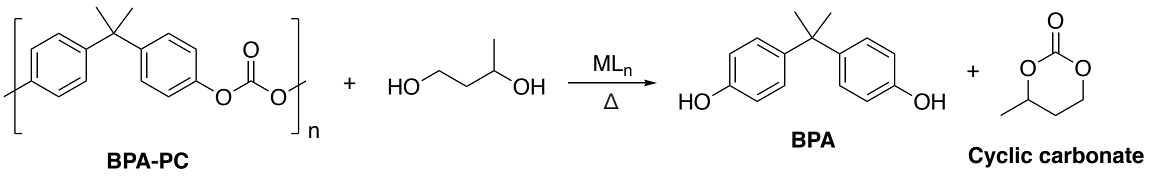

Scheme S9. Metal-mediated BPA-PC glycolysis for cyclic carbonate production using *rac*-butane-1,3-diol (0.88 mL, 10 equiv.) and the general procedure detailed in section 7.2.

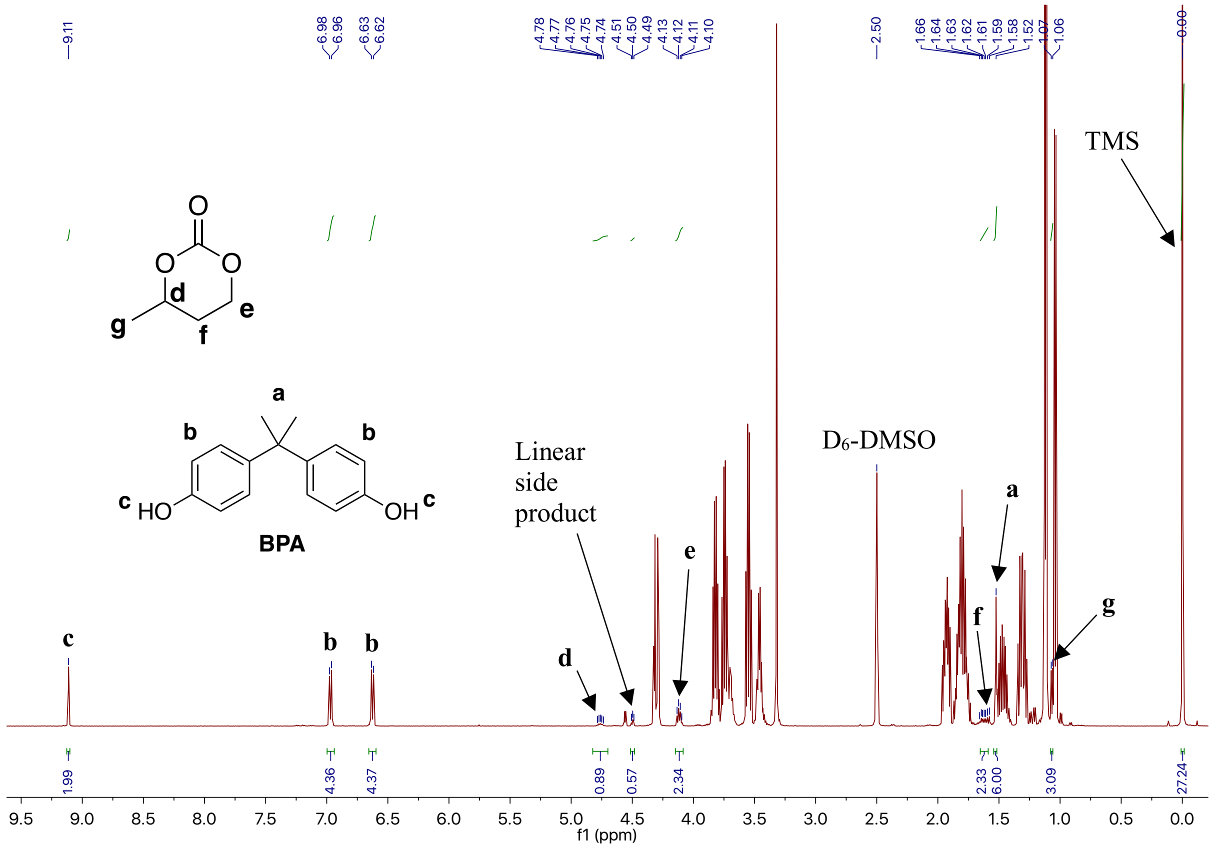

Figure S78. ¹H NMR (D_6-DMSO, 500 MHz) of BPA-PC (M_w ~ 45,000 g mol^{-1}) degradation reaction aliquot (50 μL) after 1 h at 75 °C using *rac*-butane-1,3-diol (0.88 mL, 10 equiv.) in the presence of Zn(**2**)Et (4 wt% cat. loading, 0.01 g, 2.2 mol% relative to carbonate linkages) in 2-MeTHF. ¹H NMR peak assignments based on literature example.[8] Tetramethylsilane (TMS, 10 μL, 73.5 μmol) was employed as an internal standard for calculating conversion. *N.B.* Unassigned peaks can be attributed to residual *rac*-butane-1,3-diol. In accordance to work by Jehanno *et al.*[8], the additional triplet at δ = 4.50 ppm can likely be attributed to a linear side product arising from the attack of a second nucleophile on the active carbonate species, which competes with ring-closure.

6.2.2. 5,5-Dimethyl-1,3-dioxan-2-one

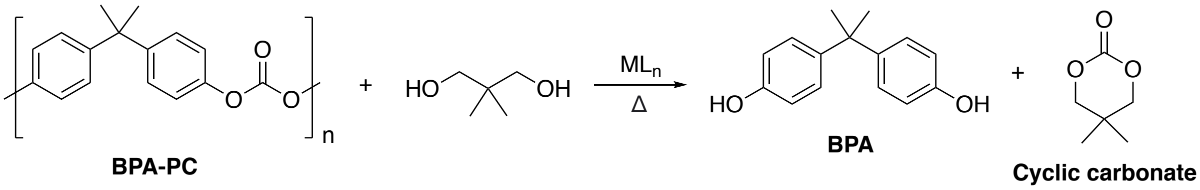

Scheme S10. Metal-mediated BPA-PC glycolysis for cyclic carbonate production using 2,2-dimethyl-1,3-propanediol (0.15 – 0.51 g, 1.5 - 5 equiv.) and the general procedure detailed in section 7.2.

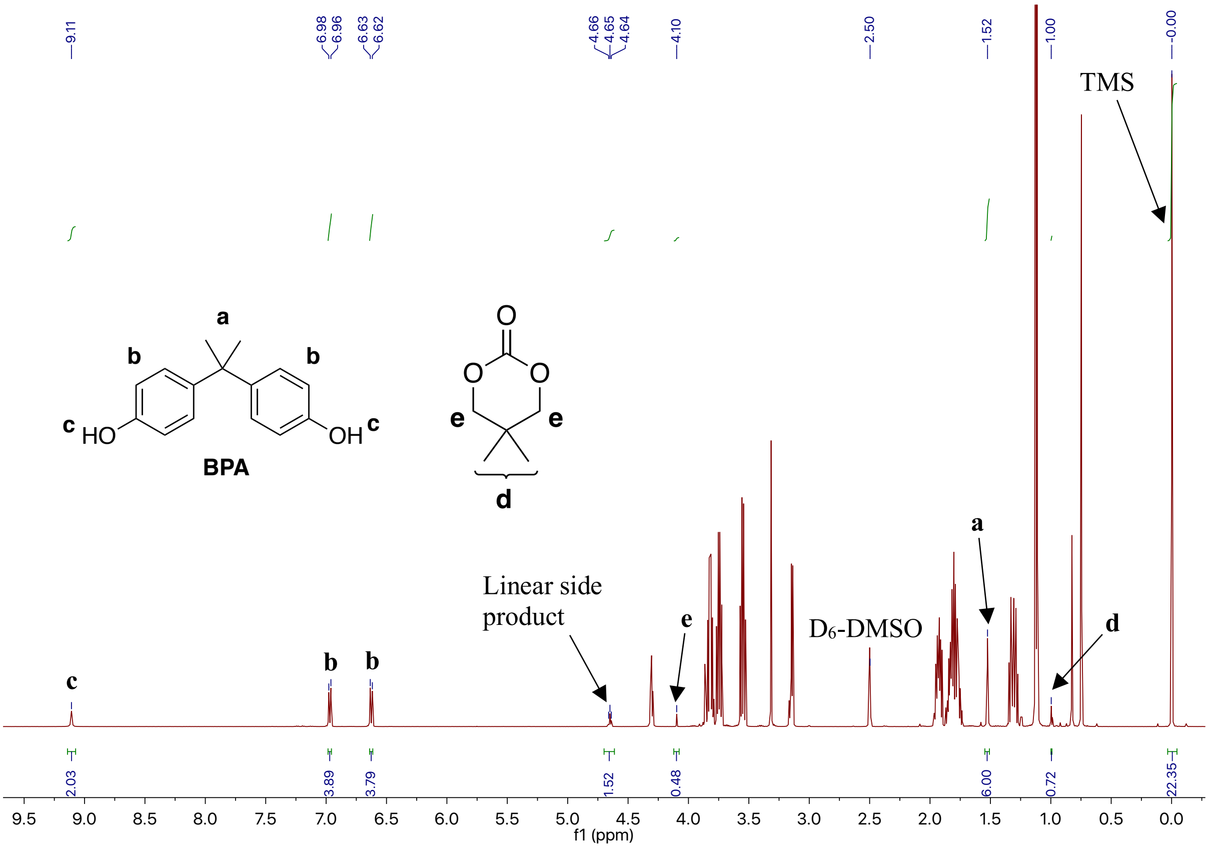

Figure S79. ^{1}H NMR (D_6-DMSO, 500 MHz) of BPA-PC (M_w ~ 45,000 g mol^{-1}) degradation reaction aliquot (50 µL) after 1 h at 75 °C using 2,2-dimethyl-1,3-propanediol (0.51 g, 5 equiv.) in the presence of Zn(**2**)Et (4 wt% cat. loading, 0.01 g, 2.2 mol% relative to carbonate linkages) in 2-MeTHF. ^{1}H NMR peak assignments based on literature example.[8] Tetramethylsilane (TMS, 10 µL, 73.5 µmol) was employed as an internal standard for calculating conversion. *N.B.* Unassigned peaks can be attributed to residual 2,2-dimethyl-1,3-propanediol. In accordance to work by Jehanno *et al.*[8], the additional triplet at δ = 4.65 ppm can likely be attributed to a linear side product arising from the attack of a second nucleophile on the active carbonate species, which competes with ring-closure.

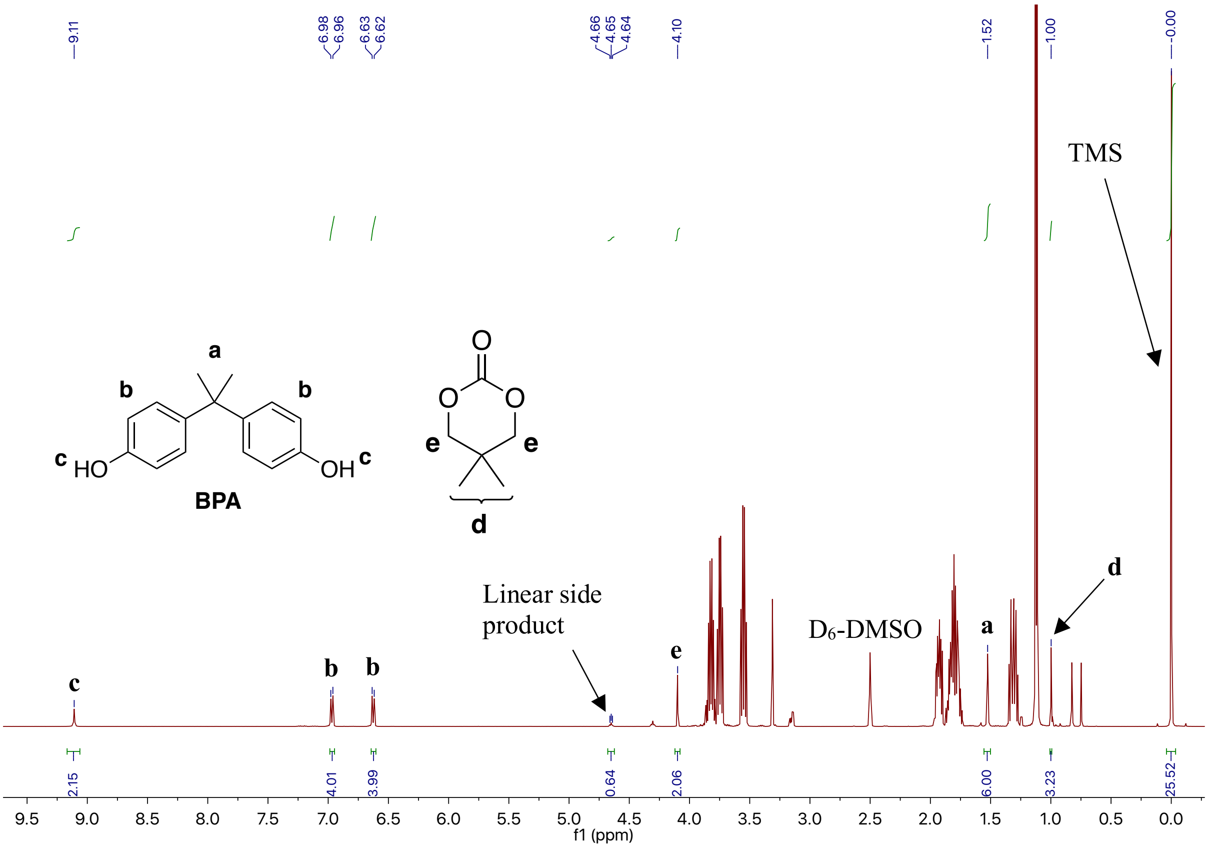

Figure S80. ^{1}H NMR (D_6-DMSO, 500 MHz) of BPA-PC (M_w ~ 45,000 g mol^{-1}) degradation reaction aliquot (50 µL) after 1 h at 75 °C using 2,2-dimethyl-1,3-propanediol (0.15 g, 1.5 equiv.) in the presence of Zn(**2**)Et (4 wt% cat. loading, 0.01 g, 2.2 mol% relative to carbonate linkages) in 2-MeTHF. ^{1}H NMR peak assignments based on literature example.[8] Tetramethylsilane (TMS, 10 µL, 73.5 µmol) was employed as an internal standard for calculating conversion. *N.B.* Unassigned peaks can be attributed to residual 2,2-dimethyl-1,3-propanediol. In accordance to work by Jehanno *et al.*[8], the additional triplet at δ = 4.65 ppm can likely be attributed to a linear side product arising from the attack of a second nucleophile on the active carbonate species, which competes with ring-closure.

6.3. PPC Methanolysis

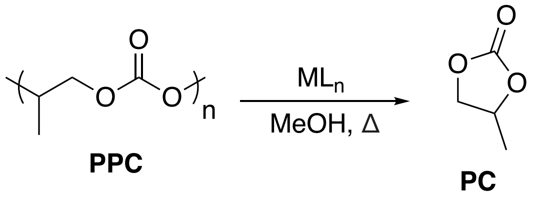

Scheme S11. Metal-mediated methanolysis of PPC into propylene carbonate (PC).

General procedure: A J Young's ampoule was charged with PPC pellets (0.25 g, M_n ~ 50,000 g mol^{-1}) and metal complex (4 wt% cat. loading, 0.01 g, 0.51 – 0.88 mol% relative to carbonate linkages) in a glovebox filled with argon. THF (4 mL) was added under a dynamic flow of argon and the ampoule submerged into a pre-heated oil bath (50 °C), achieving a homogeneous solution within 30 minutes, with heat and stirring assisting polymer dissolution. A desired amount of MeOH (0.15 – 1 mL, 1.5 – 10 equiv.) was then added and the solution stirred for a predetermined amount of time (1 – 1.5 h), after which an aliquot of the crude (50 µL) was taken for ^{1}H NMR ($CDCl_3$) spectroscopic analysis. Tetramethylsilane (TMS, 10 µL, 73.5 µmol) was employed as an internal standard for calculating conversion.

Table S9. PPC methanolysis using Zn(**2**)$_2$ and Zn(**2**)Et.

Catalyst	Time / h	T / °C	Catalyst loading / wt%	MeOH / equiv.	S_{PC} / %	PPC consumption / %
No cat.	1.5	50	4	1.5	0	0
Zn(**2**)$_2$	1.5	50	4	1.5	58	100
Zn(**2**)Et	1	50	4	10	38	100
	1	50	4	5	39	100
	1.5	50	4	1.5	50	100

Reaction conditions: 0.25 g PPC pellets (M_n ~ 50000 g mol^{-1}), solvent: THF (4 mL), 1.5 – 10 equiv. (0.15 – 0.5 mL) of MeOH (relative to carbonate linkages), Zn(II)-complex = 4 wt% (0.01 g, 0.51 – 0.88 mol% relative to carbonate linkages). S_{PC} refers to propylene carbonate selectivity. *N.B*. All reaction times refer to the time taken to achieve complete PPC consumption.

6.3.1. Representative ^{1}H NMR Spectra

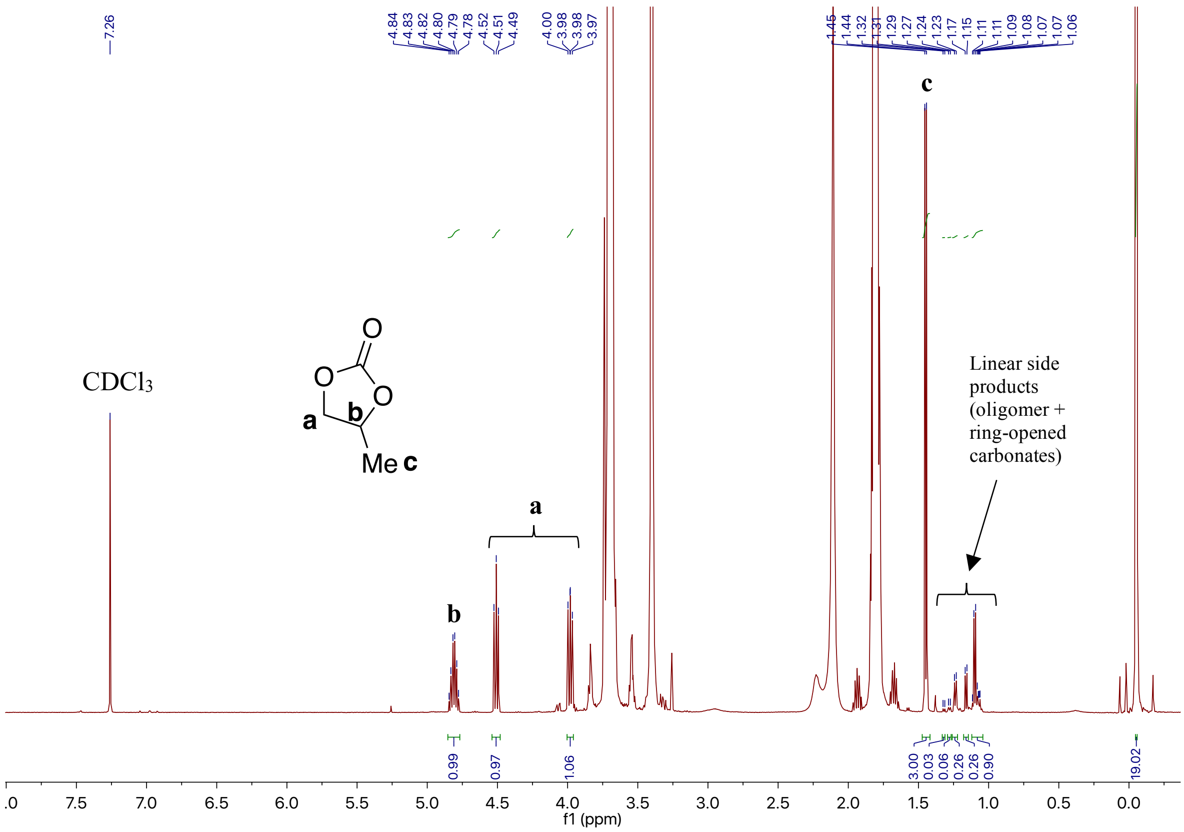

Figure S81. ^{1}H NMR ($CDCl_3$, 500 MHz) of PPC (M_n ~ 50,000 g mol^{-1}) methanolysis (10 equiv. MeOH) reaction aliquot (50 µL) after 1 h at 50 °C using Zn(**2**)Et (4 wt% cat. loading, 0.01 g, 0.88 mol% relative to carbonate linkages). ^{1}H NMR peak assignments based on literature example.[9] Tetramethylsilane (TMS, 10 µL, 73.5 µmol) was employed as an internal standard for calculating conversion. *N.B.* Unassigned peaks can be attributed to excess MeOH and solvent (THF). Additional peaks between δ = 1.06 – 1.23 ppm can likely be attributed to linear side products, including oligomer and ring-opened carbonates.

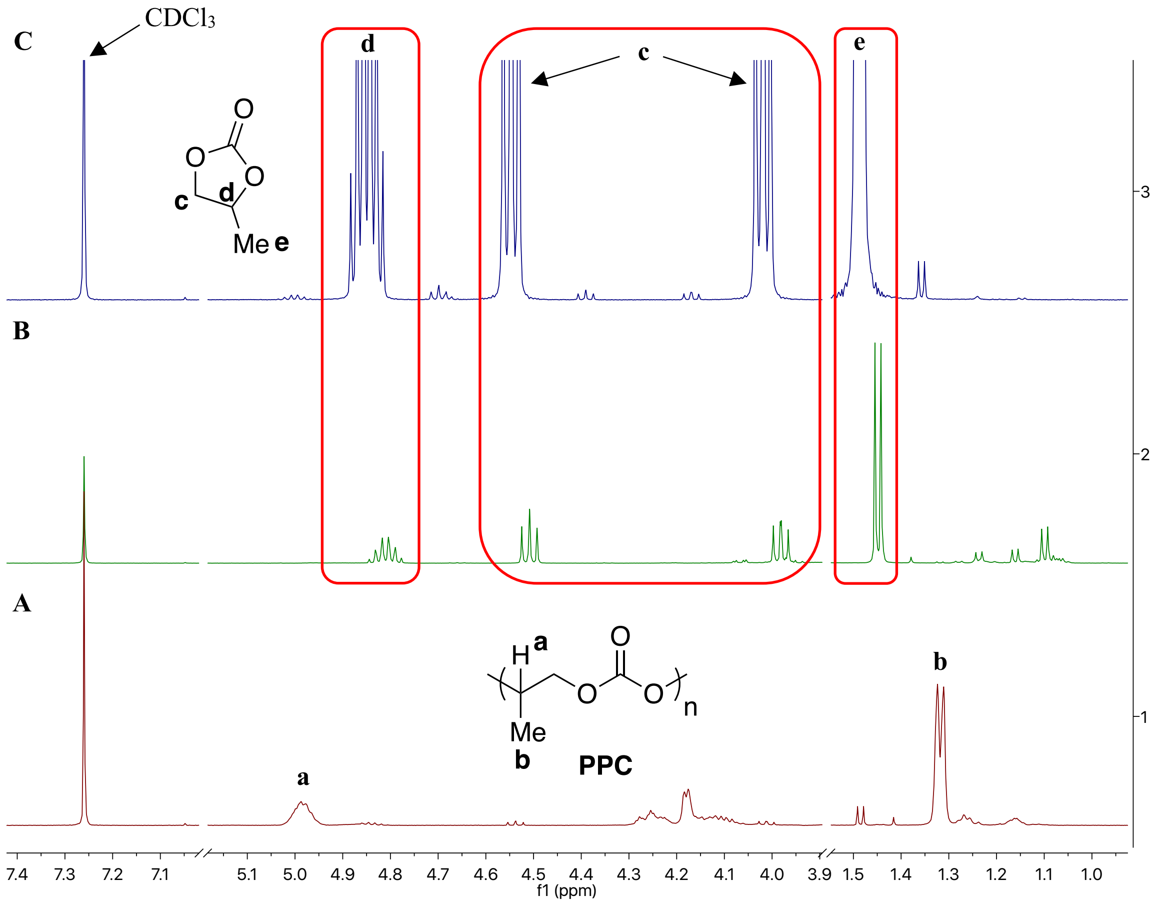

Figure S82. Stacked ^{1}H NMR ($CDCl_3$, 500 MHz) spectra assessing PPC consumption and PC formation: (**A**) Neat PPC (M_n ~ 50,000 g mol^{-1}) in THF, (**B**) PPC (M_n ~ 50,000 g mol^{-1}) methanolysis (10 equiv. MeOH) reaction aliquot (50 µL) after 1 h at 50 °C using Zn(**2**)Et (4 wt% cat. loading, 0.01 g, 0.88 mol% relative to carbonate linkages), and; (**C**) Neat PC. *N.B.* PPC consumption determined by ^{1}H NMR ($CDCl_3$) analysis of the methine (δ = 4.99 ppm, **a**).

7. Crystallographic Data

Special refinement details include:

[Zn(**3**)Et]$_2$: The complex has a centre of inversion to complete the macrocycle.

Table S10. Crystallographic data for Zn(**2**)$_2$, Zn(**2,4-5**)Et and [Zn(**3**)Et]$_2$. **CCDC 2126583-2126587.**

**Compound reference**	Zn(**2**)$_2$	Zn(**2**)Et	[Zn(**3**)Et]$_2$	Zn(**4**)Et	Zn(**5**)Et
Empirical formula	$C_{24}H_{34}Br_4N_4O_2Zn$	$C_{14}H_{22}Br_2N_2O_1Zn$	$C_{35}H_{56}N_4O_2Zn_2$	$C_{21}H_{38}N_2OZn$	$C_{13}H_{20}Br_2N_2O_1Zn$
Formula Mass	795.56	459.52	695.57	399.90	445.50
Crystal system	Triclinic	Orthorhombic	Triclinic	Monoclinic	Orthorhombic
a/Å	11.3914(5)	9.8615(3)	9.7149(9)	12.4990(4)	13.7352(2)
b/Å	12.0168(7)	16.0324(8)	10.0750(12)	10.9531(4)	16.3456(3)
c/Å	12.9805(9)	21.1643(11)	10.6705(10)	15.9078(7)	14.5299(2)
α/°	109.286(5)	90	79.627(9)	90	90
β/°	98.468(5)	90	69.221(9)	96.302(3)	90
γ/°	116.077(5)	90	65.761(11)	90	90
Unit cell volume/Å^3	1413.97(16)	3346.2(3)	889.66(18)	2164.66(14)	3262.11(9)
Temperature/K	150.00(10)	150.01(10)	150.00(10)	150.01(10)	150.00(10)
Space group	P-1	Pbca	P-1	P21/c	Pbcn
No. of formula units per unit cell, Z	2	8	1	4	8
Radiation type	Cu Kα	Cu Kα	Cu Kα	Cu Kα	Cu Kα
No. of reflections measured	8669	40504	12022	28971	65654
No. of independent reflections	5002	3171	3239	3951	3281
R_{int}	0.0343	0.0368	0.0379	0.0275	0.0490
Final R_1 values ($I > 2\sigma(I)$)	0.0424	0.0190	0.0246	0.0242	0.0466
Final $wR(F^2)$ values ($I > 2\sigma(I)$)	0.1103	0.0449	0.0655	0.0671	0.1124
Final R_1 values (all data)	0.0477	0.0206	0.0253	0.0253	0.0482
Final $wR(F^2)$ values (all data)	0.1156	0.0456	0.0660	0.0681	0.1138

Selected bond lengths and angles:

Table S11. Selected bond lengths for Zn(**2**)$_2$, Zn(**2,4-5**)Et and [Zn(**3**)Et]$_2$.

**Catalyst**	**Bond**	**Bond length / Å**
Zn(**2**)$_2$	Zn(1)-N(1) {NH}, Zn(1)-N(2) {NMe_2}, Zn(1)-N(3) {NH}	2.104(3), 2.109(3), 2.120(3)
Zn(**2**)Et	Zn(1)-N(1) {NH}, Zn(1)-N(2) {NMe_2}	2.1073(16), 2.1721(16)
[Zn(**3**)Et]$_2$		2.1043(13), 2.1631(13)
Zn(**4**)Et	Zn(1)-N(1) {NH}, Zn(1)-N(2) {NHMe}	2.1204(11), 2.1352(11)
Zn(**5**)Et		2.119(3), 2.086(4)

Table S12. Selected bond angles for Zn(**2**)$_2$, Zn(**2,4-5**)Et and [Zn(**3**)Et]$_2$ with calculated $\tau_{4/5}$ values. Ideal tetrahedral geometry corresponds to $\tau_4 = 1$. Ideal trigonal bipyramidal geometry corresponds to $\tau_5 = 1$.

**Catalyst**	**Bond**	**Bond angle / °**	$\tau_{4/5}$
Zn(**2**)$_2$	O(1)-Zn(1)-O(2), N(1)-Zn(1)-N(3)	165.65(11), 133.00(13)	0.54
Zn(**2**)Et	O(1)-Zn(1)-C(1), C(1)-Zn(1)-N(1)	127.27(7), 117.97(7)	0.81
[Zn(**3**)Et]$_2$		129.66(7), 117.72(7)	0.80
Zn(**4**)Et		124.83(5), 120.93(5)	0.81
Zn(**5**)Et	C(1)-Zn(1)-N(1), C(1)-Zn(1)-N(2)	126.33(16), 117.12(16)	0.83

Crystallographic data is accessible via the Cambridge Crystallographic Data Centre (CCDC). CCDC 2126583-2126587.

References:

1. G. R. Fulmer, A. J. M. Miller, N. H. Sherden, H. E. Gottlieb, A. Nudelman, B. M. Stoltz, J. E. Bercaw, K. I. Goldberg, *Organometallics* **2010**, *29*, 2176-2179.
2. R. Evans, Z. Deng, A. K. Rogerson, A. S. McLachlan, J. J. Richards, M. Nilsson, G. A. Morris, *Angew. Chem. Int. Ed.* **2013**, *52*, 3199-3202.
3. B. M. Chamberlain, M. Cheng, D. R. Moore, T. M. Ovitt, E. B. Lobkovsky, G. W. Coates, *J. Am. Chem. Soc.* **2001**, *123*, 3229-3238.
4. L. A. Román-Ramírez, P. Mckeown, M. D. Jones, J. Wood, *ACS Catal*. **2018**, *9*, 409-416.
5. K. Fukushima, J. M. Lecuyer, D. S. Wei, H. W. Horn, G. O. Jones, H. A. Al-Megren, A. M. Alabdulrahman, F. D. Alsewailem, M. A. McNeil, J. E. Rice, J. L. Hedrick, *Polym. Chem.* **2013**, *4*, 1610−1616.
6. X. Song, W. Hu, W. Huang, H. Wang, S. Yan, S. Yu, F. Liu, *Chem. Eng. J.* **2020**, *388*, 124324.
7. P. McKeown, M. Kamran, M. G. Davidson, M. D. Jones, L. A. Román-Ramírez, J. Wood, *Green Chem.* **2020**, *22*, 3721-3726.
8. C. Jehanno, J. Demarteau, D. Mantione, M. C. Arno, F. Ruipérez, J. L. Hedrick, A. P. Dove, H. Sardon, *ACS Macro Lett.* **2020**, *9*, 443–447.
9. T. Zhou, Z. Zou, J. Gan, L. Chen, M. Zhang, *J. Polym. Res.* ***2011***, 18, 2071-2076.
